# Abiotic sources of fixed nitrogen sustained early ecosystems for several hundred million years after the origin of life

**DOI:** 10.1126/sciadv.aec4450

**Published:** 2026-06-17

**Authors:** Joanne S. Boden, Zhanghan Ni, Rika E. Anderson, Eva E. Stüeken

**Affiliations:** ^1^ School of Earth and Environmental Sciences, University of St. Andrews, Bute Building, Queen’s terrace, St. Andrews, Fife KY16 9TS, UK.; ^2^ School of Geographical Sciences, University of Bristol, Bristol, UK.; ^3^ Department of Biology, Carleton College, Northfield, MN, USA.; ^4^ University of Illinois Urbana-Champaign, Urbana, IL, USA.

## Abstract

Nitrogen (N) plays a crucial role in controlling biological productivity. However, it remains unknown how Earth’s earliest ecosystems accessed bioavailable forms of nitrogen. Here, we present genomic evidence that the last universal common ancestor (LUCA) had genes for importing ammonium into the cell, but the first organisms with all three catalytic nitrogen fixing genes emerged at least 1 billion years later. Similarly, enzymatic pathways for accessing nitrogen from urea and nitriles appear to predate biological N_2_ fixation. Our results imply that Earth’s earliest biosphere was maintained by environmental sources of ammonium and other N-bearing compounds, possibly derived from a combination of processes such as hydrothermal activity, photochemistry, rock weathering, lightning, or impact events. Biological N_2_ fixation may have emerged in response to an increase in biological nutrient demand or due to declining abiotic supplies of ammonium, urea, and nitriles.

## Teaser

Genomic evidence suggests early life relied on ammonium and other N molecules before biological nitrogen fixation evolved.

## INTRODUCTION

Nitrogen (N) is a critical nutrient for all life as we know it and limits primary productivity in large parts of the modern biosphere (*1*, *2*). Today, the major source of bioavailable N (ca. 98%) is biological N_2_ fixation by nitrogenase enzymes. Before the onset of biological N_2_ fixation, abiotic processes must have supplied fixed N to the biosphere. Computational models and experiments have uncovered a variety of potential abiotic N sources that could have been present on early Earth (Table 1). However, the magnitudes of these fluxes are uncertain and difficult to verify. Furthermore, direct evidence for biological utilization of abiotically generated N species is so far lacking. It is therefore unknown for how long these abiotic sources were able to sustain Earth’s early biosphere and if they were limiting primary productivity (*3*). The sedimentary rock record has been used as tentative evidence for abiotic N input to the Eoarchean ocean (*4*), but strong hydrothermal and/or metamorphic alteration render this conclusion uncertain.

**Table 1.** Early Archean fluxes of fixed nitrogen (N) in mol·year^−1^ based on dinitrogen (N_2_) gas as starting material.

Flux type	Magnitude (mol·year^−1^)	Products	Supply rate	References
Lightning	10^9^–10^10^	N oxide(s)*, minor hydrogen cyanide, ammonium, organic compounds including urea	Sporadic	(*4*, *14*, *132*, *133*)
Volcanic eruptions	10^9^–10^11^	N oxide(s)*	Sporadic	(*47*)
Impact shock waves	10^10^–10^11^	N oxide(s)*	Sporadic	(*134*, *135*)
Photochemistry†	10^11^–10^12^	Hydrogen cyanide	Constant	(*52*, *136*)
Hydrothermal vents	10^9^–10^12^	Ammonium	Constant	(*44*, *45*)
Crustal weathering	10^8^–10^9^	Ammonium	Constant	(*46*)
Modern biological N_2_ fixation	10^13^	Ammonium	Constant	(*137*)

* Can undergo abiotic reduction to ammonium, coupled to iron oxidation (*8*).

† Using values with high CH_4_ levels, postdating the origin of life.

The most important inorganic N compound for life on early Earth would likely have been ammonium (NH_4_^+^), which is the preferred form of fixed N for most microbial organisms today (*5*, *6*). Ammonium has the same redox state as N in amino acids, meaning that no redox transformations are required for ammonium incorporation into biomolecules. In its unprotonated form, ammonia (NH_3_) can diffuse passively across cell membranes, but very little NH_3_ exists at cellular pH and in neutral or acidic environments, because the p*K*a (where *K*a is the acid dissociation constant) of the ammonium/ammonia equilibrium is 9.25 (*7*). The efficiency of passive ammonia uptake has also been questioned because although it lacks charges, ammonia is polar (dipole moment of 1.47 D) and may therefore struggle to cross biological membranes without enzymatic support. Perhaps as a result, ammonium is often imported directly from the environment through dedicated uptake enzymes belonging to the Amt/Mep/Rh superfamily of nutrient transporters (*7*). Ammonium can be generated biologically via N_2_ fixation, urea catabolism, or nitrile catabolism. However, before the expansion of biological N_2_ fixation, hydrothermal vents may have been major sources of ammonium on early Earth (Table 1). In addition, N oxides generated during lightning, meteorite impacts, and volcanic eruptions could have been converted into ammonium through reactions with ferrous iron in seawater or on mineral surfaces (*8*, *9*). Once inside microbial cells, the ammonium can be described as “assimilated,” but it does not support biological growth and development until the N inside it is incorporated into amino acids via additional enzymes of the glutamate dehydrogenase pathway or glutamine synthetase glutamate synthase (GOGAT) pathway (*10*). Enzymes of the latter pathway are nearly ubiquitous among prokaryotes, and estimated to date back to the last universal common ancestor (LUCA) (*11*). Therefore, we assume that if an organism had genes to import or generate ammonium, then it could also use that ammonium to support growth and development via GOGAT or GDH pathways.

Other potentially important N compounds are nitriles and urea. Nitriles (molecules with C─N triple bonds), including cyanide, may have been generated by ultraviolet (UV) radiation and in association with impact shock events (see Table 1). This class of compounds plays a prominent role in some origin-of-life models [e.g., (*12*, *13*)]. Urea has been detected in laboratory-based lightning experiments (*14*) and could also have contributed to prebiotic reactions (*13*). If these models are accurate, then one might expect continued biological utilization of nitriles and urea beyond the origin of life. However, no data currently exist to test this idea.

Here, we leverage genomic records of enzymatic ammonium uptake, cyanide catabolism, and urea catabolism through time to determine whether these abiotic N species could have been relevant sources of N before the emergence of biological N_2_ fixation. This approach rests on the assumption that ancient enzymes had the same functions as their modern counterparts, but this assumption is justified by the enzymes’ high substrate specificity. The genomic record thus overcomes some of the uncertainties associated with ancient sedimentary rocks. Our results provide fundamental insights into the ability of planet Earth to sustain its biosphere for the first several hundred million years.

## RESULTS

### Ammonium was bioavailable in the Hadean

**Fig. 1.**
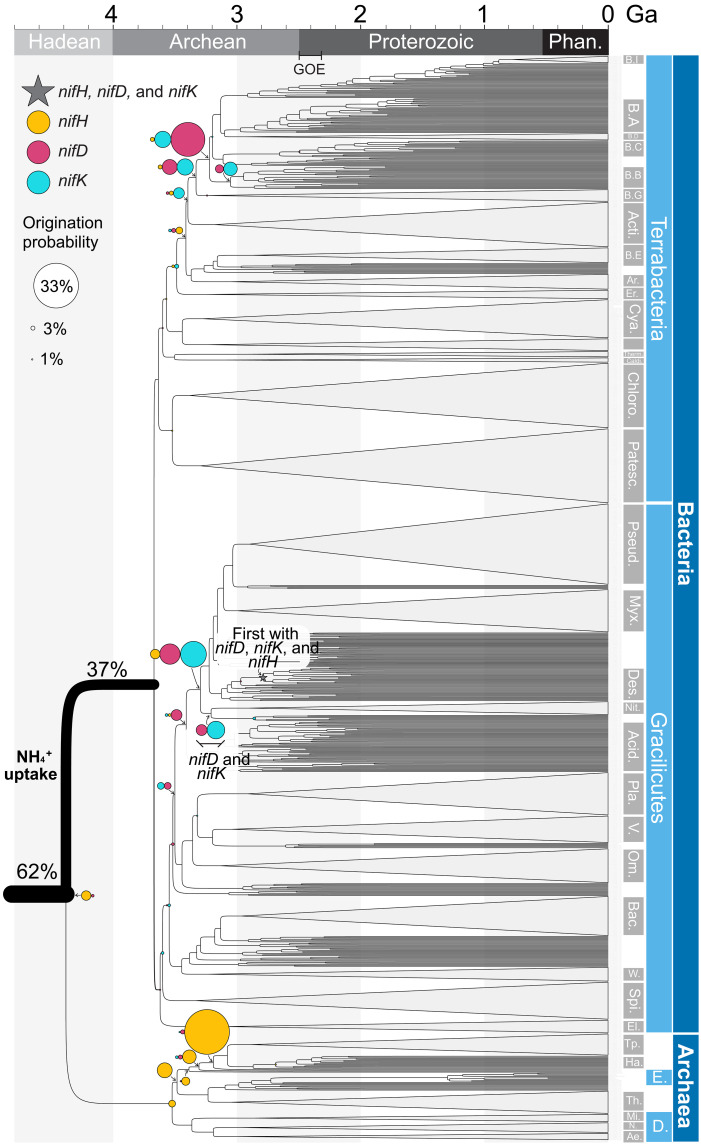
Ammonium-uptake enzymes stem back to LUCA or LBCA, whereas N-fixing enzymes evolved in more recent lineages. The most likely origins of *nifH* (yellow circles), *nifD* (pink circles), and *nifK* (turquoise circles) estimated with the CIR clock model are annotated with colored circles (sized in proportion to origination probability) on a time-calibrated tree of life where branch thicknesses are scaled to represent origination probabilities of ammonium-uptake genes (namely, *amt*/*mep*/*rh*). The grey star and associated annotation indicates the earliest lineage to host all three catalytic N fixing genes (namely, *nifH*, *nifD*, and *nifK*, host is defined as ≥50% presence probability for each gene). Text “*nifD* & *nifK*” indicates the first lineage with genes encoding both N_2_-interacting subunits of nitrogenase (defined as ≥50% presence probabilities of *nifD* and *nifK*). GOE, Great Oxygenation Event; Had., Hadean; Phan., Phanerozoic. Blue rectangles indicate bacterial and archaeal clades (E., Euryarchaeota; D., DPANN) and phyla (from top to bottom: B.I, Bacillota I; B.A, Bacillota A; B.D, Bacillota D; B.C, Bacillota C.; B.B, Bacillota B; B.G, Bacillota G; Acti., Actinobacteriota; B.E, Bacillota E; Ar., Armatimonadota; Er., Eremiobacterota; Cya., Cyanobacteriota; Ma., Margulisbacteriota; Therm., Thermotogota; Caldi., Caldisericota; Chloro., Chloroflexi.; Patesc., Patescibacteriota; Pseud., Pseudomonadota; Myx., Myxococcota; Des., Desulfobacterota; Nit., Nitrospirota; Acid., Acidobacteriota; Pla., Planctomycetota; V., Verrucomicrobiota; Om., Omnitrophota; Bac., Bacteroidota; W., WOR-3; Spi., Spirochaetota; El., Elusimicrobiota; Tp., Thermoplasmatota, Ha., Halobacteriota; Th., Thermoproteota; Mi., Microarchaeota; N., Nanoarchaeota; Ae., Aenigmatarchaeota). The CIR clock model is presented to aid comparison with other studies that use CIR for their main figures.

To estimate when genes for biological ammonium uptake and production emerged and radiated across the tree of life, time-calibrated species trees representing three models of substitution rate inheritance [namely, Cox-Ingersoll-Ross (CIR), lognormal (LN), and uncorrelated gamma multipliers (UGAM)] were reconciled with phylogenetic trees for the primary family of ammonium uptake genes (namely, *amt/mep/rh*) (see Materials and Methods). Estimates made from different clock models vary, with UGAM predicting generally younger species divergences than LN and CIR (fig. S1), but they all estimate with 53 to 62% probability that the LUCA of all life on Earth encoded a gene for *amt*/*mep*/*rh* (Fig. 1 and figs. S2 and S3). LUCA is estimated to have existed at 4.38 to 4.39 billion years ago (Ga) [confidence intervals (CIs) = 4.40 to 4.34 Ga]. This is very close to the oldest evidence for liquid water on the planet’s surface at 4.40 Ga (*15*, *16*), which we applied as an older bound for life. It is also slightly earlier than the date of ~4.2 Ga (CI = 4.33 to 4.09 Ga), which has been estimated for LUCA based on analyses of pre-LUCA gene duplicates (*17*). The second most probable origination of *amt/mep/rh* genes (with a 37 to 46% probability) is in the last bacterial common ancestor (LBCA), estimated to have existed at 4.31 to 3.67 Ga (CIs = 4.39 to 3.51 Ga). Put together, this suggests that fixed N in the form of ammonium and/or methylammonium was being incorporated into early microbial organisms as far back as the Hadean.

### Biological N_2_ fixation post-dates evidence for environmental ammonium

To compare these results with the timing of when microbial communities became capable of fixing N from dinitrogen gas (N_2_), we tracked the rise and spread of the catalytic molybdenum nitrogenase genes (namely, *nifD*, *nifK*, and *nifH*). These genes encode the structural proteins of nitrogenase, with the enzyme encoded by *nifH* acting to shuttle electrons to the nitrogenase catalytic center and active site encoded by *nifD* and *nifK*. All diazotrophic organisms studied to date encode all three subunits (*18*). Previous work has found that molybdenum nitrogenases arose before iron or vanadium nitrogenases (*19*–*22*). Our results indicate an extremely low probability that catalytic molybdenum nitrogenase genes originated in the same ancestral lineages as ammonium uptake genes (namely, ≤7% origination probability for *nifD*, *nifK*, or *nifH* in LUCA versus ≤4% origination probability for *nifD*, *nifK*, or *nifH* in LBCA) (table S1). Previous phylogenetic analyses have also indicated that LUCA lacked nitrogenase genes (*17*, *23*, *24*). Instead, our results, like others [e.g., (*23*)] show that the three nitrogenase structural proteins (encoded by *nifD*, *nifK*, and *nifH*) are more likely to stem back to more recent lineages (origination probabilities of 95 to 100% for all genes; table S2).

Our models were unable to resolve in which ancestral organisms *nifD*, *nifK*, and *nifH* arose because multiple lineages share high origination probabilities. These include several early Terrabacteria, Gracilicutes, and Archaea (Fig. 1 and figs. S5 and S6). Two of our three clock models (namely, CIR and UGAM) suggest that *nifH* is most likely to have originated in Archaea (origination probabilities total 66 to 81% for Archaea versus 14 to 27% for Bacteria). Whereas all of our analyses suggest that *nifK* and *nifD* are more likely to have originated in Bacteria (origination probabilities total 91 to 100% for Bacteria versus up to 7% for Archaea except for *nifD* with UGAM that has a 54% probability of originating in Bacteria compared to a 46% probability of originating in Archaea) (table S1 and Supplementary Text).

### Timing of nitrogenase origination

It is challenging to discern the precise origin of nitrogenase given that it consists of several subunits with different distributions across the tree of life. For example, although *nifD* and *nifK* are generally found in the same species (84 species host both genes, whereas only two species host one of the two genes), *nifH* is found alone in additional species (18 in these analyses; data file S1), as has been noted elsewhere (*21*, *25*). By reconstructing individual gene trees for *nifH*, *nifK*, and *nifD*, we find evidence that some *nifH* homologs evolved along different evolutionary trajectories to other nitrogenase subunits because the topology of our bayesian *nifK* and *nifD* phylogenies are similar (figs. S2 and S3) and different to that of the *nifH* phylogeny (fig. S4). Perhaps for this reason, our results often find high origination probabilities for both *nifD* and *nifK*, but not *nifH* in the same lineages (Fig. 1 and figs. S5 and S6). Both *nifD* and *nifK* genes are known to have evolved from a shared ancestor that duplicated to create two genes. One gene became *nifK*, while the other became *nifD* (*21*, *24*, *26*), but the two genes were dated separately for this analysis. The first lineage to host the two N_2_-interacting components of nitrogenase encoded by *nifD* and *nifK* (defined as a ≥50% presence probability of both genes) was an ancestral bacterium [likely the most recent common ancestor (MRCA) of Nitrospirota and Nitrospinota] that existed in the late Paleo- or Mesoarchean at 3.37, 3.21, or 2.81 Ga (CIs = 3.49 to 2.31 Ga; table S3).

All modern diazotrophic microbes fix N with all three structural subunits of nitrogenase (*nifD*, *nifK*, and *nifH*), but the first microbial lineage to encode all three catalytic *nif* genes (defined as a >50% presence probability for *nifD*, *nifK*, and *nifH*) is predicted to have been an ancestral bacterium (either a Desulfobacteriota or Bacillota E) that existed more recently in geological time, specifically at 2.88 to 1.87 Ga, and potentially as far back as 3.04 Ga (because CIs = 3.04 to 1.70 Ga) (table S4).

### Urea has been bioavailable as a N source since the Mesoarchean

A diverse array of Bacteria, Fungi, and plants extract N from urea using urease enzymes (*27*), which hydrolyse urea into ammonia. The urease enzymes of most bacteria are composed of three subunits, namely, alpha, beta, and gamma encoded by *ureC*, *ureB*, and *ureA* genes, respectively. Accessory genes are required to assemble urease from its subunits and insert nickel cofactors into its active site (*27*) but were not investigated here. In agreement with previous findings [e.g., (*27*)], we find that ureases are present in a wide range of bacteria, including several Cyanobacteria, Pseudomonadota, and Actinobacteria among others, but only one Archaea: *Halorientalis persicus* of the Halobacteriota phylum (data file S1). Specifically, 87 of the 1024 genomes investigated here (equivalent to 8%) encode all three catalytic urease subunits. A further 30 genomes encode two of the three subunits (specifically Beta and Gamma), and a further 33 encode only one subunit (either Gamma or Beta). The first organism most likely to have encoded all three subunits is estimated to have been a Terrabacterium [either the most recent common ancestor of Cyanobacteria and Sericytochromatia (CIR) or the most recent common ancestor of Cyanobacteria and Actinobacteria (UGAM and LN)] that existed between 3.87 and 3.28 Ga (CIs = 3.75 to 3.12 Ga) (table S5).

Genes encoding each individual catalytic subunit probably originated in earlier lineages before they united together in a single genome. This is because there is a 36 to 74% probability of *ureC* originating in a lineage that predates the first lineage to encode all three urease subunits, a 67 to 100% chance of *ureB* originating before the first lineage to encode all three subunits, and a 97 to 100% chance of *ureA* originating before the first lineage to encode all three subunits depending on which clock model is applied to estimate timing. These earlier origins (ignoring lineages with <10% origination probabilities) span from 4.15 to 3.57 Ga for *ureC*, 4.39 to 3.41 Ga for *ureB*, and 4.39 to 3.60 Ga for *ureA*. Whether these ancestral lineages could successfully extract N from urea with only a subset of the three catalytic urease genes remains unknown. Together, our findings indicate that microbial organisms have been extracting N (and carbon) from urea for at least 3.28 Ga and since the start of the Mesoarchean.

### Microbial nitrile catabolism for 3.2 Ga

Nitriles (compounds with C≡N triple bonds, including cyanide) are catabolized by diverse bacteria, fungi, and plants (*28*, *29*). Although their function in bacteria remains largely unknown, nitrilases likely play a role in detoxification (*30*), and the ammonium that is released when nitriles are catabolized (*29*) can serve as a source of N. In line with previous findings [e.g., (*28*, *29*)], we find that class I nitrilase genes are relatively rare (present in 83 of the 1024 genomes analyzed), although widespread in diverse phyla of Bacteria (including Desulfobacterota, Cyanobacteria, and several Bacillota phyla), and several Eukaryotes (data file S1). There is a very low probability that LUCA could degrade nitriles (including cyanide) using class I nitrilases, because origination probabilities in LUCA range from 1% (LN) to 2% (UGAM). Similar results have been reported previously (*28*). Instead, there is a higher probability of nitrile-degrading nitrilases originating in more recent lineages because several have low, but noteworthy origination probabilities ≥10%. Their ages range from 3.82 Ga to 3.14 Ga (CIs span 4.06 to 2.98 Ga; table S6). This predates previous estimates of 1.88 to 1.02 Ga made using alternative molecular clock approaches (*28*). Where Schwartz *et al.* (*28*) implemented relaxed molecular clocks directly onto a gene tree of class I nitrilase enzymes using calibration points from plants, animals, and fungi, we implemented relaxed molecular clocks on a species tree and reconciled the resulting chronology with gene trees of class I nitrilases to estimate their origin. Our approach has the benefit of using older calibration points spanning from 3.46 to 0.91 Ga [as oppose to 0.75 to 0.25 million years ago (Ma)] to more accurately date older Proterozoic and Archean events.

### Relative importance through time

**Fig. 2.**
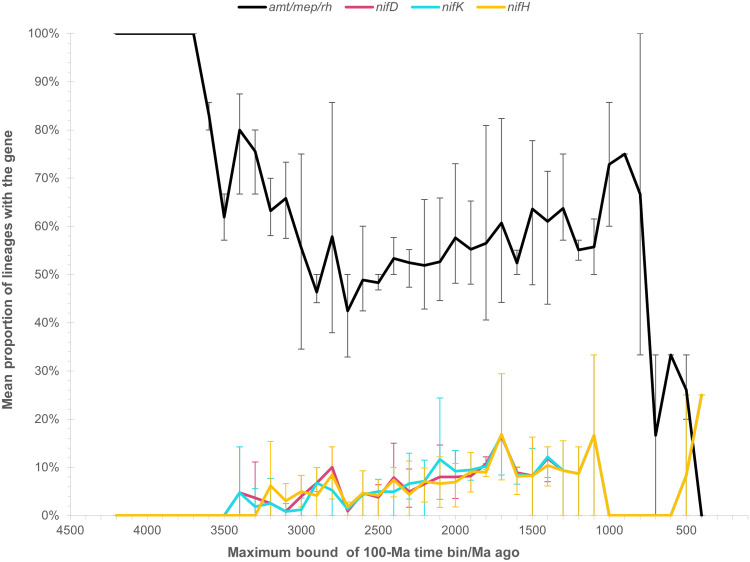
Proportion of microbial lineages with genes for ammonium uptake (black line) and biological N_2_ fixation (pink, turquoise, and yellow lines representing *nifD*, *nifK*, and *nifH*, respectively) in different time periods. Proportions represent a mean of up to three values representing the number of lineages with the gene divided by the number of lineages in the species tree in a given time bin. Each value was calculated using results from a different clock model (either CIR, UGAM, or LN). Values were not counted if the time bin contained less than three lineages. Error bars represent maximum and minimum proportions. Genes are assumed to be present in a lineage when their presence probabilities are ≥50%.

To gain insight into how important each N source was to the biosphere through time, we must have a sense of how widespread each N acquisition strategy was across the lineages that are known to have existed in different time periods. When interpreting these results, note that other lineages could have existed and subsequently gone extinct, but we do not have information about these lineages, nor do we know how they acquired N, because they did not leave traces in genomic records. Ammonium uptake genes are more widespread across the tree of life today than *nifD*, *nifK*, and *nifH* genes for N_2_ fixation, and our results estimate that this was true in all past geological eras as well (Fig. 2 and figs. S7 to S9). Since their emergence in LUCA (or LBCA), ammonium uptake genes have been present in a mean of 57 to 60% of known lineages (57% CIR, 60% UGAM, and 58% LN), whereas *nifD*, *nifK*, and *nifH* have each been restricted to less than or equal to 33% of known lineages with a mean of 6 to 9% (7 to 8% with CIR, 4 to 5% with UGAM, and 5 to 6% with LN). How abundant these lineages were remains unknown. Some modern metabolisms are restricted to few microbial groups but still impart sizeable effects on the planet (e.g., oxygenic photosynthesis is only present in one phylum of bacteria and some eukaryotes, but this relatively small subset of all lineages that exist today maintain the vast majority of oxygen in the atmosphere).

On the whole, our results indicate that most of microbial lineages can import ammonium from the environment, and these constitute a far larger number of lineages than those able to fix N_2_ or use N from urea or nitriles. We also observe that there is a drop from 100% of lineages containing *amt/mep/rh* genes for ammonium import ~4.2 Ga to ~50% of lineages with *amt/mep/rh* ~ 2.7 Ga (Fig. 2). Rather than suggesting a decrease in the number of lineages able to import ammonium, this instead mirrors an increase in the absolute number of lineages that are known to have existed, from just one at ~4.4 Ga to near 100 lineages at ~2.7 Ga (CIR) or ~1.7 Ga (UGAM and LN) (fig. S1). Therefore, our data indicate that some of the new lineages that appeared between ~3.6 and ~2.7 Ga lacked ammonium uptake genes. They must have been accessing N from other sources, such as amino acids (*6*) because genes for catabolizing nitriles appear toward the end of this window (fig. S10), and genes for catabolizing urea exhibit a similar decline (fig. S11).

Genes associated with the biological extraction of ammonium from urea and nitriles have been less widespread than ammonium uptake genes for most of the planet’s history. Class I nitrilases have existed in less than or equal to 33% of known lineages since their origin at 3.82 to 3.14 Ga, more often being present in 6 to 7% of known lineages based on means of 26 to 29 time bins, each spanning 100 Ma (ranges apply due to differing results from different clock models). The three genes for catabolizing urea have always been somewhat more widespread than class I nitrilases for accessing N from nitriles and *nif* genes for fixing N, but they have remained less widespread than *amt*/*mep*/*rh* for ammonium uptake. This is because *ureA*, *ureB*, and *ureC* have always been present in less than or equal to 57% of known lineages, with a mean of 10 to 22% since their origins in the Mesoarchean (data file S2). Therefore, our results indicate that environmental ammonium has been important for a greater diversity of microbial species than urea, nitriles, or dinitrogen gas throughout most of Earth’s history.

Although we find that genes for ammonium uptake are more common among microbes than some other N acquisition strategies, we note that our finding that 57% of extant lineages encode Amt/Mep/Rh enzymes differ from previous reports of these genes being near ubiquitous in sequenced bacterial genomes (*7*). Our search methodology correctly identifies homologs in species that have previously been reported to use ammonium transporters from the Amt/Mep/Rh family (table S7), so differences (between the near ubiquity of ammonium transporters in sequenced bacterial genomes, and their absence from 43% of the genomes analysed here) may be a result sequencing bias. Most (~90%) of sequenced genomes are from just 20 bacterial strains (*31*), with diverse samples of the type investigated here (to capture the full diversity of life) representing a small minority of public databases. As a result, our findings support the premise that *amt*/*mep*/*rh* are widespread among prokaryotes and elaborate further by suggesting that they are absent from some bacterial phyla, including Patescibacteria and WOR-3 (fig. S12).

## DISCUSSION

### Reconciling genomic and geochemical records

**Fig. 3.**
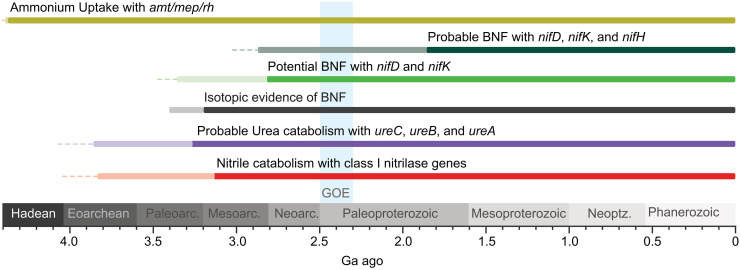
A history of microbial N sources through time. Dark colored bars indicate time periods when all three clock models agree that the metabolism existed, light colored bars represent the oldest age predicted by any of the three clock models, and dashed lines represent the oldest confidence intervals. Isotopic evidence of biological N_2_ fixation (BNF) is presented in black (see main text for references), with the gray area representing more putative isotopic evidence of biological N_2_ fixation. Paleoarc. represents the Paleoarchean, Mesoarc. represents the Mesoarchean, Neoarc. represents the Neoarchean, Neoptz. represents the Neoproterozoic.

Nitrogen is one of the most important components of biological molecules, so its relative environmental abundance acts as a key control on the size of the biosphere. Understanding the range and scope of strategies used to acquire N through Earth history can give us insights into which N sources may have been most important to the biosphere over time and thus what factors have controlled primary production over time. The results of our phylogenetic analyses suggest that microbial communities were genetically capable of importing N from environmental pools long before the appearance of any of the *nif* genes used for biological N_2_ fixation. Ammonium appears to have been the oldest N source for Earth’s earliest biosphere, but the ability to catabolize nitriles and urea also emerged early, potentially before life acquired the ability to metabolize N_2_ gas (Fig. 3). The onset of biological N_2_ fixation marks an important reference point in the evolution of the N cycle because this metabolism made the biosphere independent from planetary processes that generate bioavailable N forms. While our analyses indicate that biological N_2_ fixation with nitrogenase was not present in LUCA, there are a number of ways to interpret the data.

Most microbial species that fix N_2_ (all except thermophilic Archaea, which lack *nifN*) generate NH_4_^+^ from N_2_ using a full set of *nifD*, *nifK*, and *nifH* as well as the maturases *nifE*, *nifN*, and *nifB* (*18*). Our results estimate that the first N_2_-fixing lineage with all three catalytic subunits (namely, *nifD*, *nifK*, and *nifH*) existed at 2.88, 2.79, or 1.87 Ga (depending on the clock model), which is consistent with some other evolutionary biology studies estimating that molybdenum nitrogenases evolved at 2.11 to 1.72 Ga (*24*) or ~2.33 Ga (*21*). If *nifD* and *nifK* evolved from maturase-like ancestors, as has been previously suggested (*26*) and contested (*21*, *24*), then this first lineage with *nifD*, *nifK*, and *nifH* (the complete set of catalytic N_2_-fixing genes) may also have had *nifE* and *nifN*. However, some of these estimates, particularly the younger Paleoproterozoic estimate generated with the uncorrelated clock model, are incompatible with putative 2.1-Ga-old fossils of N_2_-fixing cyanobacteria (*32*) and the existing tenet that biological N_2_ fixation (as an oxygen-sensitive process) evolved in anoxic settings (*33*) before oxygen accumulated in the global atmosphere.

A Neoarchean to Paleoproterozoic origin of biological N_2_ fixation (between 2.88 and 1.87 Ga) would also be inconsistent with some of the earlier sedimentary N isotope records (δ^15^N = [(^15^N/^14^N)_sample_/(^15^N/^14^N)_air_ − 1] × 1000) of N_2_ fixation at 3.2 Ga. Biological N_2_ fixation with Mo-based nitrogenase (Nif) imparts very small isotopic fractionations [average of −1.4± 1.0 ‰; (*34*)], and these values have been documented from well-preserved sedimentary rocks back to 3.2 Ga and possibly 3.4 Ga (*35*–*37*). Biological N_2_ fixation may extend closer into this range because a recent evolutionary biology study estimated that all three catalytic subunits were present in an ancestral bacterium that existed ca. 3 Ga (*23*). This slightly predates our earliest estimate of 2.88 Ga due to the application of methods for interpolating between species tree nodes to get higher resolution age estimates and potentially also due to differing selection of clock models and species. However, it does not quite reach back into the older isotopic evidence for N_2_ fixation. This gap between early geochemical signatures of N_2_ fixation and the evolution of some N_2_-fixing genes has been noted previously [e.g., (*22*)].

As a result of these discrepancies, we propose that the first N_2_-fixing lineages were able to convert N_2_ into NH_4_^+^ without *nifH*. Genes encoding NifH, NifD, and NifK have been assumed to have followed the same evolutionary trajectories due to their connected roles [e.g., (*38*)], but *nifH* exists in a number of organisms that do not fix N (*25*). There might therefore be different reasons (or selective advantages) to inherit and keep *nifH*. In this study, we relaxed the assumption that *nifH*, *nifD*, and *nifK* follow the same evolutionary trajectories and found that *nifH* emerged before other nitrogenase genes in different time periods (Table 2) and lineages (Fig. 1 and Supplementary Text), similar to the results of other studies with the same assumption (*20*, *23*). Of the six genes encoding molybdenum nitrogenase, only *nifD* and *nifK*, encoding dinitrogenase/component I, interact directly with N_2_. The first lineage to host these two genes existed at 3.37, 3.21, or 2.81 Ga, depending on the clock model used (table S3). Two of these estimates are contemporaneous with early geochemical records of biological N_2_ fixation at 3.2 Ga. As these organisms would have lacked *nifH*, whose gene product donates electrons to dinitrogenase/component I to allow them to fix N_2_, an alternative reductase would have been required. Several nonbiological catalysts (including cadmium sulfide, cobaltocene, polycarboxylates, and polyaminocarboxylate ligated Eu^2+^) can reduce dinitrogenase/component I in the absence of NifH (*39*–*41*). Most limit the dinitrogenase complex to catalyzing the conversion of nitrite (NO_2_^−^), azide (N_3_^−^), or hydrazide (N_2_H_4_) to ammonium. However, cadmium sulfide can promote biological N_2_ fixation from dinitrogen gas to ammonium at 63% of the adenosine 5′-triphosphate–coupled reaction rate provided by NifH (*42*). Cadmium is rare in seawater (*43*), so the natural relevance of this mechanism remains speculative. If alternative enzymatic reductases existed in the past and were less efficient than *nifH* similar to cadmium sulfide, one could imagine that they would easily have disappeared from the genomic record when microbes with *nifD* and *nifK* acquired *nifH* in the Neoarchean to Paleoproterozoic. Therefore, further laboratory-based research into whether N_2_ fixation is possible with alternative enzymatic reductases is warranted.

**Table 2.** Estimated dates (in Ga) for nodes with ≥ 10% origination probability for N-fixing genes. CI, confidence interval.

Gene	Clock model	Origin probability	Median age/Ga	Oldest CI/Ga	Youngest CI/Ga
*nifD*	CIR	25%	3.222	3.387	3.075
		15%	3.296	3.459	3.158
		11%	3.331	3.496	3.182
	UGAM	32%	2.938	3.134	2.720
		14%	3.375	3.567	3.172
	LN	27%	3.367	3.486	3.247
		18%	3.559	3.675	3.438
		14%	3.372	3.502	3.236
*nifH*	CIR	33%	3.191	3.280	3.097
		11%	3.484	3.548	3.461
		10%	3.307	3.388	3.226
	UGAM	50%	2.938	3.134	2.720
	LN	18%	3.372	3.502	3.236
		16%	3.002	3.126	2.867
*nifK*	CIR	19%	3.296	3.459	3.158
		13%	3.209	3.374	3.071
		12%	3.222	3.387	3.075
		12%	3.331	3.496	3.182
		10%	3.053	3.215	2.899
	UGAM	19%	3.375	3.567	3.172
		12%	2.979	3.186	2.769
		11%	3.592	3.790	3.397
	LN	32%	3.367	3.486	3.247
		16%	3.175	3.310	3.034
		15%	3.193	3.330	3.051

### Abiotic sources of ammonium on early Earth

Our phylogenetic analyses reveal a gap of several hundred million years between the emergence of biological assimilation of ammonium or methylammonium from the environment and enzymatic conversion of N_2_ into ammonium within cells regardless of whether that required all three catalytic nitrogenase genes or just *nifD* and *nifK*. This long gap suggests that ammonium was freely available in the environment and able to sustain Earth’s earliest biosphere for a prolonged period of time until biological N_2_ fixation emerged. This ammonium could have been generated by a variety of abiotic sources, such as hydrothermal reduction of N_2_ by catalytic minerals within oceanic crust (*44*, *45*), continental weathering of ammonium-bearing minerals (*46*), and Fe^2+^-coupled reduction of N oxides (*8*, *9*) generated by lightning or volcanism (*4*, *47*, *48*). All of these processes have been proposed for early Earth and may also operate on other terrestrial planets. Hence, our results combined with those of others such as the tracing of glutamine synthetases and nitrite reduction enzymes back to LUCA by de Carvalho Fernandes *et al.* (*11*) and Moody *et al*. (*23*) suggest that fundamental planetary processes may be able to sustain a (small) biosphere with N over geological timescales.

Sedimentary δ^15^N data from before 3.2 Ga, when abiotic N sources were likely dominating, are generally difficult to interpret due to metamorphic and/or hydrothermal overprinting, which may have added or removed N and introduced isotopic scatter [reviewed by (*35*)]. However, it is also possible that these scattered data from the Eo- and Paleoarchean (before 3.2 Ga), which range from −4 to +11‰ (*49*), reflect at least in part microbial utilization of abiotic N sources, as suggested by our genomic results. Attempts to reconstruct primary δ^15^N values from highly metamorphosed rocks at 3.7 Ga yield negative values down to −7‰ that could be consistent with lightning or photochemical N input, for example (*4*). Alternatively, the assimilation of ammonium provided by hydrothermal processes could have played a role and contributed to these signatures (*50*).

It has been suggested that primary productivity on early Earth, before the onset of photosynthesis, was perhaps three to five orders of magnitude lower than it is today (*3*). Hence, relative to the rate of modern biological N_2_ fixation, which is on the order of 10^13^ mol N year^−1^ (*51*), abiotic source fluxes of 10^8^ to 10^10^ mol N year^−1^ would perhaps have been sufficient to maintain early life on a global scale. The required range of abiotic N input for such a small biosphere is consistent with all proposed abiotic N source fluxes, even at their lower limits (Table 1).

### Early nitriles and urea

Some models propose that nitriles (molecules with a C≡N triple bond) were critical for the origin of life because nitrile molecules can undergo reductive homologation to form precursors of important biomolecular building blocks (such as RNA, proteins, and lipids) in the presence of UV radiation (*12*). Sources of nitriles include photochemical reactions in the presence of high CH_4_ levels (*52*) and asteroid impacts (*53*). Asteroids themselves contain cyanide and nitriles, and when they hit the ground, additional nitrile compounds are generated due to the elevated pressures and temperatures (*54*). While we cannot distinguish between nitrile sources using our methods, our data provide an encouraging indication that nitriles were indeed present or at least being metabolized by early microbial life on the Mesoarchean Earth. This finding lends credence to the involvement of nitriles in prebiotic chemistry and indicates that nitriles may have continued to support life beyond its origins.

Likewise, we find early evidence of urea utilization that may also stem back to prebiotic conditions. Urea is generated in small amounts during lightning reactions (*14*), in space (*55*), via the degradation of some cyanides by UV light (*56*), and (if ammonia is present) via spontaneous formation on water droplets such as fog or sea spray (*57*). As a result, urea may have contributed to the origin of life by reacting with nitriles (specifically cyanoacetaldehyde) to create two of the five bases found in RNA and DNA (*13*). Similar to nitriles, our data suggest that urea continued to play a role in microbial metabolisms long after the establishment of the biosphere.

### What drove the emergence of N_2_-fixing genes in the Paleo- to Mesoarchean or Paleoproterozoic?

As abiotic processes were evidently capable of sustaining Earth’s biosphere for several hundred million years, what drove the emergence of N_2_-fixing genes in the Paleo- to Mesoarchean or Paleoproterozoic? There is no consensus on what selective pressures may have favored the evolution of biological N_2_ fixation (*33*), but one possible explanation is an increase in productivity, driven by the rise of photosynthesis (*22*). The rise of anoxygenic phototrophs to ecological dominance may have increased primary productivity by a factor of 10 compared to ecosystems dominated by purely chemoautotrophic metabolisms (*58*–*60*). Another increase by a factor of 10 may have been achieved by the emergence of oxygenic phototrophs (namely, Cyanobacteria) (*3*). The antiquity of anoxygenic phototrophs is not well constrained, but the origin of Cyanobacteria can be traced back to as early as 3.3 Ga (*61*–*63*). If abiotic sources of fixed N were at the lower end of proposed fluxes (Table 1), then the increased demand for bioavailable N resulting from such an increase in primary productivity caused by the evolution of Cyanobacteria would have been difficult to sustain, perhaps leading to a selective advantage for organisms that had *nif* genes. This advantage must have been substantial, given that biological N_2_ fixation is energetically costly (*33*). This interpretation does not require that early phototrophs themselves were capable of fixing N_2_ because they could have benefitted from biogenic ammonium released into the environment by other N_2_ fixers within the ecosystem.

Alternatively, it is possible that the emergence of *nif* genes was driven by a decline of abiotic N sources. Trace element data in chemical sedimentary rocks (cherts and iron formations) suggest a progressive decline in hydrothermal activity from the Eoarchean (before 3.5 Ga) into the Mesoarchean (after 3.2 Ga) (*64*). The magnitude of this decline has so far not been quantified, but it is conceivable that it curtailed the supply of hydrothermal ammonium, which was one of the largest sources of abiotically fixed N on early Earth (Table 1). It is also possible that a shift in atmospheric composition affected the rate of N oxide production during lightning (*48*). On the anoxic Archean Earth, the O atoms required for the formation of N─O bonds during lightning would have been largely derived from CO_2_, and hence, a decline in atmospheric *p*CO_2_ over time could have suppressed NO_x_ production. This would in turn have limited the supply of ammonium formed from NO*_x_* reduction via Fe^2+^ oxidation (*8*). Likewise, a decline in CH_4_ levels could have suppressed the generation of HCN by photochemistry (*52*). However, climate reconstructions of the Paleoarchean are not yet precise enough to test these hypotheses.

In summary, we reveal the first indirect evidence that abiotic processes operating on the surface of the early Earth were able to maintain a sufficient supply of bioavailable N to sustain microbial ecosystems for several hundred million years after the origin of life. These N compounds included ammonium as well as urea and nitriles, all of which have been documented in laboratory experiments and models simulating early Earth conditions (Table 1). Based on these simulations, abiotic N_2_-fixing processes have long been invoked for prebiotic scenarios that led to the formation of amino acids and nucleobases. Our results show that abiotic conversion of gaseous N_2_ into bioavailable forms sustained the biosphere long after the emergence of life and extended well into the Archean eon.

Our findings have implications for the habitability of other terrestrial planets with active volcanism and hydrological cycles at their surface because these conditions can evidently be sufficient for supplying bioavailable N—an essential nutrient for carbon-based life—through a variety of abiotic mechanisms. Together, our results suggest that abiotic sources of ammonium may be sufficient for the development of a substantial surface biosphere, with important implications for understanding the coevolution of life and planetary biospheres over time.

## MATERIALS AND METHODS

### Species tree reconstruction

#### Taxa selection

Our evolutionary tree of life included 1025 strains (data file S1) that were chosen to represent the full diversity of Bacteria and Archaea. To select representative strains, we included one genome from each order as defined by the Genome Taxonomy Database (GTDB) version 220 (*65*, *66*). Where multiple genomes were available for each order, we selected the genome with the highest completeness as estimated by CheckM2 (*67*). All genomes with less than or equal to 90% completeness or more than or equal to 5% contamination as estimated by CheckM2 (*67*) were discarded.

The resulting taxon selection was then balanced to maximize phylogenetic signal following the methods of (*68*). Briefly, this involved removing phyla with fewer than five representatives and downsampling overrepresented phyla. Overrepresented phyla are defined as those with more than “*S*” representatives, calculated using the equation below, where “*N*” represents the number of genomes present in the phylum and “*Quant*” represents the 0.9 quantile of the number of genomes in each phyla before downsampling. This quantile was calculated in R (R Core Team, 2022) using the “quantile” function

$$S=N\left[1-{\left(\frac{N-\text{Quant}}{N}\right)}^{2}\right]$$

Three overrepresented phyla were identified (namely, Pseudomonadota, Patescibacteria, and Chloroflexota) and downsampled to ensure that molecular clocks would be able to estimate an accurate age for the origin of the crown group of the phylum. To do this, the full GTDB phylogenetic tree of Bacteria was downloaded, rooted on the most recent common ancestor of a Gracilicute (namely, the Pseudomonadota with GTDB accession RS_GCF_000297215.2) and Terrabacteria (namely, the Actinobacteria with GTDB accession RS_GCF_000384115.1) based on the findings of (*69*), and labeled in TreeViewerCommandLine version 1.3.1 (*70*) to highlight genomes flagged for inclusion. Of these, random genomes were manually removed to reach the balanced number of genomes (*S*) required for that phylum. If the removal of the selected genome would have changed the MRCA of the phyla in question, that genome was kept so a valid date for the crown ancestor of each phylum could be generated in future steps.

We added several additional genomes in order to calibrate the molecular clock with microfossils and geochemical records spanning a wide range of phylogenetic groups and geological time intervals. These additional genomes included nine nuclear eukaryotic genomes, four mitochondrial genomes, four plastid genomes, six cyanobacterial genomes, two anoxychlamydial genomes [a clade within Chlamydiota that donated three subunits of hydrogenase maturase to stem eukaryotes (*71*)] and two chromatiales genomes [an order of Gammaproteobacteria which have left okenane biomarkers in the geological record (*72*)]. If any of these genomes belonged to the same order as another genome in the tree, the other genome was removed to maintain the balanced taxon selection achieved earlier (with the exception of genomes that required two or more representatives from the same order to apply the calibration).

#### Marker gene selection

We created our tree of life using an alignment of 28 protein-coding genes, selected based on their ubiquity and absence of interdomain transfers in their evolutionary history. First, we compiled a list of 79 marker genes that had been used in several previous tree-of-life studies [namely, (*68*, *73*–*77*)]. From this list of 79, we discarded any genes that had experienced interdomain horizontal gene transfer as tested and reported by (*17*). For the remaining 30 genes that had not been tested by (*17*), we reconstructed rough maximum-likelihood phylogenetic trees, using amino acid sequences. Homologs of each marker protein were identified in each of the 1025 genomes using HMMER version 3.4 (*78*) using HMM profiles downloaded from PFAM (*79*). The resulting hits were filtered using a custom python script (called filter.py), which selected the best hit for each marker protein in each genome based on the highest scoring alignment region. If that hit was truncated (defined as envelope length < 0.7) or likely to be found due to chance as opposed to homology (defined as bitscore <66 when the subject sequence was a single genome), then it was removed. These were aligned in MAFFT version 7.490 (*80*) using either the iterative E-INS-i strategy (--genafpair –maxiterate 1000) if less than 200 homolog were present or a faster progressive E-large-INS-1 strategy (--mpi –large –genafpair) if more than 200 homologs were present. Poorly sequenced residues were removed using TAPER (*81*), and uninformative columns containing gaps in ≥90% of sequences were removed using TrimAl version 1.4.rev22 (*82*) following the methodology of (*68*, *83*). Rough maximum-likelihood protein phylogenies were then generated in FastTree version 2.1.11 OpenMP (*84*) with default parameters. These trees were annotated with TreeViewerCommandLine version 1.3.1 (*70*) to distinguish between homologs from Archaeal and Bacterial genomes based on GTDB taxonomy. Any marker proteins with clades indicative of paraphyly (indicating duplication in the history of the gene encoding that protein) or substantial interdomain horizontal gene transfer were removed from our list. Rare marker genes (present in <50% of genomes) were also removed from the list, unless they were predominantly Archaeal to maintain the representation of Archaea in a dataset where Archaea represented <10% of the total genomes (largely because fewer Archaeal orders have been identified on the GTDB). Together, this left us with a set of 28 marker genes with minimal evidence of paraphyly and interdomain HGT. Twenty four of these genes were found in both Archaea and Bacteria, three were specific to Bacteria and one was specific to Archaea. The few domain-specific proteins assist with accurate phylogenetic positioning and dating within each domain, whereas the multidomain markers inform evolutionary relationships both within and between the domains.

#### Alignments for phylogenetic reconstruction

Homologs of these 28 marker proteins (selected for their ubiquity and lack of interdomain horizontal gene transfer) were used to reconstruct the phylogenetic tree. Rather than using all of the marker gene homologs that had been identified above, we carefully removed homologs that could blur the phylogenetic relationships between strains as a result of long-branch attraction (caused by abnormally high levels of mutation), gene duplication, or horizontal gene transfer. To identify homologs for removal, we reconstructed more accurate protein phylogenies for each marker gene and manually removed sequences which fell on abnormally long branches, did not group with their respective domain (unless they were from eukaryotes), or exhibited branching patterns indicative of paralogs. These accurate protein phylogenies were reconstructed in IQ-TREE version 2.2.5 (*85*) using complex mixture models and 1000 ultrafast bootstraps (*86*) with more than the default number of iterations (specified with -nm 5000) to increase the chances of ultrafast bootstrap correlation coefficients reaching convergence (at the default correlation coefficient of ≥0.99). Most protein phylogenies (19 of 28) were reconstructed with the best-fitting substitution model out of C10 combined with F for empirical state frequencies observed from the data and G4 or R4 for substitution rate heterogeneity with LG, Q.pfam (*87*), or Q.bac (*88*) (command -madd LG + G4 + C10 + F, LG + R4 + C10 + F, Q.pfam + G4 + C10 + F, Q.pfam + R4 + C10 + F, Q.bac + G4 + C10 + F, and Q.bac + R4 + C10 + F) and the standard models implemented in ModelFinder (*89*). However, because of time restraints, some genes (7 of 28) were reconstructed using the best-fitting substitution model out of the same set of mixture models and a reduced set of the standard models implemented in ModelFinder (namely, the base models LG, WAG, Q.pfam, and Q.bac). A further two genes used the same reduced set of standard models and mixture models, but without the -nm option to increase the maximum number of iterations. The alignments used as input for these phylogenies were generated as above, but this time all proteins were aligned using the accurate E-INS-I strategy (--genafpair –maxiterate 1000). In total, 64.6% of homologs of marker protein PF00009, 4.8% of homologs from PF00118, 0.5% of homologs from PF00154, and 0.2% of homologs from PF05000 were removed because they were on abnormally long branches, paralogous, or nested among homologs from a different domain.

We then removed genomes that could not be placed accurately in the species tree because they contained fewer than 15 of the resulting 28 marker proteins, unless those genomes belonged to organelles (namely, mitochondria or plastids) or symbiotic phyla [namely, Patescibacteria and DPANN including Nanoarchaeota, Nanohaloachaeota, Micrarchaeota, Iainarchaeota, Aenigmarchaeota, UAP2, and Altiarchaeota (*90*)], which are expected to have undergone genome reduction. These genomes were removed before the taxon balancing described above was completed. The final balanced genomes encode a mean of 24 marker proteins (standard deviation of 2.5). Mitochondrial genomes encode the fewest marker proteins, with each of the four mitochondrial genomes encoding one, three, five, or seven marker genes.

#### Phylogenetic reconstruction

Appropriate amino acid substitution models for each marker protein were selected using ModelFinder (*89*) with the -m MF + MERGE option to find the best partitioning scheme (*91*), the -safe option to avoid numerical underflow, −rcluster 5 to speed up the computation (a.k.a. make it complete in a matter of months instead of years) by only considering the top 5% of partitioning schemes, and an additional parameter to specify the addition of six complex mixture models (including LG + G + F + C60, LG + G + C60, Q.pfam + G + F + C60, Q.pfam + G + C60, Q.bac + G + F + C60, and Q.bac + G + C60). Two separate runs were conducted, one with the -p option to allow each partition to have its own evolutionary rate, and another with the -Q option to additionally account for heterotachy [as described in (*92*)]. The resulting partitioning scheme (*91*) chosen with the -p option had a better (closer to zero) Bayesian information criterion (BIC) score than the best partitioning scheme chosen with -Q. Therefore, an amended version of this partitioning scheme (with the original 13 partitions, but where mixture models including “C60” were modified to include “C10” instead due to computational restraints) was used to reconstruct our species tree in IQ-TREE version 2.2.5 (*85*), using the “-safe” option to prevent numerical underflow. Branch supports were estimated with 1000 replicates of ultrafast bootstrap approximations (*86*) the “-bnni” option to reduce the risk of overestimating ultrafast bootstrap branch supports and the SH-aLRT test (*93*). Three independent replicates were completed, and the tree with the best (a.k.a. closest to zero) log-likelihood value was retained for further analyses. The resulting phylogenies were visualized in TreeViewer v2.1.0 (*70*) and FigTree v.1.4.4 (*94*).

Species trees made using different methods sometimes estimate different relationships between phyla [e.g., as demonstrated here (*83*)], so we incorporated information from previous studies that thoroughly investigated macroevolutionary relationships between large clades of Bacteria and Archaea [including (*68*, *69*, *75*, *90*, *95*–*100*)] by applying a topological constraint on the relationships between Gracilicutes, Terrabacteria, Patescibacteria (formerly known as the “Candidate Phyla Radiation”), and Euryarchaeota (including Halobacteriota, Thermoplasmatota, Methanobacteriota, Hydrothermarchaeota, and Hadarchaeota) in our tree (fig. S13). This has been applied before in (*101*).

### Divergence time estimation

Our species tree was anchored to a geological timeline by implementing three relaxed Bayesian molecular clocks in Phylobayes version 4.1 (*102*). Creating a molecular clock with an alignment comprising 4510–amino acid columns and accurate evolutionary models was computationally unfeasible, so the 28 marker protein alignments were randomly subsampled using a custom code (SubsampleAlignmentRandomly.py) to retain 50% of the original alignment columns from each marker protein for molecular dating. Similar random sampling has been used in other molecular clock studies [e.g., (*96*)] because it ensures that sequence information from all strains and marker proteins are used for dating. Our resulting alignment contained 2264 amino acid positions. Topology was fixed to match the species tree, and substitution rates modeled using a uniform exchangeability matrix and an empirical profile mixture model with 20 profiles (specified using -catfix C20) (*103*). Divergence times were estimated under one of three models to reflect differing views on the inheritance of substitution rates between mother and daughter lineages, namely, lognormal (LN), CIR (*104*, *105*), and UGAM (*106*).

The first diversification in our clock, representing the root and the first radiation of LUCA into Bacteria and Archaea, was set with a uniform distribution spanning 4.4 to 3.5 Ga based on two assumptions: (i) Life could not have been present before liquid water was present on the planet’s surface (*15*, *16*), and (ii) LUCA must have emerged before the earliest generally accepted microfossils (*107*).

We also implemented 14 calibration points based on microfossils of Cyanobacteria, microfossils of algae, and geochemical evidence for methanogenesis and oxygenic photosynthesis on a scale powerful enough to cause the Great Oxygenation Event (GOE) (table S8). Given recent data suggesting that oxygenic photosynthesis evolved before crown Cyanobacteria emerged (*108*, *109*) (the term “crown” is used to refer to the most recent common ancestor of all extant Cyanobacteria), we note that the findings reported in this manuscript are based on our assumption that the crown group of the most successful modern oxygenic phototrophs in the prokaryotic domain of life (Cyanobacteria) must have been present when the atmosphere first became oxygenated at the GOE. This does not refute the possibility that other oxygenic photosynthetic lineages could have existed at this point or that more ancient ancestors in the cyanobacterial stem branch or earlier could have been photosynthetic, but it does assume that they were not efficient or widespread enough to cause oxygenation on a global scale. Further analyses may prove this wrong, but at present, our assumption that crown group Cyanobacteria emerged somewhere between the first whiffs of atmospheric oxygen at 3 Ga and the onset of the GOE at 2.32 Ga is in-line with previous molecular clock analyses dating crown group Cyanobacteria to the Neo- or Mesoarchean (*63*).

Many of these calibrations have been applied previously [e.g. (*62*, *101*, *110*–*116*)]. Here, we build on the calibrations applied in (*101*) by assuming earlier methanogenesis >3.46 Ga (*117*–*119*) and adding additional calibrations for purple sulfur bacteria, crown eukaryotes, anoxychlamdiales, metazoa, and embryophytes.

Twelve independent chains were run for 20,532 (UGAM), 97,115 (CIR), or 113,798 (LN) cycles and considered converged when two replicate chains passed all of the following criteria after discarding the first 3500 (for clocks made with CIR and LN) or 1500 (UGAM) cycles as burn-in: (i) relative differences of all parameters <0.3, (ii) effective sizes of all parameters >50, (iii) effective sample size (ESS) of all parameters >200, and (iv) Rhat of all parameters <1.05. These relative differences and effective sizes were calculated in tracecomp implemented in phylobayes v4.1 (*102*), whereas Rhat and ESS values were calculated with a custom R script (phylobayes_convergence_statistics_fixed.r). The number of chains to discard as burn-in was chosen by visual assessment of traces viewed in Tracer v.1.7.0 (*120*).

### Identification of N genes

We searched in our molecular clock genome taxa for homologs of genes involved in key pathways for the uptake of ammonium and other N compounds using HMMER3 (*78*). First, query sequences (in the form of “reviewed proteins”) were downloaded from relevant entries on InterPro 104.0 (table S9) (*121*). These were aligned with MAFFT v.7.4 (*80*) using genafpair (E-INS-i) and converted into HMM profiles using hmmbuild with default parameters. The resulting HMM profiles were used to search for homologs in the genomes of our molecular clock (all genomes were concatenated and treated as a single subject) using hmmsearch with an *e* value threshold of 0.001 (recommended in the HMMER3 user guide to minimize the chances of finding false homologs). Hits shorter than 70% the length of the shortest query sequence were removed. Ammonium transporters and nitrite nitrate symporters require transmembrane helices to transport N compounds into and out of the cell (*7*, *122*), so for these proteins only, hits that did not contain transmembrane helices [estimated by DeepTMHMM (*123*)] were removed.

A cautious approach to gene identification was taken by discarding all homologs that did not descend from the MRCA of the query sequences. Similar approaches have been used to identify phosphorus-cycling genes (*101*, *124*), compatible solute genes (*61*), light-harvesting genes (*112*), and arsenic-resistance genes (*125*) previously. Here, we identified genes that descended from the MRCA of query sequences by aligning, removing poorly sequence residues, and reconstructing rough species trees using the same methods that were used to look for evidence of interdomain HGT in the marker genes used to reconstruct the species tree. An additional step of rooting the gene trees using minimal ancestor deviation (with the -t option to retain branch lengths shorter than 10^−6^) (*126*)—one of the two best methods of rooting prokaryotic gene families (*127*)—was added to identify which branch corresponds to the common ancestor of all query sequences. Hits that passed all previous thresholds but did not descend from the MRCA of the query sequences were discarded. An additional step was added to distinguish class I nitrilases with activity on nitrile molecules (with C≡N triple bonds) from 12 other classes of nitrilases that act on different N- and carbon-containing molecules [often amides, (*29*)]. This involved removing sequences (totalling 1961 of 2051), which did not have a signature Cys-Trp-Glu amino acid motif (beginning at position 2180 in our alignment) unique to class I nitrilases (*29*), then realigning, and trimming the remaining class I nitrilases.

A different additional step was used for NifH, NifD, and NifK to ensure that closely related bacteriochlorophylls with different functions (*21*, *24*, *128*) were removed. This involved adding 138 reviewed amino acid sequences of BchX (from CD02044 and IPR010246) and BchL/ChlL (from CD02033 and IPR010246) to the alignment of NifH, 174 reviewed amino acid sequences of BchN/ChlN (from IPR005970, IPR050293, NF002768.0, and NF005867.0) to the alignment of NifD, and 117 reviewed amino acid sequences of BchB/ChlB (from IPR005969 and IPR016209) to the alignment of NifK before filtering out sequences that did not descend from the MRCA of the relevant N fixing proteins. Fortunately, all bacteriochlorophylls were outgroups of these. Maturases (NifE and NifN, otherwise known as “assembly scaffold proteins”) were not kept for further analyses because they cannot efficiently reduce dinitrogen, although laboratory studies have shown that their ancestors may have been capable of limited N_2_ fixation (*129*).

### Evolutionary trajectories of N uptake genes

Gene trees were reconstructed from amino acid alignments generated with the genafpair option (E-Ins-i) in MAFFT (*80*). Poorly sequenced residues were removed, and gappy regions were trimmed with the same criteria that have been applied throughout. For each gene, phylogenies were reconstructed in either MrBayes version 3.2.7a (*130*) (*nifD*, *nifK*, *nifH*, and class I nitrilases) or IQ-TREE version 2.2.5 (*amt/mep/rh*, *ureC*, *ureB*, and *ureA*) if MrBayes trees did not converge within 75 million generations. IQ-TREE was parameterized to use the same complex mixture models and parameters that were applied to identify and remove long branches from most of the marker genes (namely, LG + G4 + C10 + F, LG + R4 + C10 + F, Q.pfam + G4 + C10 + F, Q.pfam + R4 + C10 + F, Q.bac + G4 + C10 + F, Q.bac + R4 + C10 + F with 1000 ultrafast bootstraps), with the exception of -nm 5000, which had to be removed due to computational constraints. An additional option, -wbtl was added to output ultrafast bootstrap trees representing uncertainty in branching relationships. MrBayes was parameterized with a mixed amino acid model prior, invariant sites, and gamma-distributed site rates. Chains were considered converged when the average standard deviation of split frequencies reached <0.01, and the potential scale reduction factor between 1.00 and 1.02 after the first 25% of iterations were discarded as burn-in.

### Reconciliation of gene trees with the molecular clock

To estimate the timing for duplication, transfer, loss, and speciation events, gene phylogenies were reconciled with our time-calibrated species trees using AleRax version 1.2.1 (*131*). AleRax was parameterized to consider genes that might be absent as a result of incomplete genome sequencing (via --fraction-missing-file FractionMissing.txt) and to forbid horizontal gene transfers between lineages that did not coexist in time (via --transfer-constraint RELDATED). Uncertainty in gene tree topology was also considered by inputting a sample gene trees instead of a single topology. This sample consisted of either 1000 ultrafast bootstrap resampled trees for genes whose evolutionary histories were reconstructed in IQ-TREE or 151 to 18,991 posterior trees sampled (every 500 generations) during MrBayes runs for different genes after discarding the first 25% as burn-in. The number of posterior trees sampled is a reflection of the number of gene tree topologies that needed to be sampled before the chains reached convergence.

## Supplementary Materials

### The PDF file includes:

Supplementary Text Figs. S1 to S13 Tables S1 to S9 Legends for data files S1 and S2 References

## References

[1] L. J. Ustick, A. A. Larkin, C. A. Garcia, N. S. Garcia, M. L. Brock, J. A. Lee, N. A. Wiseman, J. K. Moore, A. C. Martiny, Metagenomic analysis reveals global-scale patterns of ocean nutrient limitation. Science 372, 287–291 (2021). 33859034 10.1126/science.abe6301

[2] C. M. Moore, M. M. Mills, K. R. Arrigo, I. Berman-Frank, L. Bopp, P. W. Boyd, E. D. Galbraith, R. J. Geider, C. Guieu, S. L. Jaccard, T. D. Jickells, J. La Roche, T. M. Lenton, N. M. Mahowald, E. Marañón, I. Marinov, J. K. Moore, T. Nakatsuka, A. Oschlies, M. A. Saito, T. F. Thingstad, A. Tsuda, O. Ulloa, Processes and patterns of oceanic nutrient limitation. Nat. Geosci. 6, 701–710 (2013).

[3] P. W. Crockford, Y. M. Bar On, L. M. Ward, R. Milo, I. Halevy, The geologic history of primary productivity. Curr. Biol. 33, 4741–4750.e5 (2023). 37827153 10.1016/j.cub.2023.09.040

[4] P. Barth, E. E. Stüeken, C. Helling, L. Rossmanith, Y. Peng, W. Walters, M. Claire, Isotopic constraints on lightning as a source of fixed nitrogen in Earth’s early biosphere. Nat. Geosci. 16, 478–484 (2023).

[5] J. M. García-Fernández, N. T. de Marsac, J. Diez, Streamlined regulation and gene loss as adaptive mechanisms in *Prochlorococcus* for optimized nitrogen utilization in oligotrophic environments. Microbiol. Mol. Biol. Rev. 68, 630–638 (2004). 15590777 10.1128/MMBR.68.4.630-638.2004 PMC539009

[6] J. Wang, D. Yan, R. Dixon, Y.-P. Wang, Deciphering the principles of bacterial nitrogen dietary preferences: A strategy for nutrient containment. MBio 7, e00792–e00716 (2016). 27435461 10.1128/mBio.00792-16 PMC4958250

[7] G. Williamson, T. Harris, A. Bizior, P. A. Hoskisson, L. Pritchard, A. Javelle, Biological ammonium transporters: Evolution and diversification. FEBS J. 291, 3786–3810 (2024). 38265636 10.1111/febs.17059

[8] D. P. Summers, S. Chang, Prebiotic ammonia from reduction of nitrite by iron (II) on the early Earth. Nature 365, 630–633 (1993). 11540245 10.1038/365630a0

[9] M. Nishizawa, T. Saito, A. Makabe, H. Ueda, M. Saitoh, T. Shibuyam, K. Takai, Stable abiotic production of ammonia from nitrate in komatiite-hosted hydrothermal systems in the Hadean and Archean oceans. Minerals 11, 321 (2021).

[10] W. C. van Heeswijk, H. V. Westerhoff, F. C. Boogerd, Nitrogen assimilation in *Escherichia coli*: Putting molecular data into a systems perspective. Microbiol. Mol. Biol. Rev. 77, 628–695 (2013). 24296575 10.1128/MMBR.00025-13 PMC3973380

[11] G. de Carvalho Fernandes, A. C. Turchetto-Zolet, L. M. P. Passaglia, Glutamine synthetase evolutionary history revisited: Tracing back beyond the last universal common ancestor. Evolution 76, 605–622 (2022). 35044684 10.1111/evo.14434

[12] B. H. Patel, C. Percivalle, D. J. Ritson, C. D. Duffy, J. D. Sutherland, Common origins of RNA, protein and lipid precursors in a cyanosulfidic protometabolism. Nat. Chem. 7, 301–307 (2015). 25803468 10.1038/nchem.2202 PMC4568310

[13] M. P. Robertson, S. L. Miller, An efficient prebiotic synthesis of cytosine and uracil. Nature 375, 772–774 (1995). 7596408 10.1038/375772a0

[14] H. J. Cleaves, J. H. Chalmers, A. Lazcano, S. L. Miller, J. L. Bada, A reassessment of prebiotic organic synthesis in neutral planetary atmospheres. Orig. Life Evol. Biosph. 38, 105–115 (2008). 18204914 10.1007/s11084-007-9120-3

[15] S. J. Mojzsis, T. M. Harrison, R. T. Pidgeon, Oxygen-isotope evidence from ancient zircons for liquid water at the Earth’s surface 4,300 Myr ago. Nature 409, 178–181 (2001). 11196638 10.1038/35051557

[16] S. A. Wilde, J. W. Valley, W. H. Peck, C. M. Graham, Evidence from detrital zircons for the existence of continental crust and oceans on the Earth 4.4 Gyr ago. Nature 409, 175–178 (2001). 11196637 10.1038/35051550

[17] E. R. R. Moody, S. Álvarez-Carretero, T. A. Mahendrarajah, J. W. Clark, H. C. Betts, N. Dombrowski, L. L. Szánthó, R. A. Boyle, S. Daines, X. Chen, N. Lane, Z. Yang, G. A. Shields, G. J. Szöllősi, A. Spang, D. Pisani, T. A. Williams, T. M. Lenton, P. C. J. Donoghue, The nature of the last universal common ancestor and its impact on the early Earth system. Nat. Ecol. Evol. 8, 1654–1666 (2024). 38997462 10.1038/s41559-024-02461-1 PMC11383801

[18] P. C. Dos Santos, Z. Fang, S. W. Mason, J. C. Setubal, R. Dixon, Distribution of nitrogen fixation and nitrogenase-like sequences amongst microbial genomes. BMC Genomics 13, 162 (2012). 22554235 10.1186/1471-2164-13-162 PMC3464626

[19] A. K. Garcia, H. McShea, B. Kolaczkowski, B. Kaçar, Reconstructing the evolutionary history of nitrogenases: Evidence for ancestral molybdenum-cofactor utilization. Geobiology 18, 394–411 (2020). 32065506 10.1111/gbi.12381 PMC7216921

[20] C. Parsons, E. E. Stüeken, C. J. Rosen, K. Mateos, R. E. Anderson, Radiation of nitrogen-metabolizing enzymes across the tree of life tracks environmental transitions in Earth history. Geobiology 19, 18–34 (2021). 33108025 10.1111/gbi.12419 PMC7894544

[21] H.-W. Pi, J.-J. Lin, C.-A. Chen, P.-H. Wang, Y.-R. Chiang, C.-C. Huang, C.-C. Young, W.-H. Li, Origin and evolution of nitrogen fixation in prokaryotes. Mol. Biol. Evol. 39, msac181 (2022). 35993177 10.1093/molbev/msac181 PMC9447857

[22] F. Mus, D. R. Colman, J. W. Peters, E. S. Boyd, Geobiological feedbacks, oxygen, and the evolution of nitrogenase. Free Radic. Biol. Med. 140, 250–259 (2019). 30735835 10.1016/j.freeradbiomed.2019.01.050

[23] E. R. R. Moody, T. A. Williams, S. Álvarez-Carretero, G. J. Szöllősi, D. Pisani, T. M. Lenton, P. C. J. Donoghue, The emergence of metabolisms through Earth history and implications for biospheric evolution. Phil. Trans. R. Soc. B 380, 20240097 (2025). 40770992 10.1098/rstb.2024.0097 PMC12329450

[24] E. S. Boyd, A. D. Anbar, S. Miller, T. L. Hamilton, M. Lavin, J. W. Peters, A late methanogen origin for molybdenum-dependent nitrogenase. Geobiology 9, 221–232 (2011). 21504537 10.1111/j.1472-4669.2011.00278.x

[25] K. Mise, Y. Masuda, K. Senoo, H. Itoh, Undervalued pseudo-*nifH* sequences in public databases distort metagenomic insights into biological nitrogen fixers. mSphere 6, e0078521 (2021). 34787447 10.1128/msphere.00785-21 PMC8597730

[26] P. S. Garcia, S. Gribaldo, G. Borrel, Diversity and evolution of methane-related pathways in archaea. Annu. Rev. Microbiol. 76, 727–755 (2022). 35759872 10.1146/annurev-micro-041020-024935

[27] K. Kappaun, A. R. Piovesan, C. R. Carlini, R. Ligabue-Braun, Ureases: Historical aspects, catalytic, and non-catalytic properties—A review. J. Adv. Res. 13, 3–17 (2018). 30094078 10.1016/j.jare.2018.05.010 PMC6077230

[28] S. L. Schwartz, L. T. Rangel, J. G. Payette, G. P. Fournier, A Proterozoic microbial origin of extant cyanide-hydrolyzing enzyme diversity. Front. Microbiol. 14, 1130310 (2023). 37065136 10.3389/fmicb.2023.1130310 PMC10098168

[29] H. C. Pace, C. Brenner, The nitrilase superfamily: Classification, structure and function. Genome Biol. 2, reviews0001.1 (2001). 11380987 10.1186/gb-2001-2-1-reviews0001 PMC150437

[30] R. Egelkamp, T. Zimmermann, D. Schneider, R. Hertel, R. Daniel, Impact of nitriles on bacterial communities. Front. Environ. Sci. 7, 103 (2019).

[31] G. A. Blackwell, M. Hunt, K. M. Malone, L. Lima, G. Horesh, B. T. F. Alako, N. R. Thomson, Z. Iqbal, Exploring bacterial diversity via a curated and searchable snapshot of archived DNA sequences. PLOS Biol. 19, e3001421 (2021). 34752446 10.1371/journal.pbio.3001421 PMC8577725

[32] A. Tomitani, A. H. Knoll, C. M. Cavanaugh, T. Ohno, The evolutionary diversification of Cyanobacteria: Molecular-phylogenetic and paleontological perspectives. Proc. Natl. Acad. Sci. U.S.A. 103, 5442–5447 (2006). 16569695 10.1073/pnas.0600999103 PMC1459374

[33] H. R. Rucker, B. Kaçar, Enigmatic evolution of microbial nitrogen fixation: Insights from Earth’s past. Trends Microbiol. 32, 554–564 (2024). 37061455 10.1016/j.tim.2023.03.011

[34] N. Wannicke, E. Stüeken Eva, T. Bauersachs, M. Gehringer, Exploring the influence of atmospheric CO_2_ and O_2_ levels on the utility of nitrogen isotopes as proxy for biological N_2_ fixation. Appl. Environ. Microbiol. 90, e0057424 (2024). 39320082 10.1128/aem.00574-24 PMC11497790

[35] E. E. Stüeken, R. Buick, B. M. Guy, M. C. Koehler, Isotopic evidence for biological nitrogen fixation by molybdenum-nitrogenase from 3.2 Gyr. Nature 520, 666–669 (2015). 25686600 10.1038/nature14180

[36] M. Homann, P. Sansjofre, M. Van Zuilen, C. Heubeck, J. Gong, B. Killingsworth, I. S. Foster, A. Airo, M. J. Van Kranendonk, M. Ader, S. V. Lalonde, Microbial life and biogeochemical cycling on land 3,220 million years ago. Nat. Geosci. 11, 665–671 (2018).

[37] A. Pellerin, C. Thomazo, M. Ader, J. Marin-Carbonne, J. Alleon, E. Vennin, A. Hofmann, Iron-mediated anaerobic ammonium oxidation recorded in the early Archean ferruginous ocean. Geobiology 21, 277–289 (2023). 36637027 10.1111/gbi.12540

[38] H.-W. Pi, Y.-R. Chiang, W.-H. Li, Mapping geological events and nitrogen fixation evolution onto the timetree of the evolution of nitrogen-fixation genes. Mol. Biol. Evol. 41, msae023 (2024). 38319744 10.1093/molbev/msae023 PMC10881105

[39] R. D. Milton, S. Abdellaoui, N. Khadka, D. R. Dean, D. Leech, L. C. Seefeldt, S. D. Minteer, Nitrogenase bioelectrocatalysis: Heterogeneous ammonia and hydrogen production by MoFe protein. Energy Environ. Sci. 9, 2550–2554 (2016).

[40] K. Danyal, A. J. Rasmussen, S. M. Keable, B. S. Inglet, S. Shaw, O. A. Zadvornyy, S. Duval, D. R. Dean, S. Raugei, J. W. Peters, L. C. Seefeldt, Fe protein-independent substrate reduction by nitrogenase MoFe protein variants. Biochemistry 54, 2456–2462 (2015). 25831270 10.1021/acs.biochem.5b00140

[41] K. Danyal, B. S. Inglet, K. A. Vincent, B. M. Barney, B. M. Hoffman, F. A. Armstrong, D. R. Dean, L. C. Seefeldt, Uncoupling nitrogenase: Catalytic reduction of hydrazine to ammonia by a MoFe protein in the absence of Fe protein-ATP. J. Am. Chem. Soc. 132, 13197–13199 (2010). 20812745 10.1021/ja1067178 PMC2944900

[42] K. A. Brown, D. F. Harris, M. B. Wilker, A. Rasmussen, N. Khadka, H. Hamby, S. Keable, G. Dukovic, J. W. Peters, L. C. Seefeldt, P. W. King, Light-driven dinitrogen reduction catalyzed by a CdS:nitrogenase MoFe protein biohybrid. Science 352, 448–450 (2016). 27102481 10.1126/science.aaf2091

[43] J. P. Riley, D. Taylor, The concentrations of cadmium, copper, iron, manganese, molybdenum, nickel, vanadium and zinc in part of the tropical north-east Atlantic ocean. Deep-Sea Res. Oceanogr. Abstr. 19, 307–317 (1972).

[44] J. A. Brandes, N. Z. Boctor, G. D. Cofy, B. A. Cooper, R. M. Hazen, H. S. Yoder, Abiotic nitrogen reduction on the early Earth. Nature 395, 365–367 (1998). 9759725 10.1038/26450

[45] A. Smirnov, D. Hausner, R. Laffers, D. R. Strongin, M. A. A. Schoonen, Abiotic ammonium formation in the presence of Ni-Fe metals and alloys and its implications for the Hadean nitrogen cycle. Geochem. Trans. 9, 5 (2008). 18489746 10.1186/1467-4866-9-5 PMC2430951

[46] E. E. Stüeken, F. S. M. Holland, S. Mikhail, Igneous rocks as a viable source of fixed nitrogen to the prebiotic world. Geochem. Perspect. Lett. 35, 13–17 (2025).

[47] T. A. Mather, D. M. Pyle, A. G. Allen, Volcanic source for fixed nitrogen in the early Earth’s atmosphere. Geology 32, 905–908 (2004).

[48] R. Navarro-González, C. P. McKay, D. N. Mvondo, A possible nitrogen crisis for Archaean life due to reduced nitrogen fixation by lightning. Nature 412, 61–64 (2001). 11452304 10.1038/35083537

[49] B. W. Johnson, E. E. Stüeken, in *Treatise on Geochemistry*, A. Anbar, D. Weis, Eds. (Elsevier, ed. 3, 2025), pp. 177–201.

[50] D. L. Pinti, K. Hashizume, J.-i. Matsuda, Nitrogen and argon signatures in 3.8 to 2.8 Ga metasediments: Clues on the chemical state of the Archean ocean and the deep biosphere. Geochim. Cosmochim. Acta 65, 2301–2315 (2001).

[51] W.-L. Wang, J. K. Moore, A. C. Martiny, F. W. Primeau, Convergent estimates of marine nitrogen fixation. Nature 566, 205–211 (2019). 30760914 10.1038/s41586-019-0911-2

[52] F. Tian, J. F. Kasting, K. Zahnle, Revisiting HCN formation in Earth’s early atmosphere. Earth Planet. Sci. Lett. 308, 417–423 (2011).

[53] Z. R. Todd, K. I. Öberg, Cometary delivery of hydrogen cyanide to the early Earth. Astrobiology 20, 1109–1120 (2020). 32749859 10.1089/ast.2019.2187

[54] M. Ferus, S. Civiš, A. Mládek, J. Šponer, L. Juha, J. E. Šponer, On the Road from Formamide Ices to Nucleobases: IR-spectroscopic observation of a direct reaction between cyano radicals and formamide in a high-energy impact event. J. Am. Chem. Soc. 134, 20788–20796 (2012). 23193998 10.1021/ja310421z

[55] F. S. Brigiano, Y. Jeanvoine, A. Largo, R. Spezia, The formation of urea in space I. Ion-molecule, neutral-neutral, and radical gas-phase reactions. Astron. Astrophys. 610, A26 (2018).

[56] D. M. Ratnayake, R. Tanaka, E. Nakamura, Biogeochemical impact of nickel and urea in the great oxidation event. Commun. Earth Environ. 6, 654 (2025).

[57] M. A. Mohajer, P. Basuri, A. Evdokimov, G. David, D. Zindel, E. Miliordos, R. Signorell, Spontaneous formation of urea from carbon dioxide and ammonia in aqueous droplets. Science 388, 1426–1430 (2025). 40570122 10.1126/science.adv2362

[58] P. Kharecha, J. Kasting, J. Siefert, A coupled atmosphere–ecosystem model of the early Archean Earth. Geobiology 3, 53–76 (2005).

[59] D. E. Canfield, M. T. Rosing, C. Bjerrum, Early anaerobic metabolisms. Phil. Trans. R. Soc. B 361, 1819–1836 (2006). 17008221 10.1098/rstb.2006.1906 PMC1664682

[60] L. M. Ward, B. Rasmussen, W. W. Fischer, Primary productivity was limited by electron donors prior to the advent of oxygenic photosynthesis. J. Geophys. Res. Biogeo. 124, 211–226 (2019).

[61] G. Bianchini, M. Hagemann, P. Sánchez-Baracaldo, Stochastic character mapping, bayesian model selection, and biosynthetic pathways shed new light on the evolution of habitat preference in Cyanobacteria. Syst. Biol. 73, 644–665 (2024). 38934241 10.1093/sysbio/syae025 PMC11505929

[62] J. S. Boden, K. O. Konhauser, L. J. Robbins, P. Sánchez-Baracaldo, Timing the evolution of antioxidant enzymes in Cyanobacteria. Nat. Commun. 12, 4742 (2021). 34362891 10.1038/s41467-021-24396-y PMC8346466

[63] G. P. Fournier, K. R. Moore, L. T. Rangel, J. G. Payette, L. Momper, T. Bosak, The Archean origin of oxygenic photosynthesis and extant cyanobacterial lineages. Proc. R. Soc. B 288, 20210675 (2021). 10.1098/rspb.2021.0675 PMC8479356 34583585

[64] S. Viehmann, E. E. Stüeken, S. V. Hohl, N. Tepe, Y. Lin, D. Kraemer, M. V. Kranendonk, J. Krayer, D. M. Ernst, S. Weyer, Europium traces the impact of high temperature hydrothermal systems on the early oceans. Geochem. Perspect. Lett. 34, 57–61 (2025).

[65] D. H. Parks, M. Chuvochina, P. A. Chaumeil, C. Rinke, A. J. Mussig, P. Hugenholtz, A complete domain-to-species taxonomy for bacteria and archaea. Nat. Biotechnol. 38, 1079–1086 (2020). 32341564 10.1038/s41587-020-0501-8

[66] D. H. Parks, M. Chuvochina, D. W. Waite, C. Rinke, A. Skarshewski, P. A. Chaumeil, P. Hugenholtz, A standardized bacterial taxonomy based on genome phylogeny substantially revises the tree of life. Nat. Biotechnol. 36, 996–1004 (2018). 30148503 10.1038/nbt.4229

[67] A. Chklovski, D. H. Parks, B. J. Woodcroft, G. W. Tyson, CheckM2: A rapid, scalable and accurate tool for assessing microbial genome quality using machine learning. Nat. Methods 20, 1203–1212 (2023). 37500759 10.1038/s41592-023-01940-w

[68] C. A. Martinez-Gutierrez, F. O. Aylward, Phylogenetic signal, congruence, and uncertainty across bacteria and archaea. Mol. Biol. Evol. 38, 5514–5527 (2021). 34436605 10.1093/molbev/msab254 PMC8662615

[69] G. A. Coleman, A. A. Davin, T. A. Mahendrarajah, L. L. Szantho, A. Spang, P. Hugenholtz, G. J. Szöllősi, T. A. Williams, A rooted phylogeny resolves early bacterial evolution. Science 372, eabea0511 (2021). 10.1126/science.abe0511 33958449

[70] G. Bianchini, P. Sánchez-Baracaldo, TreeViewer: Flexible, modular software to visualise and manipulate phylogenetic trees. Ecol. Evol. 14, e10873 (2024). 38314311 10.1002/ece3.10873 PMC10834882

[71] C. W. Stairs, J. E. Dharamshi, D. Tamarit, L. Eme, S. L. Jørgensen, A. Spang, T. J. G. Ettema, Chlamydial contribution to anaerobic metabolism during eukaryotic evolution. Sci. Adv. 6, eabb7258 (2020). 32923644 10.1126/sciadv.abb7258 PMC7449678

[72] J. J. Brocks, P. Schaeffer, Okenane, a biomarker for purple sulfur bacteria (Chromatiaceae), and other new carotenoid derivatives from the 1640Ma Barney Creek Formation. Geochim. Cosmochim. Acta 72, 1396–1414 (2008).

[73] S. Sunagawa, D. R. Mende, G. Zeller, F. Izquierdo-Carrasco, S. A. Berger, J. R. Kultima, L. Coelho, M. Arumugam, J. Tap, H. Nielsen, S. Rasmussen, S. Brunak, O. Pedersen, F. Guarner, W. de Vos, J. Wang, J. Li, J. Doré, S. Ehrlich, A. Stamatakis, P. Bork, Metagenomic species profiling using universal phylogenetic marker genes. Nat. Methods 10, 1196–1199 (2013). 24141494 10.1038/nmeth.2693

[74] L. A. Hug, B. J. Baker, K. Anantharaman, C. T. Brown, A. J. Probst, C. J. Castelle, C. N. Butterfield, A. W. Hernsdorf, Y. Amano, K. Ise, Y. Suzuki, N. Dudek, S. A. Relman, K. M. Finstad, R. Amundson, B. C. Thomas, J. F. Banfield, A new view of the tree of life. Nat. Microbiol. 1, 16048 (2016). 27572647 10.1038/nmicrobiol.2016.48

[75] H. C. Betts, M. N. Puttick, J. W. Clark, T. A. Williams, P. C. J. Donoghue, D. Pisani, Integrated genomic and fossil evidence illuminates life's early evolution and eukaryote origin. Nat. Ecol. Evol. 2, 1556–1562 (2018). 30127539 10.1038/s41559-018-0644-x PMC6152910

[76] T. A. Williams, C. J. Cox, P. G. Foster, G. J. Szöllosi, T. M. Embley, Phylogenomics provides robust support for a two-domains tree of life. Nat. Ecol. Evol. 4, 138–147 (2020). 31819234 10.1038/s41559-019-1040-x PMC6942926

[77] E. R. R. Moody, T. A. Mahendrarajah, N. Dombrowski, J. W. Clark, C. Petitjean, P. Offre, G. J. Szöllősi, A. Spang, T. A. Williams, An estimate of the deepest branches of the tree of life from ancient vertically evolving genes. eLife 11, e66695 (2022). 35190025 10.7554/eLife.66695 PMC8890751

[78] S. R. Eddy, Accelerated Profile HMM Searches. PLOS Comput. Biol. 7, e1002195 (2011). 22039361 10.1371/journal.pcbi.1002195 PMC3197634

[79] J. Mistry, S. Chuguransky, L. Williams, M. Qureshi, G. A. Salazar, E. L. L. Sonnhammer, S. C. E. Tosatto, L. Paladin, S. Raj, L. J. Richardson, R. D. Finn, A. Bateman, Pfam: The protein families database in 2021. Nucleic Acids Res. 49, D412–D419 (2021). 33125078 10.1093/nar/gkaa913 PMC7779014

[80] K. Katoh, D. M. Standley, MAFFT multiple sequence alignment software version 7: Improvements in performance and usability. Mol. Biol. Evol. 30, 772–780 (2013). 23329690 10.1093/molbev/mst010 PMC3603318

[81] C. Zhang, Y. Zhao, E. L. Braun, S. Mirarab, TAPER: Pinpointing errors in multiple sequence alignments despite varying rates of evolution. Methods Ecol. Evol. 12, 2145–2158 (2021).

[82] S. Capella-Gutierrez, J. M. Silla-Martinez, T. Gabaldon, trimAl: A tool for automated alignment trimming in large-scale phylogenetic analyses. Bioinformatics 25, 1972–1973 (2009). 19505945 10.1093/bioinformatics/btp348 PMC2712344

[83] C. A. Martinez-Gutierrez, J. C. Uyeda, F. O. Aylward, A timeline of bacterial and archaeal diversification in the ocean. eLife 12, RP88268 (2023). 38059790 10.7554/eLife.88268 PMC10703444

[84] M. N. Price, P. S. Dehal, A. P. Arkin, FastTree 2-approximately maximum-likelihood trees for large alignments. PLOS ONE 5, e9490 (2010). 20224823 10.1371/journal.pone.0009490 PMC2835736

[85] B. Q. Minh, H. A. Schmidt, O. Chernomor, D. Schrempf, M. D. Woodhams, A. von Haeseler, R. Lanfear, IQ-TREE 2: New models and efficient methods for phylogenetic inference in the genomic era. Mol. Biol. Evol. 37, 1530–1534 (2020). 32011700 10.1093/molbev/msaa015 PMC7182206

[86] D. T. Hoang, O. Chernomor, A. von Haeseler, B. Q. Minh, L. S. Vinh, UFBoot2: Improving the ultrafast bootstrap approximation. Mol. Biol. Evol. 35, 518–522 (2017). 10.1093/molbev/msx281 PMC5850222 29077904

[87] B. Q. Minh, C. C. Dang, L. S. Vinh, R. Lanfear, QMaker: Fast and accurate method to estimate empirical models of protein evolution. Syst. Biol. 70, 1046–1060 (2021). 33616668 10.1093/sysbio/syab010 PMC8357343

[88] C. C. Dang, L. S. Vinh, Estimating amino acid substitution models and rooting bacterial trees. J. Comput. Sci. Cybern. 40, 53–66 (2024).

[89] S. Kalyaanamoorthy, B. Q. Minh, T. K. F. Wong, A. von Haeseler, L. S. Jermiin, ModelFinder: Fast model selection for accurate phylogenetic estimates. Nat. Methods 14, 587–589 (2017). 28481363 10.1038/nmeth.4285 PMC5453245

[90] C. Rinke, M. Chuvochina, A. J. Mussig, P.-A. Chaumeil, A. A. Davín, D. W. Waite, W. B. Whitman, D. H. Parks, P. Hugenholtz, A standardized archaeal taxonomy for the Genome Taxonomy Database. Nat. Microbiol. 6, 946–959 (2021). 34155373 10.1038/s41564-021-00918-8

[91] O. Chernomor, A. von Haeseler, B. Q. Minh, Terrace aware data structure for phylogenomic inference from supermatrices. Syst. Biol. 65, 997–1008 (2016). 27121966 10.1093/sysbio/syw037 PMC5066062

[92] P. Lopez, D. Casane, H. Philippe, Heterotachy, an important process of protein evolution. Mol. Biol. Evol. 19, 1–7 (2002). 11752184 10.1093/oxfordjournals.molbev.a003973

[93] S. Guindon, J. F. Difayard, V. Lefort, M. Anisimova, W. Hordijk, O. Gascuel, New algorithms and methods to estimate maximum-likelihood phylogenies: Assessing the performance of PhyML 3.0. Syst. Biol. 59, 307–321 (2010). 20525638 10.1093/sysbio/syq010

[94] A. Rambaut, FigTree version 1.4.4 (Institute of Evolutionary Biology, University of Edinburgh, 2018.

[95] T. A. Williams, G. J. Szöllősi, A. Spang, P. G. Foster, S. E. Heaps, B. Boussau, T. J. G. Ettema, T. Embley, Integrative modeling of gene and genome evolution roots the archaeal tree of life. Proc. Natl. Acad. Sci. U.S.A. 114, E4602–E4611 (2017). 28533395 10.1073/pnas.1618463114 PMC5468678

[96] Q. Zhu, U. Mai, W. Pfeiffer, G. Janssen, K. R. Asnicar, J. G. Sanders, S. Belda-Ferre, G. A. Al-Ghalith, M. Kopylova, E. J. McDonald, T. Kosciolek, J. T. Morton, K. C. S. Carini, S. M. S. Malik, A. J. S. Gilbert, D. Knight, G. J. S. Knight, R. Knight, Phylogenomics of 10,575 genomes reveals evolutionary proximity between domains Bacteria and Archaea. Nat. Commun. 10, 5477 (2019). 31792218 10.1038/s41467-019-13443-4 PMC6889312

[97] Y. Wang, G. Wegener, T. A. Williams, R. Xie, J. Hou, C. Tian, Y. Zhang, F. Wang, X. Xiao, A methylotrophic origin of methanogenesis and early divergence of anaerobic multicarbon alkane metabolism. Sci. Adv. 7, eabj1453 (2021). 34215592 10.1126/sciadv.abj1453 PMC11057702

[98] J. Witwinowski, A. Sartori-Rupp, N. Taib, N. Pende, T. N. Tham, D. Poppleton, J.-M. Ghigo, C. Beloin, S. Gribaldo, An ancient divide in outer membrane tethering systems in bacteria suggests a mechanism for the diderm-to-monoderm transition. Nat. Microbiol. 7, 411–422 (2022). 35246664 10.1038/s41564-022-01066-3

[99] N. Taib, D. Megrian, J. Witwinowski, P. Adam, D. Poppleton, G. Borrel, C. Beloin, S. Gribaldo, Genome-wide analysis of the Firmicutes illuminates the diderm/monoderm transition. Nat. Ecol. Evol. 4, 1661–1672 (2020). 33077930 10.1038/s41559-020-01299-7

[100] C. J. Castelle, J. F. Banfield, Major new microbial groups expand diversity and alter our understanding of the tree of life. Cell 172, 1181–1197 (2018). 29522741 10.1016/j.cell.2018.02.016

[101] J. S. Boden, J. Zhong, R. E. Anderson, E. E. Stüeken, Timing the evolution of phosphorus-cycling enzymes through geological time using phylogenomics. Nat. Commun. 15, 3703 (2024). 38697988 10.1038/s41467-024-47914-0 PMC11066067

[102] N. Lartillot, T. Lepage, S. Blanquart, PhyloBayes 3: A Bayesian software package for phylogenetic reconstruction and molecular dating. Bioinformatics 25, 2286–2288 (2009). 19535536 10.1093/bioinformatics/btp368

[103] L. S. Quang, O. Gascuel, N. Lartillot, Empirical profile mixture models for phylogenetic reconstruction. Bioinformatics 24, 2317–2323 (2008). 18718941 10.1093/bioinformatics/btn445

[104] T. Lepage, D. Bryant, H. Philippe, N. Lartillot, A general comparison of relaxed molecular clock models. Mol. Biol. Evol. 24, 2669–2680 (2007). 17890241 10.1093/molbev/msm193

[105] J. L. Thorne, H. Kishino, I. S. Painter, Estimating the rate of evolution of the rate of molecular evolution. Mol. Biol. Evol. 15, 1647–1657 (1998). 9866200 10.1093/oxfordjournals.molbev.a025892

[106] A. J. Drummond, S. Y. W. Ho, M. J. Phillips, A. Rambaut, Relaxed phylogenetics and dating with confidence. PLOS Biol. 4, 699–710 (2006). 10.1371/journal.pbio.0040088 PMC1395354 16683862

[107] F. Westall, A. Brack, A. G. Fairén, M. D. Schulte, Setting the geological scene for the origin of life and continuing open questions about its emergence. Front. Astron. Space Sci. 9, 1095701 (2023). 38274407 10.3389/fspas.2022.1095701 PMC7615569

[108] A. Nishihara, Y. Tsukatani, C. Azai, M. K. Nobu, Illuminating the coevolution of photosynthesis and bacteria. Proc. Natl. Acad. Sci. U.S.A. 121, e2322120121 (2024). 38875151 10.1073/pnas.2322120121 PMC11194577

[109] Z. C. Zeng, L. Y. Li, H. Wang, Y. X. Tao, Z. B. Lv, F. P. Wang, Y. Z. Wang, Oxidative adaptations in prokaryotes imply the oxygenic photosynthesis before crown-group Cyanobacteria. PNAS Nexus 4, pgaf035 (2025). 39949657 10.1093/pnasnexus/pgaf035 PMC11823831

[110] K. Mateos, G. Chappell, A. Klos, B. Le, J. Boden, E. E. Stüeken, R. Anderson, The evolution and spread of sulfur cycling enzymes reflect the redox state of the early Earth. Sci. Adv. 9, eade4847 (2023). 37418533 10.1126/sciadv.ade4847 PMC10328410

[111] P. Sánchez-Baracaldo, Origin of marine planktonic Cyanobacteria. Sci. Rep. 5, 17418 (2015). 26621203 10.1038/srep17418 PMC4665016

[112] P. Sánchez-Baracaldo, G. Bianchini, A. Di Cesare, C. Callieri, N. A. M. Chrismas, Insights Into the evolution of picocyanobacteria and phycoerythrin genes (*mpeBA* and *cpeBA*). Front. Microbiol. 10, 45 (2019). 30761097 10.3389/fmicb.2019.00045 PMC6363710

[113] P. Sánchez-Baracaldo, T. Cardona, On the origin of oxygenic photosynthesis and Cyanobacteria. New Phytol. 225, 1440–1446 (2020). 31598981 10.1111/nph.16249

[114] P. Sánchez-Baracaldo, P. K. Hayes, C. E. Blank, Morphological and habitat evolution in the Cyanobacteria using a compartmentalization approach. Geobiology 3, 145–165 (2005).

[115] P. Sánchez-Baracaldo, J. A. Raven, D. Pisani, A. H. Knoll, Early photosynthetic eukaryotes inhabited low-salinity habitats. Proc. Natl. Acad. Sci. U.S.A. 114, E7737–E7745 (2017). 28808007 10.1073/pnas.1620089114 PMC5603991

[116] P. Sánchez-Baracaldo, A. Ridgwell, J. A. Raven, A Neoproterozoic transition in the marine nitrogen cycle. Curr. Biol. 24, 652–657 (2014). 24583016 10.1016/j.cub.2014.01.041

[117] J. M. Wolfe, G. P. Fournier, Horizontal gene transfer constrains the timing of methanogen evolution. Nat. Ecol. Evol. 2, 897–903 (2018). 29610466 10.1038/s41559-018-0513-7

[118] B. Cavalazzi, L. Lemelle, A. Simionovici, S. L. Cady, M. J. Russell, E. Bailo, R. Canteri, E. Enrico, A. Manceau, A. Maris, M. Salome, E. Thomassot, N. Bouden, R. Tucoulou, A. Hofmann, Cellular remains in a ~3.42-billion-year-old subseafloor hydrothermal environment. Sci. Adv. 7, eabf3963 (2021). 34261651 10.1126/sciadv.abf3963 PMC8279515

[119] Y. Ueno, K. Yamada, N. Yoshida, S. Maruyama, Y. Isozaki, Evidence from fluid inclusions for microbial methanogenesis in the early Archaean era. Nature 440, 516–519 (2006). 16554816 10.1038/nature04584

[120] A. Rambaut, A. J. Drummond, D. Xie, G. Baele, M. A. Suchard, Posterior summarization in bayesian phylogenetics using Tracer 1.7. Syst. Biol. 67, 901–904 (2018). 29718447 10.1093/sysbio/syy032 PMC6101584

[121] M. Blum, A. Andreeva, L. C. Florentino, S. R. Chuguransky, T. Grego, E. Hobbs, B. L. Pinto, A. Orr, T. Paysan-Lafosse, I. Ponamareva, G. A. Salazar, N. Bordin, P. Bork, A. Bridge, L. Colwell, J. Gough, D. H. Haft, I. Letunic, F. Llinares-López, A. Marchler-Bauer, L. Meng-Papaxanthos, H. Mi, D. A. Natale, C. A. Orengo, A. P. Pandurangan, D. Piovesan, C. Rivoire, C. J. A. Sigrist, N. Thanki, F. Thibaud-Nissen, P. D. Thomas, S. C. E. Tosatto, C. H. Wu, A. Bateman, InterPro: The protein sequence classification resource in 2025. Nucleic Acids Res. 53, D444–D456 (2025). 39565202 10.1093/nar/gkae1082 PMC11701551

[122] M. Fukuda, H. Takeda, H. E. Kato, S. Doki, K. Ito, A. D. Maturana, R. Ishitani, O. Nureki, Structural basis for dynamic mechanism of nitrate/nitrite antiport by NarK. Nat. Commun. 6, 7097 (2015). 25959928 10.1038/ncomms8097 PMC4432589

[123] J. Hallgren, K. D. Tsirigos, M. D. Pedersen, J. J. A. Armenteros, P. Marcatili, H. Nielsen, A. Krogh, O. Winther, DeepTMHMM predicts alpha and beta transmembrane proteins using deep neural networks. bioRxiv 487609 [Preprint] (2022).

[124] J. S. Boden, S. M. Som, W. J. Brazelton, R. E. Anderson, E. E. Stüeken, Evaluating serpentinization as a source of phosphite to microbial communities in hydrothermal vents. Geobiology 23, e70016 (2025). 40129261 10.1111/gbi.70016 PMC11933879

[125] S.-C. Chen, G.-X. Sun, Y. Yan, K. T. Konstantinidis, S.-Y. Zhang, Y. Deng, X.-M. Li, H.-L. Cui, F. Musat, D. Popp, B. P. Rosen, Y.-G. Zhu, The Great Oxidation Event expanded the genetic repertoire of arsenic metabolism and cycling. Proc. Natl. Acad. Sci. U.S.A. 117, 10414–10421 (2020). 32350143 10.1073/pnas.2001063117 PMC7229686

[126] F. D. K. Tria, G. Landan, T. Dagan, Phylogenetic rooting using minimal ancestor deviation. Nat. Ecol. Evol. 1, 0193 (2017). 10.1038/s41559-017-0193 29388565

[127] T. Wade, L. T. Rangel, S. Kundu, G. P. Fournier, M. S. Bansal, Assessing the accuracy of phylogenetic rooting methods on prokaryotic gene families. PLOS ONE 15, e0232950 (2020). 32413061 10.1371/journal.pone.0232950 PMC7228096

[128] A. K. Garcia, B. Kolaczkowski, B. Kaçar, Reconstruction of nitrogenase predecessors suggests origin from maturase-like proteins. Genome Biol. Evol. 14, evac031 (2022). 35179578 10.1093/gbe/evac031 PMC8890362

[129] C. C. Lee, K. Górecki, M. Stang, M. W. Ribbe, Y. Hu, Cofactor maturase NifEN: A prototype ancient nitrogenase? Sci. Adv. 10, eado6169 (2024). 38865457 10.1126/sciadv.ado6169 PMC11168457

[130] F. Ronquist, M. Teslenko, P. van der Mark, D. L. Ayres, A. Darling, S. Hohna, B. Larget, L. Liu, M. A. Suchard, J. P. Huelsenbeck, MrBayes 3.2: Efficient bayesian phylogenetic inference and model choice across a large model space. Syst. Biol. 61, 539–542 (2012). 22357727 10.1093/sysbio/sys029 PMC3329765

[131] B. Morel, T. A. Williams, A. Stamatakis, G. J. Szöllosi, AleRax: A tool for gene and species tree co-estimation and reconciliation under a probabilistic model of gene duplication, transfer, and loss. Bioinformatics 40, btae162 (2024). 38514421 10.1093/bioinformatics/btae162 PMC10990685

[132] R. Navarro-Gonzalez, M. J. Molina, L. T. Molina, Nitrogen fixation by volcanic lightning in the early Earth. Geophys. Res. Lett. 25, 3123–3126 (1998).

[133] J. F. Kasting, J. C. G. Walker, Limits on oxygen concentration in the prebiological atmosphere and the rate of abiotic fixation of nitrogen. J. Geophys. Res. Oceans 86, 1147–1158 (1981).

[134] J. F. Kasting, Bolide impacts and the oxidation-state of carbon in the Earth’s early atmosphere. Orig. Life Evol. Biosph. 20, 199–231 (1990). 10.1007/BF01808105 11537523

[135] H. Nakazawa, T. Sekine, T. Kakegawa, S. Nakazawa, High yield shock synthesis of ammonia from iron, water and nitrogen available on the early Earth. Earth Planet. Sci. Lett. 235, 356–360 (2005).

[136] N. F. Wogan, D. C. Catling, K. J. Zahnle, R. Lupu, Origin-of-life molecules in the atmosphere after big impacts on the early Earth. Planet. Sci. J. 4, 169 (2023).

[137] D. E. Canfield, A. N. Glazer, P. G. Falkowski, The evolution and future of Earth’s nitrogen cycle. Science 330, 192–196 (2010). 20929768 10.1126/science.1186120

[138] E. K. Moore, B. I. Jelen, D. Giovannelli, H. Raanan, P. G. Falkowski, Metal availability and the expanding network of microbial metabolisms in the Archaean eon. Nat. Geosci. 10, 629–636 (2017).

[139] A. Bekker, H. D. Holland, P. L. Wang, D. Rumble, H. J. Stein, J. L. Hannah, L. L. Coetzee, N. J. Beukes, Dating the rise of atmospheric oxygen. Geochim. Cosmochim. Acta 68, A780–A780 (2004). 10.1038/nature02260 14712267

[140] T. Bosak, B. Liang, M. S. Sim, A. P. Petroff, Morphological record of oxygenic photosynthesis in conical stromatolites. Proc. Natl. Acad. Sci. U.S.A. 106, 10939–10943 (2009). 19564621 10.1073/pnas.0900885106 PMC2708726

[141] K. Pang, Q. Tang, J. D. Schiffbauer, J. Yao, X. Yuan, B. Wan, L. Chen, Z. Ou, S. Xiao, The nature and origin of nucleus-like intracellular inclusions in Paleoproterozoic eukaryote microfossils. Geobiology 11, 499–510 (2013). 24033870 10.1111/gbi.12053

[142] S. Golubic, V. N. Sergeev, A. H. Knoll, Mesoproterozoic *Archaeoellipsoides*: Akinetes of heterocystous cyanobacteria. Lethaia 28, 285–298 (1995). 11539549 10.1111/j.1502-3931.1995.tb01817.x

[143] T. M. Gibson, P. M. Shih, V. M. Cumming, W. W. Fischer, P. W. Crockford, M. S. W. Hodgskiss, S. Worndle, R. A. Creaser, R. H. Rainbird, T. M. Skulski, G. P. Halverson, Precise age of *Bangiomorpha pubescens* dates the origin of eukaryotic photosynthesis. Geology 46, 135–138 (2018).

[144] J. A. Zumberge, G. D. Love, P. Cárdenas, E. A. Sperling, S. Gunasekera, M. Rohrssen, E. Grosjean, J. P. Grotzinger, R. E. Summons, Demosponge steroid biomarker 26-methylstigmastane provides evidence for Neoproterozoic animals. Nat. Ecol. Evol. 2, 1709–1714 (2018). 30323207 10.1038/s41559-018-0676-2 PMC6589438

[145] J. L. Morris, M. N. Puttick, J. W. Clark, D. Edwards, P. Kenrick, S. Pressel, C. H. Wellman, Z. Yang, H. Schneider, P. C. J. Donoghue, The timescale of early land plant evolution. Proc. Natl. Acad. Sci. U.S.A. 115, E2274–E2283 (2018). 29463716 10.1073/pnas.1719588115 PMC5877938

[146] F. M. Cornejo-Castillo, A. M. Cabello, G. Szalasar, P. Sánchez-baracaldo, G. Lima-Mendez, P. Hingamp, A. Alberti, S. Sunagawa, P. Bork, C. de Vargas, J. Raes, C. Bowler, P. Wincker, J. P. Zehr, J. M. Gasol, R. Massana, S. G. Acinas, Cyanobacterial symbionts diverged in the late Cretaceous towards lineage-specific nitrogen fixation factories in single-celled phytoplankton. Nat. Commun. 7, 11071 (2016). 27002549 10.1038/ncomms11071 PMC4804200

[147] A. P. Sims, D. G. Mann, L. K. Medlin, Evolution of the diatoms: Insights from fossil, biological and molecular data. Phycologia 45, 361–402 (2006).

[148] A. Caputo, M. Stenegren, M. C. Pernice, R. A. Foster, A short comparison of two marine planktonic diazotrophic symbioses highlights an un-quantified disparity. Front. Mar. Sci. 5, 10.3389/fmicb.2019.00045 (2018).

[149] R. P. Anderson, C. R. Woltz, N. J. Tosca, S. M. Porter, D. E. G. Briggs, Fossilisation processes and our reading of animal antiquity. Trends Ecol. Evol. 38, 1060–1071 (2023). 37385847 10.1016/j.tree.2023.05.014

