# Supplementary material for: Abiotic sources of fixed nitrogen sustained early ecosystems for several hundred million years after the origin of life
Source: Sci Adv. 2026 Jun 17;12(25):eaec4450. doi: 10.1126/sciadv.aec4450 (PMC13274591; doi:10.1126/sciadv.aec4450)
Supplement: Supplementary file 1 — Supplementary Text Figs. S1 to S13 Tables S1 to S9 Legends for data files S1 and S2 References [file sciadv.aec4450_sm.pdf]

Science Advances
AAAS

Supplementary Materials for

Abiotic sources of fixed nitrogen sustained early ecosystems for several hundred million years after the origin of life

Joanne S. Boden *et al.*

Corresponding author: Joanne S. Boden, j.boden@bristol.ac.uk; Eva E. Stüeken, ees4@st-andrews.ac.uk

Sci. Adv. **12**, eaec4450 (2026)
DOI: 10.1126/sciadv.aec4450

The PDF file includes:

Supplementary Text
Figs. S1 to S13
Tables S1 to S9
Legends for data files S1 and S2
References

Other Supplementary Material for this manuscript includes the following:

Data files S1 and S2

Supplementary Text

In which ancestral micro-organisms did nitrogen-fixation emerge?

In which ancestral species biological nitrogen fixation emerged has been debated. BNF (biological nitrogen fixation) has long been considered to have emerged in archaea (specifically anaerobic methanogens) based on the basal placement of archaeal *nif* gene sequences in phylogenies (e.g. [33, 35]). But more recent phylogenies made with additional sequence data have challenged this idea by suggesting that *nif* genes emerged in bacteria (specifically in anaerobic firmicutes) [32]. Our results are in-line with the latter as the first lineage with a more than 50 % presence probability of hosting genes encoding both nitrogenase reductase (namely *nifH*) and dinitrogenase (namely *nifD* and *nifK*) is estimated to be a bacterium. This bacterium could either have been a population of Bacillota (formerly Firmicutes) as has been previously proposed [32], or a population of Desulfobacteria. Although these bacteria are likely to have been the first to fix nitrogen using all three catalytic molybdenum nitrogen-fixing genes, archaea are more likely to have hosted the first gene encoding nitrogenase reductase/component II (*nifH)* and bacteria (specifically the MRCA of Nitrospirota and Nitrospinota or Pseudomonadota) are more likely to have hosted the first genes encoding dinitrogenase/component I (namely *nifD* and *nifK*). This raises the possibility that nitrogenase subunits could have arisen in different lineages.

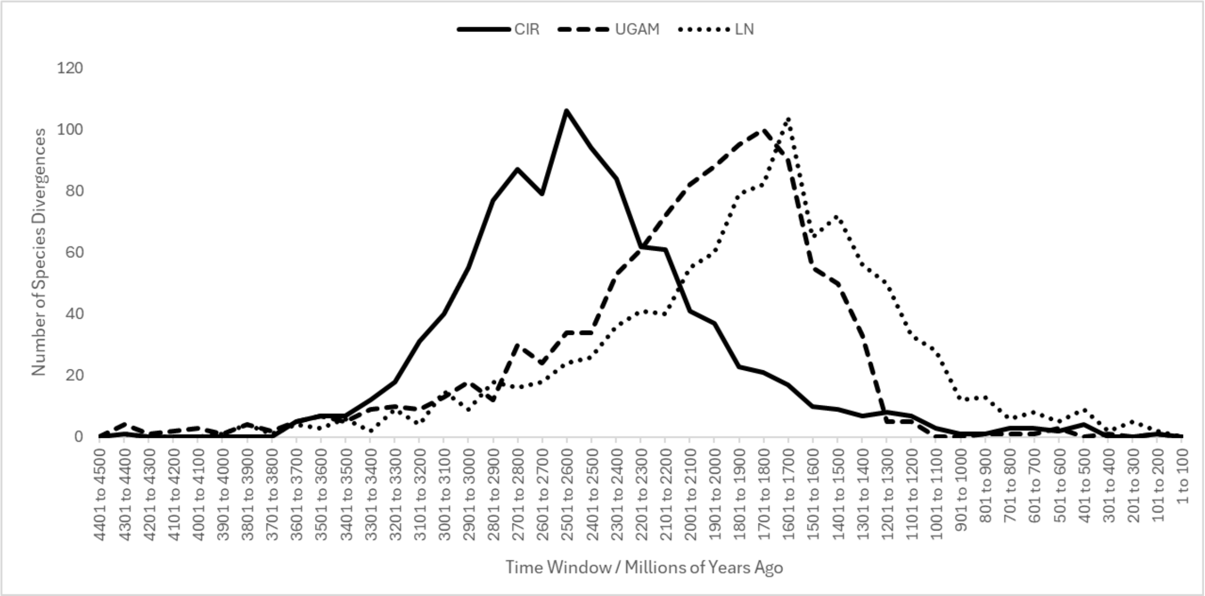

Figure S1: Number of speciations estimated by different clock models in different time periods. Clock models are CIR (solid line), UGAM (dashed line), and LN (dotted line).

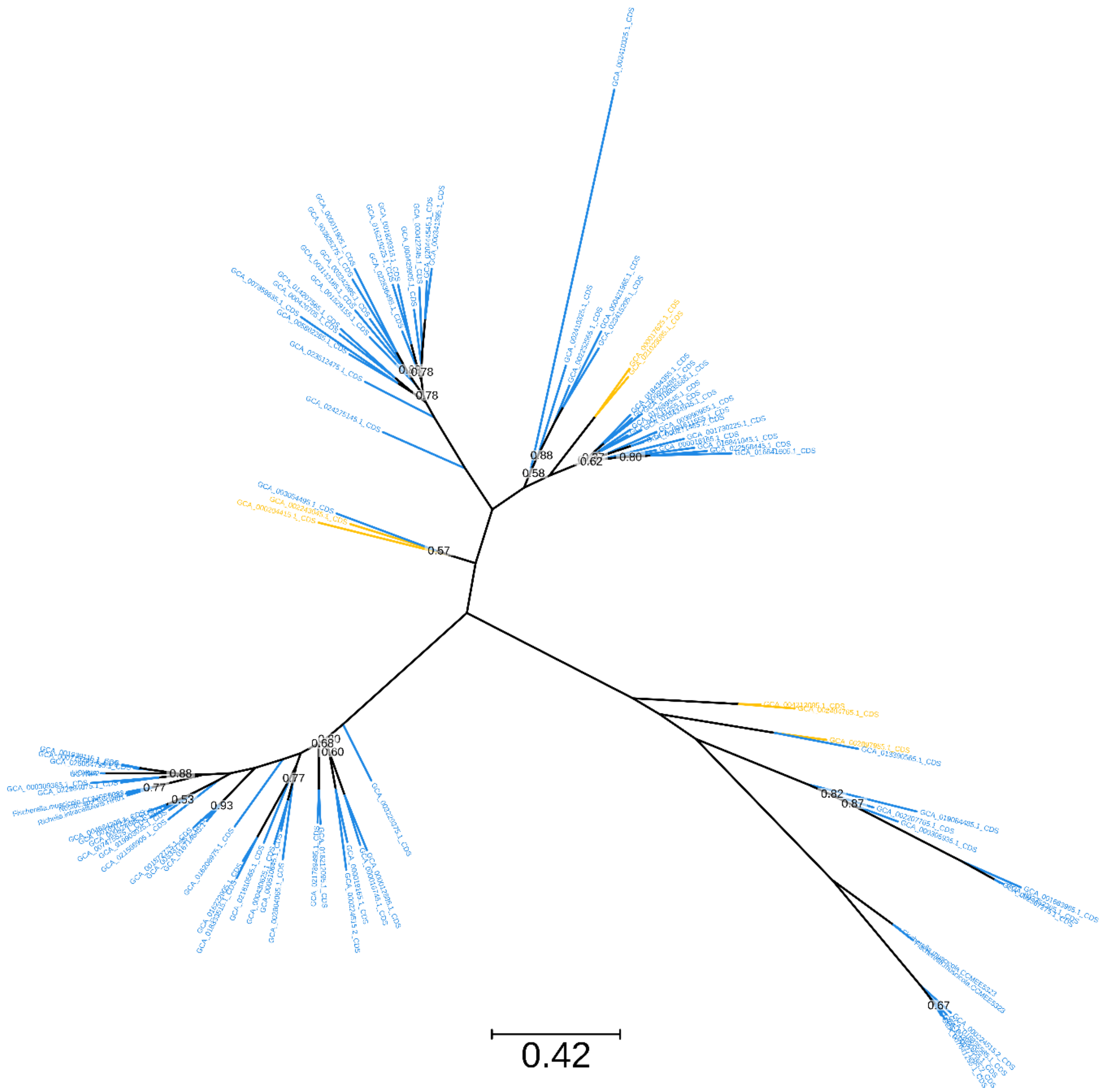

Figure S2: Bayesian NifD gene tree with branches and leaf labels coloured depending on whether the sequence is from an Archaea (yellow) or Bacteria (blue). Burn-in was discarded and the consensus tree calculated in TreeViewer. Posterior probabilities lower than 95 are indicated in black text next to the node in question. Scale bar represents the average number of amino acid substitutions per site. Leaf labels represent the GTDB ID of the genome hosting a homolog of the gene.

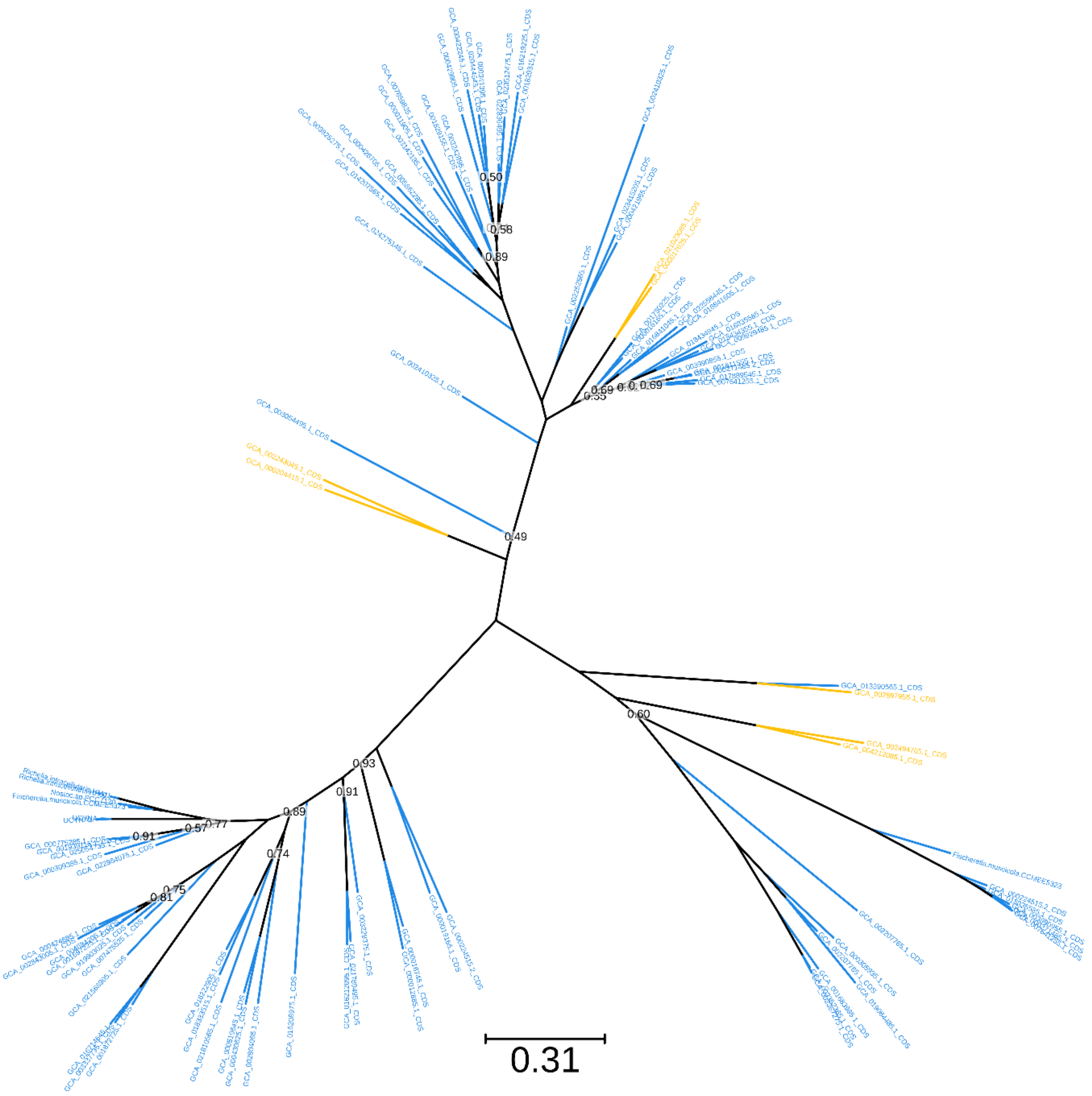

Figure S3: Bayesian NifK gene tree with branches and leaf labels coloured depending on whether the sequence is from an Archaea (yellow) or Bacteria (blue). Burn-in was discarded and the consensus tree calculated in TreeViewer. Posterior probabilities lower than 95 are indicated in black text next to the node in question. Scale bar represents the average number of amino acid substitutions per site. Leaf labels represent the GTDB ID of the genome hosting a homolog of the gene.

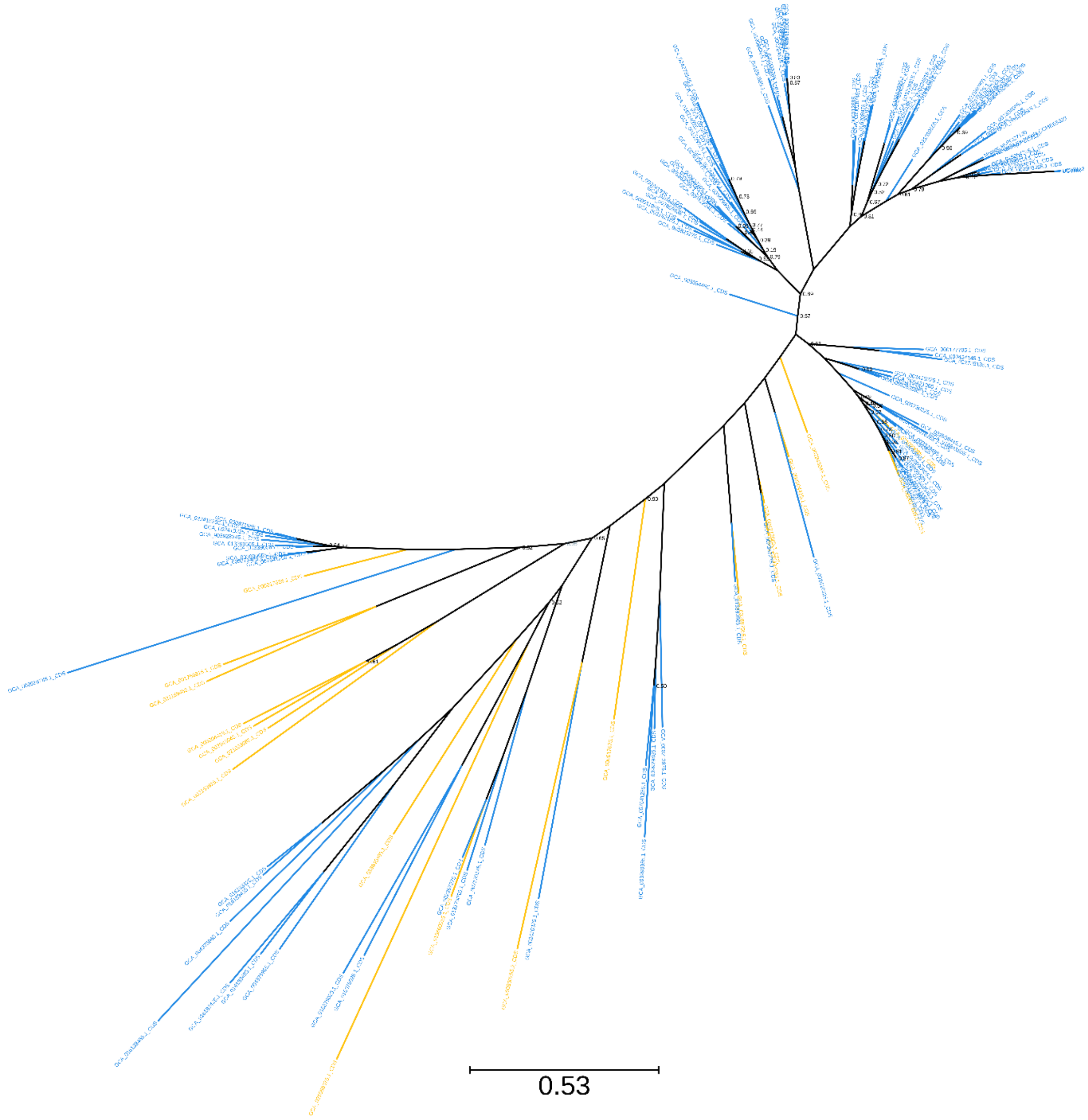

Figure S4: Bayesian NifH gene tree with branches and leaf labels coloured depending on whether the sequence is from an Archaea (yellow) or Bacteria (blue). Burn-in was discarded and the consensus tree calculated in TreeViewer. Posterior probabilities lower than 95 are indicated in black text next to the node in question. Scale bar represents the average number of amino acid substitutions per site. Leaf labels represent the GTDB ID of the genome hosting a homolog of the gene.

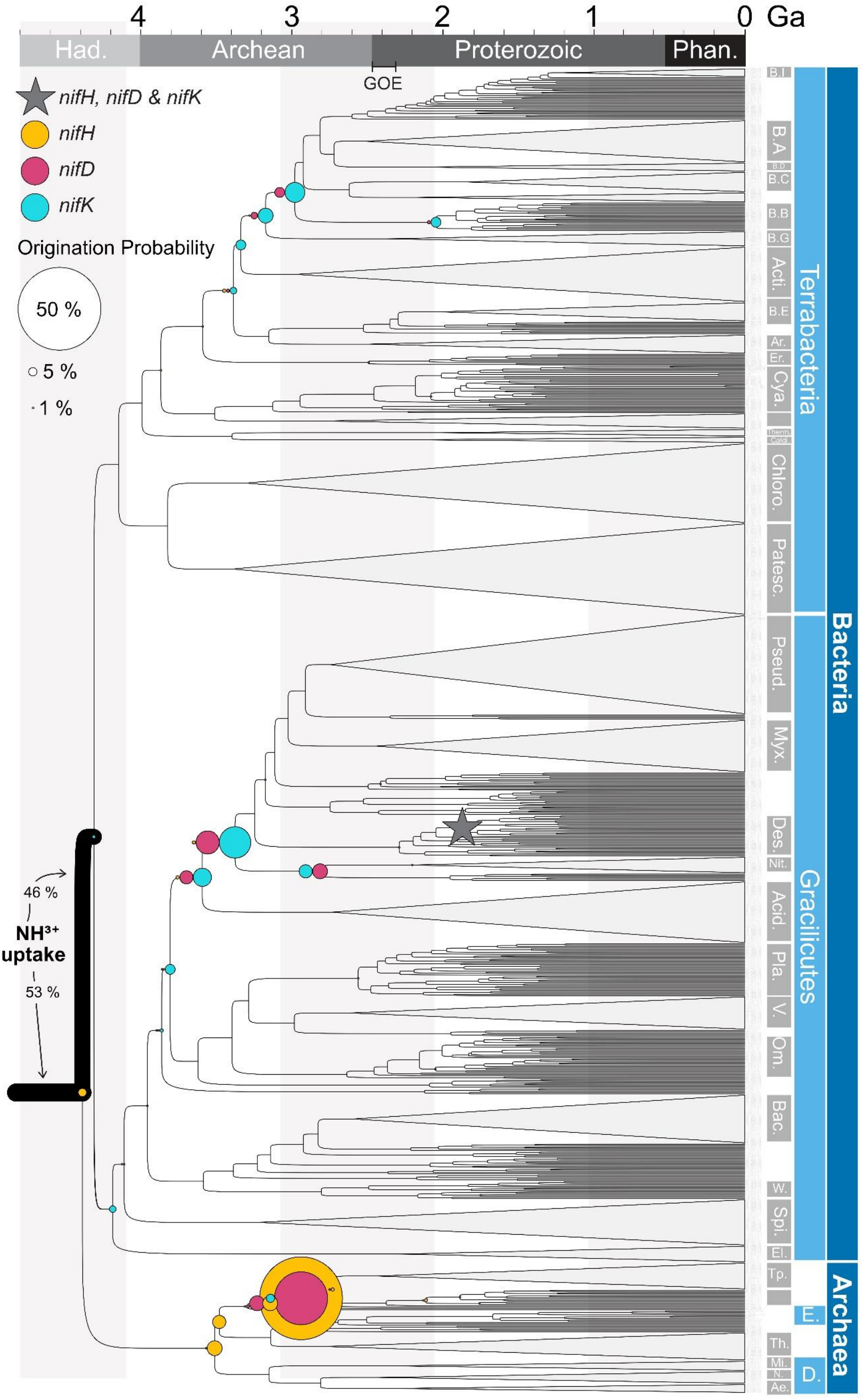

4
3
2
1
0 Ga
Had.
Archean
Proterozoic
Phan.
GOE
nifH, nifD & nifK
nifH
nifD
nifK
Origination Probability
50 %
5 %
1 %
46 %
NH3+ uptake
53 %
Terrabacteria
Gracilicutes
Bacteria
Archaea
Acti.
Cya.
Chloro.
Patesc.
Pseud.
Myx.
Des.
Nit.
Acid.
Pla.
V.
Om.
Bac.
W.
Spi.
El.
Tp.
E.
Th.
Mi.
N.
Ae.
D.

Figure S5: Ammonium-uptake enzymes stem back to LUCA or LBCA, whereas nitrogen-fixing enzymes evolved in more recent ancestral lineages of bacteria and archaea with the UGAM clock model. The most likely origins of *nifH* (yellow circles), *nifD* (pink circles) and *nifK* (turquoise circles) estimated with the UGAM clock model are annotated with coloured circles (sized in proportion to origination probability) on a time-calibrated tree of life where branch thicknesses are scaled to represent origination probabilities of genes encoding ammonium-uptake genes (namely *amt / mep / rh*). The grey star indicates the earliest lineage to host all three catalytic nitrogen fixing genes (namely *nifH, nifD* and *nifK*, 'host' is defined as > 50 % presence probability for each gene). GOE refers to the Great Oxygenation Event; Had., to the Hadean, and Phan. to the Phanerozoic. The two major domains of life are annotated with dark blue rectangles, alongside major bacterial and archaeal clades (E. refers to Euryarchaeota, and D. to DPANN), and phyla (from top to bottom B.I refers to Bacillota I, B.A refers to Bacillota A, B.D to Bacillota D, B.C to Bacillota C., B.B to Bacillota B, B.G to Bacillota G, Acti. to Actinobacteriota, B.E to Bacillota E, Ar. Armatimonadota, Er. to Eremiobacterota, Cya. to Cyanobacteriota, Ma. to Margulisbacteriota, Therm. to Thermotogota, Caldi. to Caldisericota, Chloro. to Chloroflexota., Patesc. to Patescibacteriota, Pseud. to Pseudomonadota, Myx. to Myxococcota, Des. Desulfobacterota, Nit. to Nitrospirota, Acid. to Acidobacteriota, Pla. to Planctomycetota, V. to Verrucomicrobiota, Om. to Omnitrophota, Bac. to Bacteroidota, W. to WOR-3, Spi. to Spirochaetota, El. to Elusimicrobiota, Tp. to Thermoplasmatota, Ha. to Halobacteriota, Th. to Thermoproteota, Mi. Microarchaeota, N. to Nanoarchaeota, Ae. Aenigmatarchaeota)

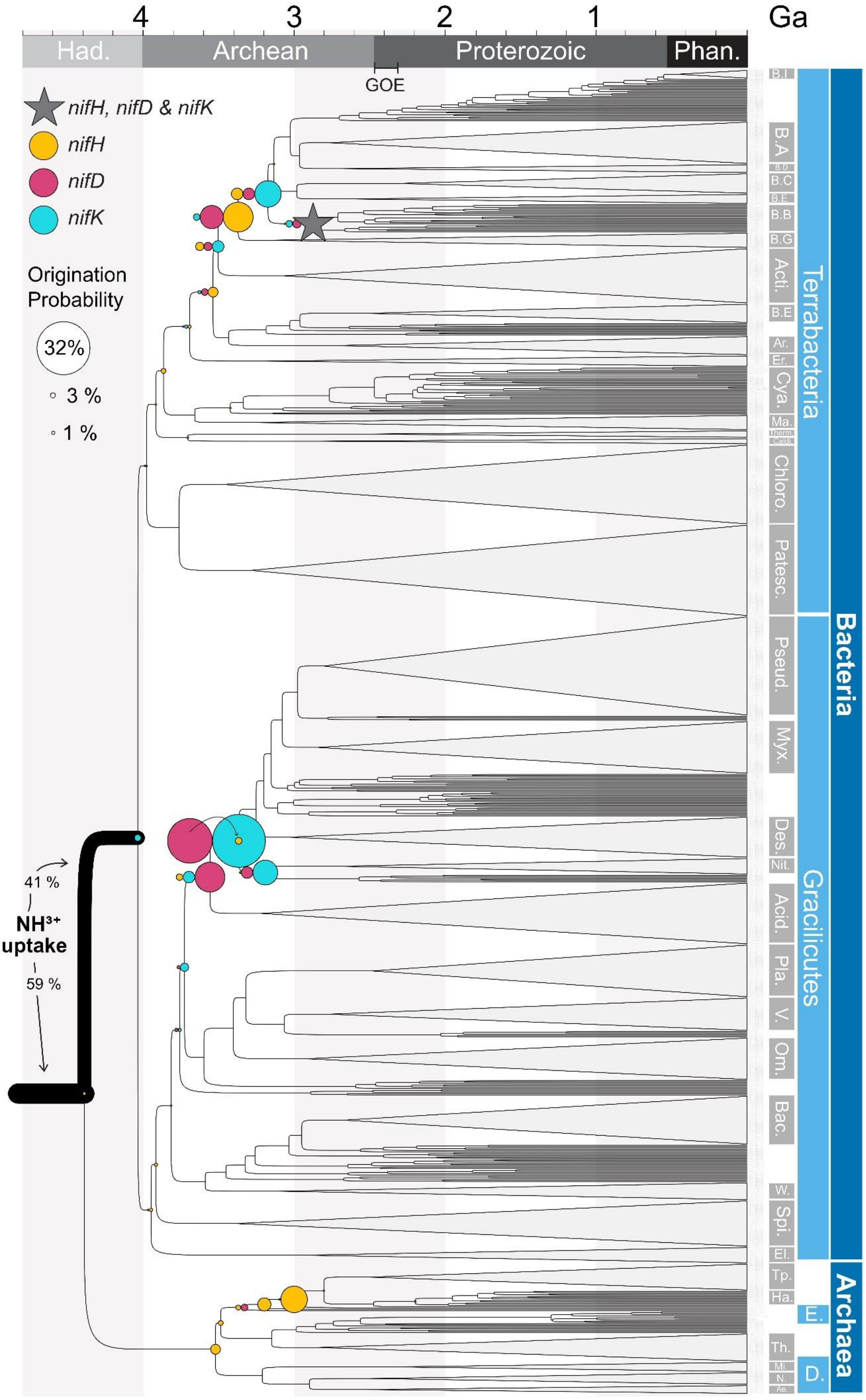

4
3
2
1
Ga
Had.
Archean
Proterozoic
Phan.
GOE
nifH, nifD & nifK
nifH
nifD
nifK
Origination Probability
32%
3 %
1 %
41 %
NH³⁺ uptake
59 %
B.A
B.C
B.B
B.G
Acti.
B.E
Ar.
Er.
Cya.
Ma.
Chloro.
Patesc.
Pseud.
Myx.
Des.
Nit.
Acid.
Pla.
V.
Om.
Bac.
W.
Spi.
El.
Tp.
Ha.
E.
Th.
D.
Terrabacteria
Gracilicutes
Bacteria
Archaea

Figure S6: Ammonium-uptake enzymes stem back to LUCA or LBCA, whereas nitrogen-fixing enzymes evolved in more recent ancestral lineages of bacteria and archaea with the LN clock model. The most likely origins of *nifH* (yellow circles), *nifD* (pink circles) and *nifK* (turquoise circles) estimated with the LN clock model are annotated with coloured circles (sized in proportion to origination probability) on a time-calibrated tree of life where branch thicknesses are scaled to represent origination probabilities of genes encoding ammonium-uptake genes (namely *amt / mep / rh*). The grey star indicates the earliest lineage to host all three catalytic nitrogen fixing genes (namely *nifH, nifD* and *nifK*, 'host' is defined as > 50 % presence probability for each gene). GOE refers to the Great Oxygenation Event; Had., to the Hadean, and Phan. to the Phanerozoic. The two major domains of life are annotated with dark blue rectangles, alongside major bacterial and archaeal clades (E. refers to Euryarchaeota, and D. to DPANN), and phyla (from top to bottom B.I refers to Bacillota I, B.A refers to Bacillota A, B.D to Bacillota D, B.C to Bacillota C., B.B to Bacillota B, B.G to Bacillota G, Acti. to Actinobacteriota, B.E to Bacillota E, Ar. Armatimonadota, Er. to Eremiobacterota, Cya. to Cyanobacteriota, Ma. to Margulisbacteriota, Therm. to Thermotogota, Caldi. to Caldisericota, Chloro. to Chloroflexota., Patesc. to Patescibacteriota, Pseud. to Pseudomonadota, Myx. to Myxococcota, Des. Desulfobacterota, Nit. to Nitrospirota, Acid. to Acidobacteriota, Pla. to Planctomycetota, V. to Verrucomicrobiota, Om. to Omnitrophota, Bac. to Bacteroidota, W. to WOR-3, Spi. to Spirochaetota, El. to Elusimicrobiota, Tp. to Thermoplasmatota, Ha. to Halobacteriota, Th. to Thermoproteota, Mi. Microarchaeota, N. to Nanoarchaeota, Ae. Aenigmatarchaeota)

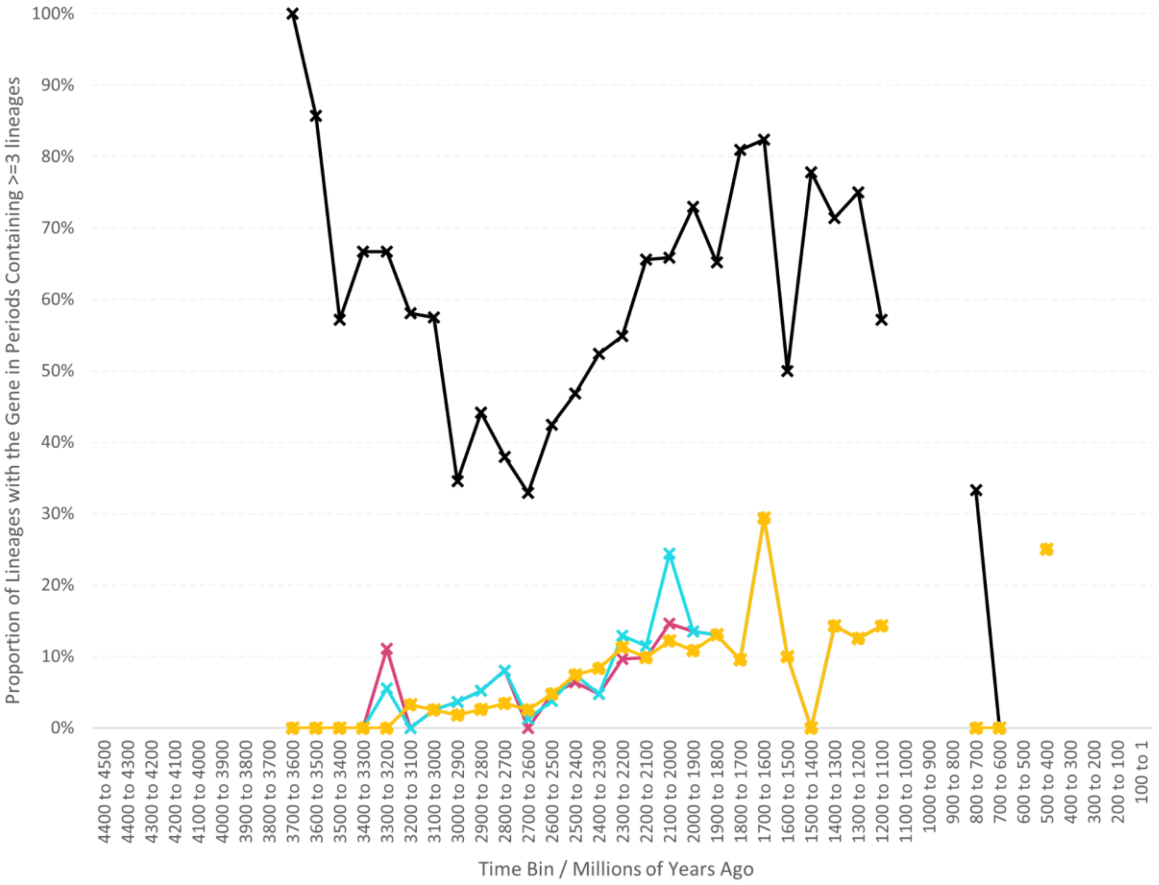

Figure S7: Proportion of lineages estimated to have genes encoding ammonium importers (black line), and three genes for biological nitrogen fixation (coloured lines) with the CIR clock model. Genes for nitrogen fixation include *nifD* (pink), *nifK* (cyan) and *nifH* (yellow). Genes are assumed to be present in a lineage when their presence probabilities are estimated to be > 50 %. Proportions are not shown for time bins containing less than 3 lineages.

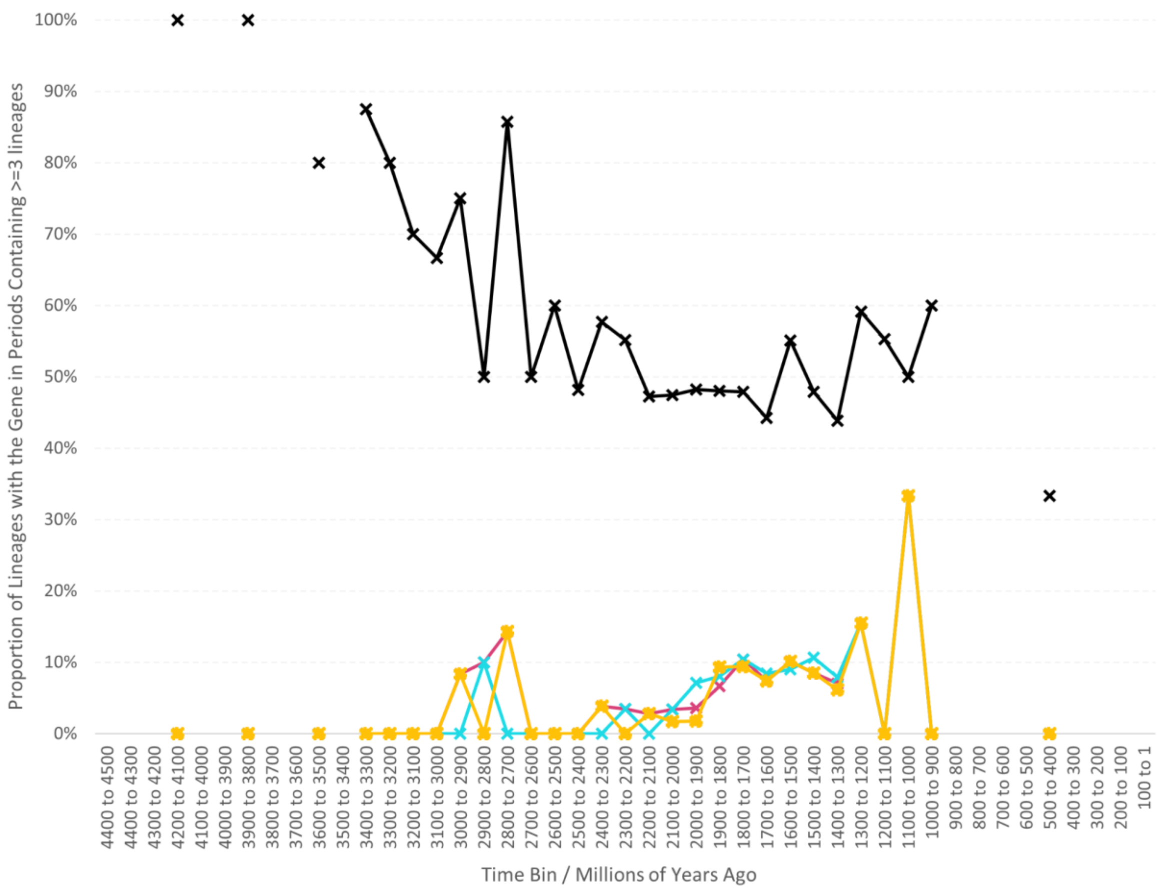

Figure S8: Proportion of lineages estimated to have genes encoding ammonium importers (black line), and three genes for biological nitrogen fixation (coloured lines) with the UGAM clock model. Genes for nitrogen fixation include *nifD* (pink), *nifK* (cyan) and *nifH* (yellow). Genes are assumed to be present in a lineage when their presence probabilities are estimated to be > 50 %. Proportions are not shown for time bins containing less than 3 lineages.

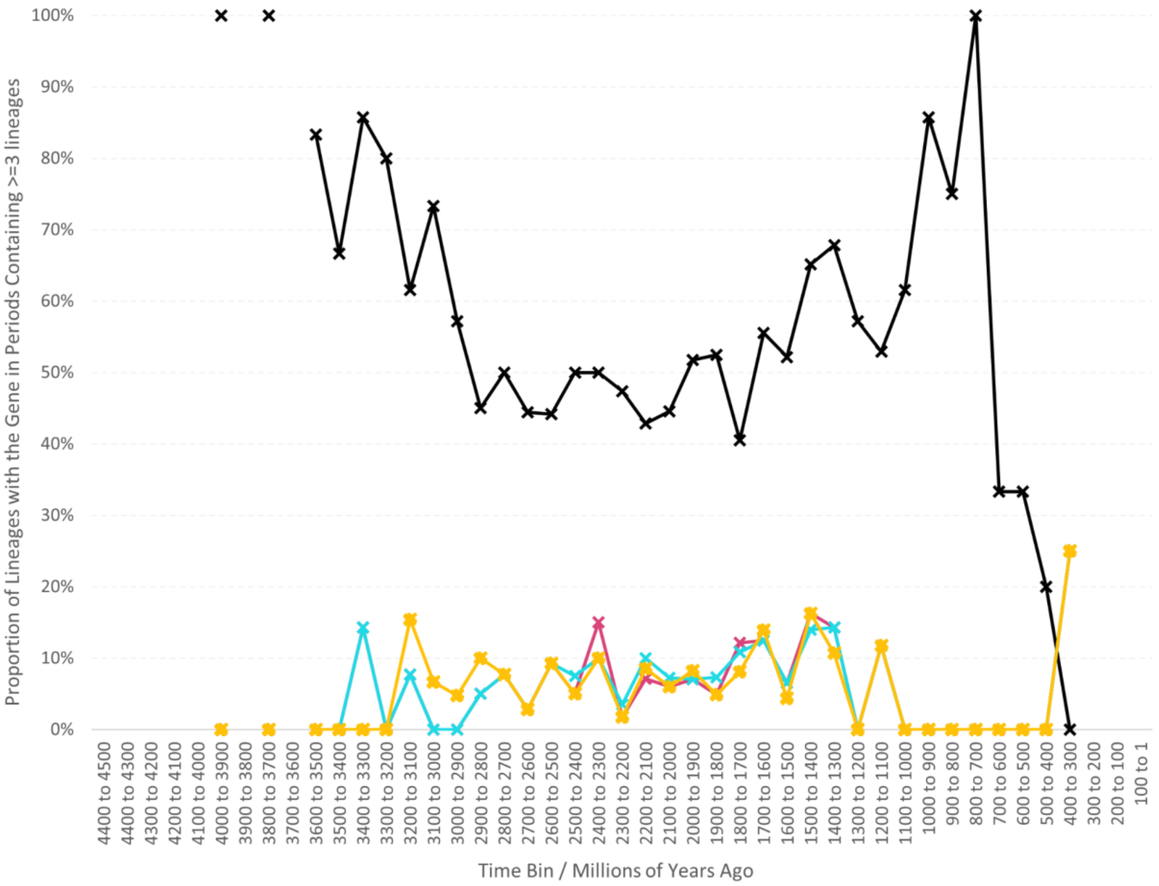

Figure S9: Proportion of lineages estimated to have genes encoding ammonium importers (black line), and three genes for biological nitrogen fixation (coloured lines) with the LN clock model. Genes for nitrogen fixation include *nifD* (pink), *nifK* (cyan) and *nifH* (yellow). Genes are assumed to be present in a lineage when their presence probabilities are estimated to be > 50 %. Proportions are not shown for time bins containing less than 3 lineages.

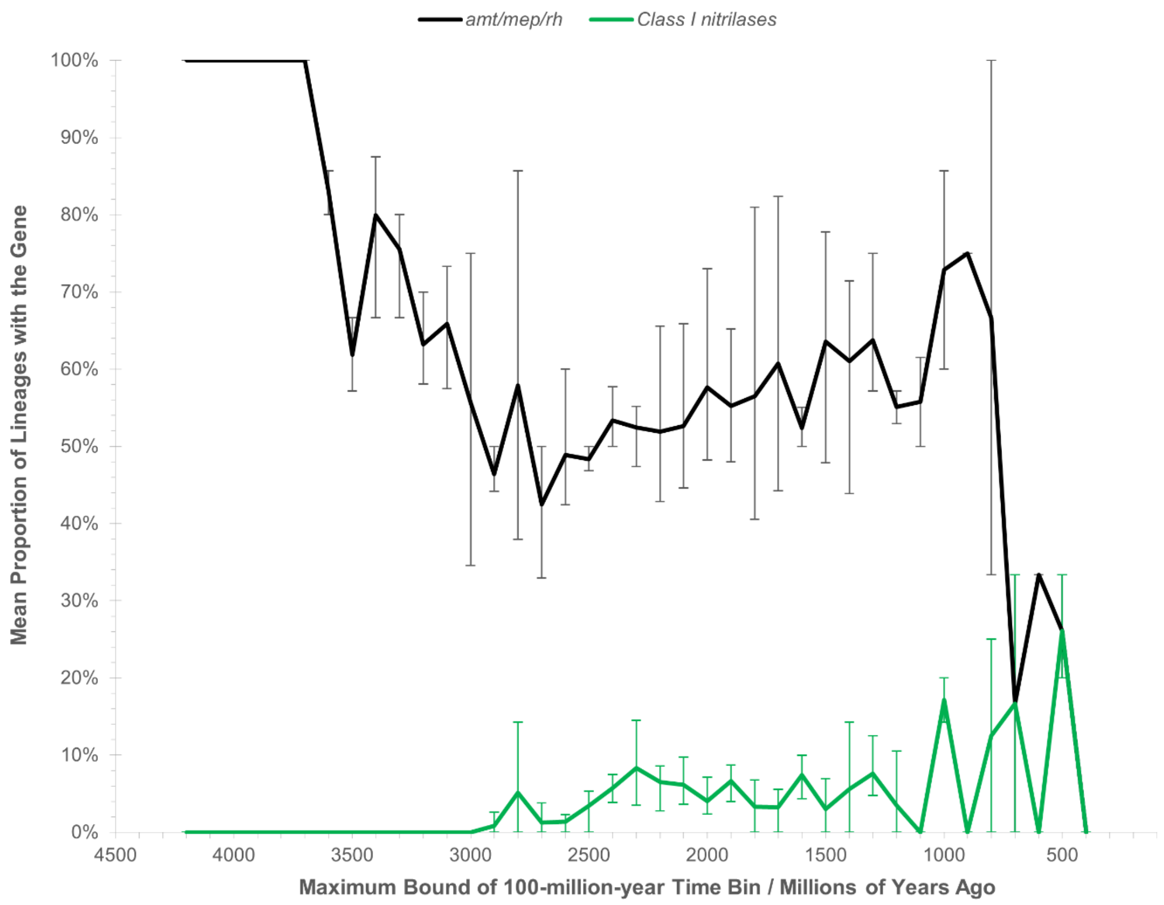

Figure S10: Proportion of microbial lineages with genes for ammonium uptake (black line) and nitrile catabolism (green line) in different time periods. Proportions represent a mean of up to three values representing the number of lineages with the gene divided by the number of lineages in the species tree in a time bin spanning 100 million years. Each value was calculated using results from a different clock model (either CIR, UGAM, or LN). Values were not counted if the time bin contained less than 3 lineages. Error bars represent maximum and minimum proportions. Genes are assumed to be present in a lineage when their presence probabilities are ≥ 50 %.

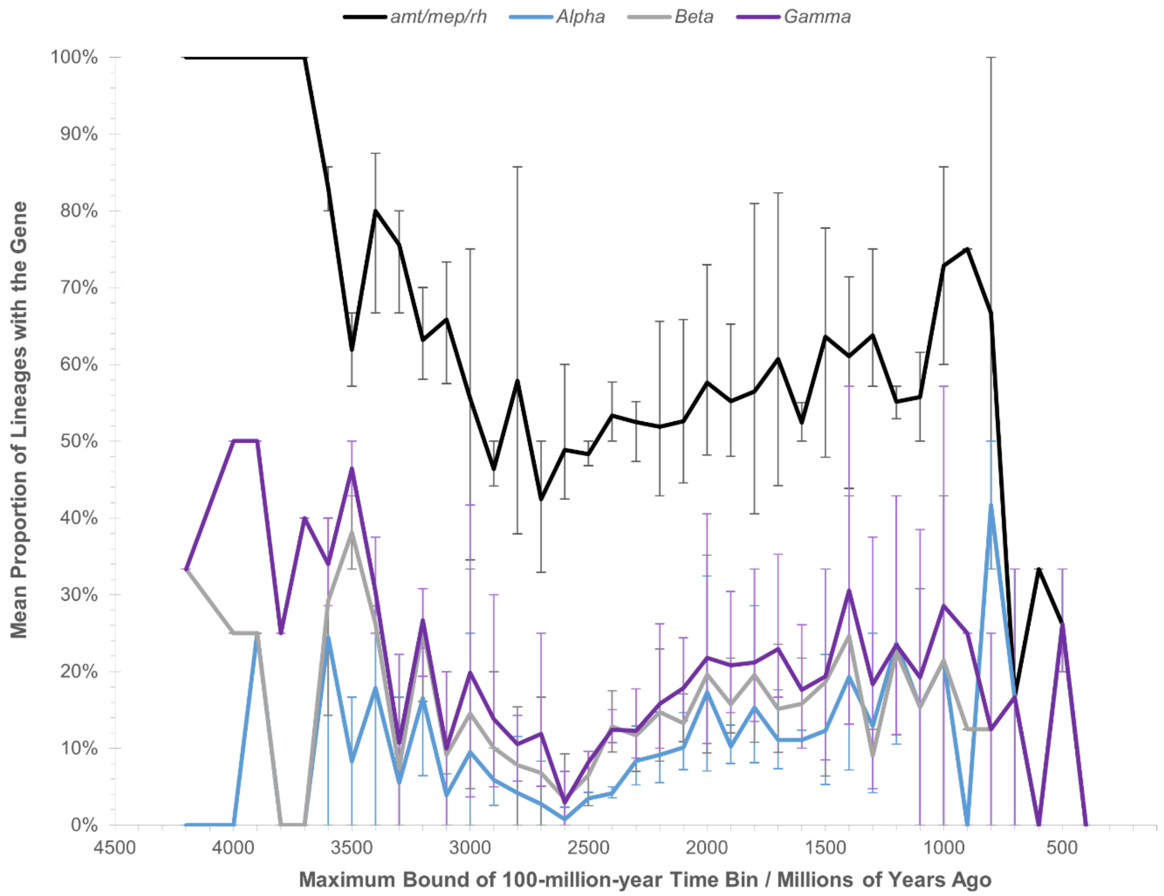

Figure S11: Proportion of microbial lineages with genes for ammonium uptake (black line) and urea catabolism (*ureC* represented by the blue line, *ureB* represented by the grey line, and *ureA* represented by the purple line) in different time periods. Proportions represent a mean of up to three values representing the number of lineages with the gene divided by the number of lineages in the species tree in a time bin spanning 100 million years. Each value was calculated using results from a different clock model (either CIR, UGAM, or LN). Values were not counted if the time bin contained less than 3 lineages. Error bars represent maximum and minimum proportions. Genes are assumed to be present in a lineage when their presence probabilities are > 50 %.

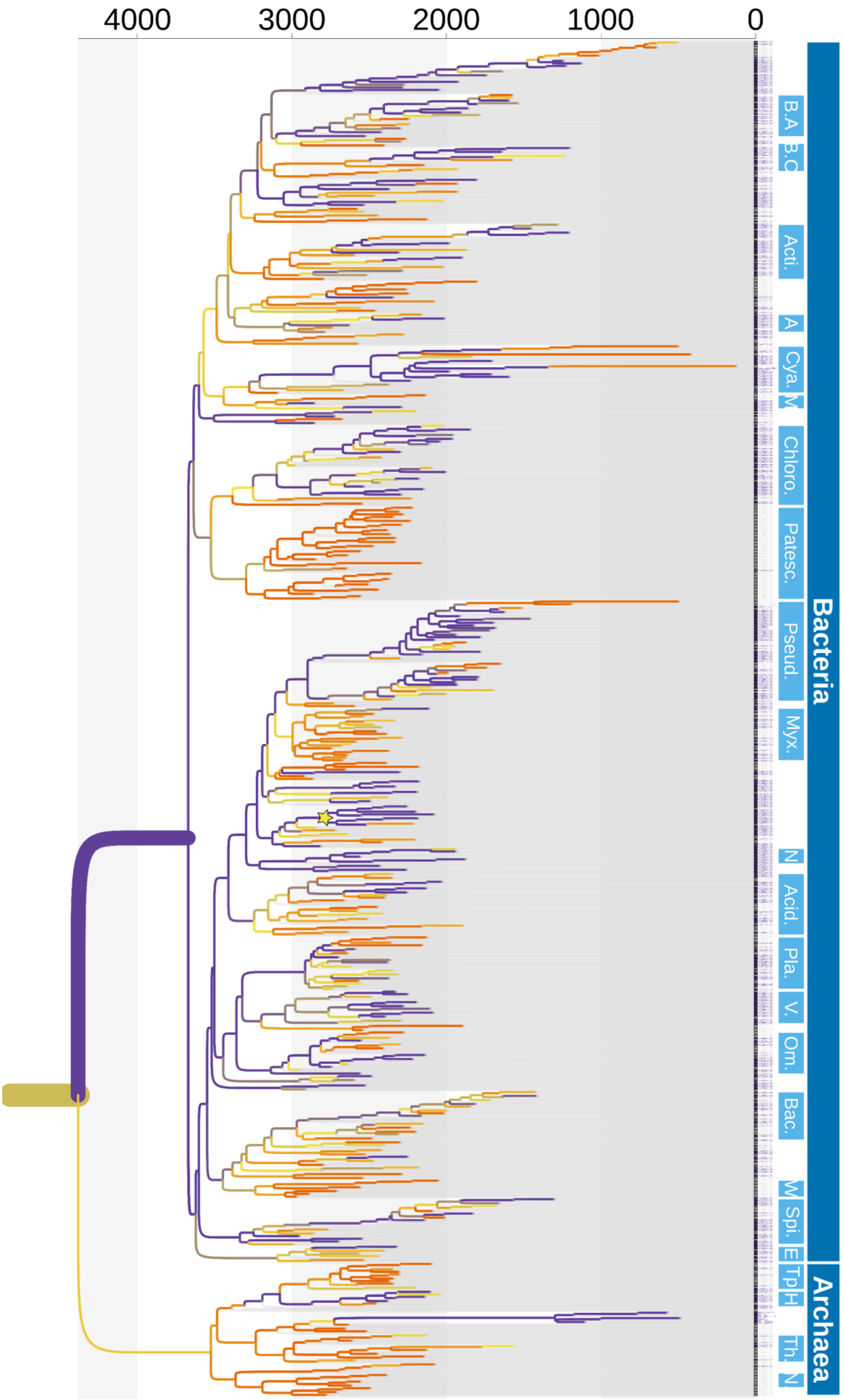

4000
3000
2000
1000
0
B.A
B.C
Acti.
A
Cya.
M
Chloro.
Patesc.
Pseud.
Myx.
N
Acid.
Pla.
V.
Om.
Bac.
W.
Spi.
E
Tp
H
Th.
N.
Bacteria
Archaea

Figure S12: Distribution of genes encoding ammonium transporters from the Amt/Mep/Rh family across the tree of life through time. Leaf labels representing the ID number of each strain's genome, and adjacent boxes are coloured according to whether they encode homologs of Amt/Mep/Rh (purple) or not (grey). Internal branch colours represent presence probabilities of Amt/Mep/Rh estimated with the CIR clock model, on a continuous scale from 100 % probability of the gene being present (purple), to 50 % probability of the gene being present (yellow) to 0 % probability of the gene being present (orange). The thicker the branch, the higher the origination probability for Amt/Mep/Rh.

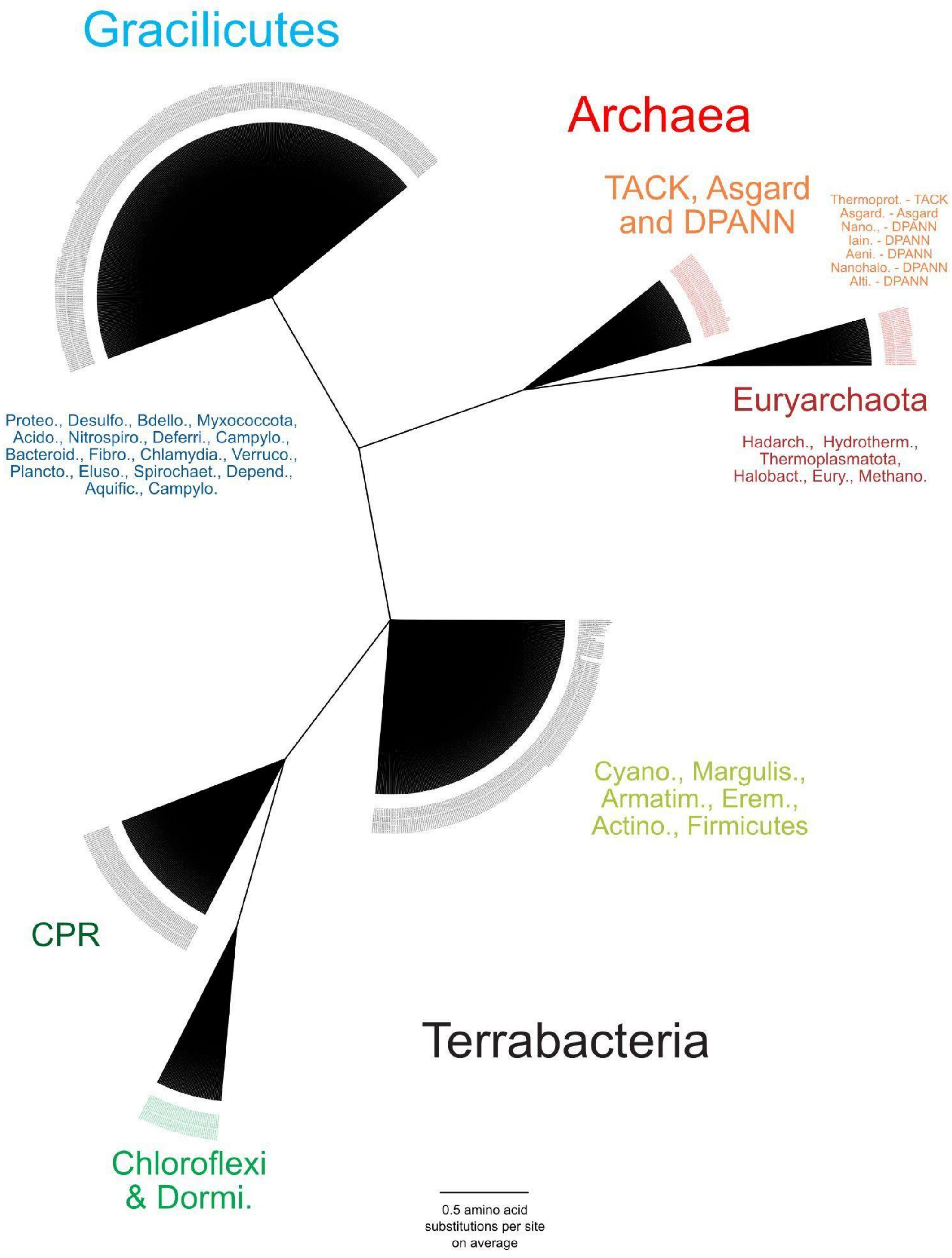

Figure S13: Topological constraints applied to the time-calibrated molecular clock. Coloured text next to wedges describes which phyla they contain, including Archaea (red and orange), Bacteria of two clades; gracilicutes (blue) and terrabacteria (green). The constraint ensures that Euryarchaeota are more closely related to each other than they are to other archaea in-line with previous findings (*101, 103*). Gracilicutes are constrained to be more closely related to each other than to terrabacteria or archaea, and terrabacteria are constrained to be more closely related to each other than to gracilicutes or archaea based on previous

findings (*72, 74-75, 79, 82, 104-105*). Patescibacteriota (formerly CPR) are constrained to be sisters of the Chloroflexi/Dormibacteriota clade within Terrabacteria.

Table S1: Probabilities of catalytic molybdenum nitrogen-fixation genes originating in Bacteria, Archaea, or the Last Universal Common Ancestor (LUCA). Dashes (-) indicate that origination probabilities were not outputted. This happens when no speciation, transfer, loss, or duplication events were sampled during reconciliation analyses, so the gene was likely absent. The probability of each gene originating in the last bacterial common ancestor (LBCA) are also presented.

Gene	Clock Model	Bacteria	Archaea	LUCA	LBCA
nifD	CIR	91%	7%	2%	0%
	UGAM	54%	46%	-	1%
	LN	95%	5%	-	-
nifK	CIR	96%	4%	-	-
	UGAM	92%	7%	1%	2%
	LN	100%	-	-	4%
nifH	CIR	27%	66%	7%	0%
	UGAM	14%	81%	5%	0%
	LN	60%	38%	2%	0%

Table S2: Probabilities of catalytic molybdenum nitrogen-fixation genes originating after the last bacterial common ancestor (LBCA).

Clock Model	*nifD*	*nifK*	*nifH*
CIR	98%	100%	100%
UGAM	99%	97%	95%
LN	100%	100%	98%

Table S3: Estimated timing of the earliest lineage to host *nifD* and *nifK* encoding the N_2-interacting components of nitrogenase.

	CIR	UGAM	LN
Median Age / Ga	3.209	2.813	3.367
Max CI / Ga	3.374	3.283	3.486
Min CI / Ga	3.071	2.311	3.247
Presence Probability of *nifD*	79%	65%	56%
Presence Probability of *nifK*	83%	82%	53%
Predates lineage hosting all three catalytic *nif* genes by;	421 million yrs	941 million yrs	498 million yrs
Ancestral Domain	Bacteria	Bacteria	Bacteria
Ancestral Clade	Gracilicute	Gracilicute	Gracilicute
MRCA of;	Nitrospirota & Nitrospinota	Nitrospirota & Nitrospinota	Nitrospirota & Pseudomonadota

Table S4: Estimated timing of the earliest lineage to have all three catalytic nitrogen-fixing genes (namely *nifD, nifK* and *nifH*).

	CIR	UGAM	LN
Median Age / Ga	2.788	1.872	2.878
Oldest CI / Ga	2.956	2.068	3.040
Youngest CI / Ga	2.623	1.696	2.709
Presence Probability of *nifD*	93%	89%	64%
Presence Probability of *nifK*	99%	99%	90%
Presence Probability of *nifH*	100%	99%	65%
Domain	Bacteria	Bacteria	Bacteria
Clade	Gracilicute	Gracilicute	Terrabacteria
MRCA of;	GCA_016208165.1 and GCA_000429905.1 (a subset of Desulfobacterota)	GCA_016208165.1 & GCA_000429905.1 (a subset of Desulfobacterota)	Bacillota B

Table S5: Estimated timing of the earliest lineage to host *ureC*, *ureB* and *ureA* encoding the catalytic component of urease enzymes for accessing nitrogen from urea. Earliest lineage to host *ureC, ureB* and *ureA* is defined as the oldest lineage with a > 50 % presence probability for all three genes. CI, confidence interval.

	CIR	UGAM	LN
Median Age / Ga	3.275	3.866	3.867
Max CI / Ga	3.442	4.063	3.973
Min CI / Ga	3.122	3.644	3.751
Presence Probability of *ureC*	55%	77%	69%
Presence Probability of *ureB*	62%	97%	93%
Presence Probability of *ureA*	96%	100%	100%
MRCA of;	Cyanobacteria and Sericytochromatia	Cyanobacteria and Actinobacteria	Cyanobacteria and Actinobacteria

Table S6: Estimated timing of the earliest lineage to host a class I nitrilase for catabolizing molecules with C-N triple bonds. Lineages with origination probabilities ≥ 10 % are shown below. CI, confidence interval.

Clock Model	Origin Probability	Median Age / Ga	Oldest CI / Ga	Youngest CI / Ga
CIR	16%	3.384	3.562	3.229
	12%	3.199	3.361	3.053
UGAM	16%	3.308	3.644	2.982
	12%	3.821	4.055	3.562
LN	11%	3.135	3.273	2.992

Table S7: Results of a sensitivity analyses applied to establish whether our search criteria could find homologs of Amt/Mep/Rh genes in the genomes of species that are already known to have them. Annotated genomes of 47 of the 60 strains reported to have Amt/Mep/Rh genes in (19) had their genomes sequenced, annotated, and uploaded to the NCBI. Our search methodology correctly identified at least 1 homolog of Amt/Mep/Rh in all of these genomes.

Strain Discussed in (19)	Annotated Genome Available	GenBank ID of Genome	Amt/Mep/Rh Homologs
Aedes aegypti	Yes	GCA_002204515.2	7
Aedes albopictus	Yes	GCA_035046485.1	8
Alcalicus grahami	No	n/a	n/a
Anablepsoides harti	No	n/a	n/a
Anopheles gambiae	Yes	GCA_943734735.2	2
Aplocheilus lineatus	No	n/a	n/a
Arabidopsis thaliana	Yes	GCA_000001735.2	7
Archaeoglobus fulgidus	Yes	GCA_000008665.1	3
Aspergillus nidulans	Yes	GCA_000011425.1	4
Brassica napus	Yes	GCA_020379485.1	18
Caenorhabditis elegans	Yes	GCA_000002985.3	6
Camellia sinensis	Yes	GCA_004153795.1	11
Candida albicans	Yes	GCA_000182965.3	3
Candidatus Kuenenia stuttgartiensis	Yes	GCA_011066545.1	7
Candidatus Nitrosotalea devenaterra	Yes	GCA_025450535.1	2
Capsicum annuum	Yes	GCA_002878395.1	9
Carcinus maenas	No	n/a	n/a
Chlamydomonas reinhardtii	Yes	GCA_000002595.3	12
Clostridium pasteurianum	Yes	GCA_001856645.1	1
Corynebacterium glutamicum	Yes	GCA_000011325.1	2
Cynodonichthys hildebrandi	No	n/a	n/a
Cyprinus carpio	Yes	GCA_018340385.1	15
Danio rerio	Yes	GCA_049306965.1	26
Dictyostelium discoideum	Yes	GCA_000004695.1	5
Drosophila melanogaster	Yes	GCA_000001215.4	5
Eptatretus stoutii	No	n/a	n/a
Escherichia coli	Yes	GCA_000005845.2	1
Fundulopanchax gardneri	No	n/a	n/a
Glycine max	Yes	GCF_000004515.6	15
Hebeloma cylindrosporum	Yes	GCA_000827355.1	4
Homo sapiens	Yes	GCF_009914755.1	36
Klebsiella pneumoniae	Yes	GCA_000240185.2	1
Kryptolebias marmoratus	Yes	GCA_001649575.2	8
Limulus polyphemus	Yes	GCA_000517525.1	8
Lotus japonicus	Yes	GCA_012489685.1	8
Lycopersicon esculentum	Yes	GCF_036512215.1	9
Macaca mulatta	Yes	GCA_049350105.2	27
Malus domestica	Yes	GCA_042453785.1	13

Manihot esculenta	Yes	GCA_001659605.2	13
Metacarcinus magister	No	n/a	n/a
Methanomassiliicoccus luminyensis	Yes	GCA_000308215.1	2
Mus musculus	Yes	GCA_000001635.27	18
Nitrosomonas europaea	Yes	GCA_000009145.1	1
Oncorhynchus mykiss	Yes	GCA_013265735.2	18
Oryza sativa	Yes	GCA_034140825.1	14
Oryzias latipes	Yes	GCA_002234675.1	12
Paxilus involutus	Yes	GCA_000827475.1	2
Penicillium chrysogenum	Yes	GCA_028827035.1	3
Populus tremulax tremuloides	No	n/a	n/a
Populus trichocarpa	Yes	GCA_000002775.4	18
Portunus trituberculatus	Yes	GCA_017591435.1	6
Pyrus betulaefolia	No	n/a	n/a
Rivulus cylindraceus	No	n/a	n/a
Saccharomyces cerevisiae	Yes	GCA_000146045.2	3
Saccharum officinarum	No	n/a	n/a
Sinonovacula constricta	No	n/a	n/a
Solanum lycopersicum	Yes	GCA_036512215.1	9
Sorghum bicolor	Yes	GCA_000003195.3	10
Takifugu rubripes	Yes	GCF_901000725.2	15
Tuber borchii	Yes	GCA_003070745.1	3

Table S8: Calibration points used in molecular clock analyses. These are conservative estimates, as the origin of a metabolism or group of organisms may predate its first widely accepted expression in the rock record. Note that if one node must be older than a given age, A, then all the ancestors of that node must also be older than A. This is particularly important for the red algal constraint because it also constrains the origin of crown Eukaryotes.

Calibration	Minimum Age	Citation(s)	Maximum Age	Citation (s)	Phylogenetic Placement	Citation(s)
Last Universal Common Ancestor of Life	3.5 Ga	*(113)*	4.4 Ga	*(26-27)*	MRCA of archaea and bacteria	n/a
Methanogenesis	3.46 Ga	*(123-125)*	4.4 Ga	*(26-27)*	MRCA of TACK and Euryarchaeota	*(101, 103, 138)*
Oxygenic photosynthesis	2.32 Ga	*(139)*	3 Ga	*(140)*	MRCA of all extant photosynthetic cyanobacteria (equivalent to crown group Cyanobacteria)	Cyanobacteria are the most successful oxygenic phototrophs among prokaryotes
Eukaryotes (total group)	1.603 Ga	*(141)*	4.4 Ga	*(26-27)*	MRCA of Eukaryotic nuclear genomes and Archaea	n/a
Purple Sulfur Bacteria	1.631 Ga	*(77)*	4.4 Ga	*(26-27)*	MRCA of all extant Chromatiales (equivalent to crown group Chromatiales)	*(77)*
Akinetes	1.6 Ga	*(142)*	4.4 Ga	*(26-27)*	First radiation of all extant heterocyst-forming cyanobacteria	*(120)*
Red algae	1.03 Ga	*(143)*	4.4 Ga	*(26-27)*	MRCA of all red algal nuclear genomes and MRCA of all red algal mitochondrial genomes	*(121)*
Eukaryotes (crown group)	1.03 Ga	*(143)*	4.4 Ga	*(26-27)*	MRCA of all Eukaryotic chloroplasts	n/a
Anoxychlamydiales	1.03 Ga	*(143)*	4.4 Ga	*(26-27)*	MRCA of all Anoxychlamydiales (equivalent to	*(76)*

					crown group Anoxychlamydiales)	
Metazoa	0.635 Ga	*(144)*	0.789 Ga	*(149)*	MRCA of all extant animals (equivalent to crown group animals)	n/a
Embryophyta	0.469 Ga	*(145)*	0.516 Ga	*(145)*	MRCA of all extant bryophytes and tracheophytes	*(145)*
Prymnesiophyte endosymbionts	91 Ma	*(146)*	4.4 Ga	*(26-27)*	MRCA of UCYNA	*(146)*
Hemiaulus endosymbionts	110 Ma	*(147)*	4.4 Ga	*(26-27)*	MRCA of *Richelia intracellularis HH01* and *HM01*	*(148)*

MRCA: Most recent common ancestor

Table S9: Source of query sequences for genome mining of nitrogen genes. Only ‘reviewed’ proteins were used to generate HMM profiles (see methods for details).

Gene	InterPro ID
amt / mep / rh	IPR001905
	IPR018047
	IPR025041
	IPR029020
	COG0004
	Nmar_1547
	PR00909
	PRK10666
	PS01219
	PTHR11730
	TIGR00836
	TIGR03644
nifH	IPR005977
	CD02040
nifK	IPR005975
	IPR005976
	IPR014280
	IPR014281
	CD03466
	CD01971
nifD	IPR005972
	IPR005973
	IPR005974
	IPR010143
Class I Nitrilases	IPR000132
	IPR037544
	IPR044149
	MF03224
	CD07564

Data File S1: Distribution of genes for ammonium uptake, biological nitrogen fixation, nitrile catabolism, and urea catabolism among the bacteria and archaea in our evolutionary trees. GTDB identifiers of these strains are also provided, alongside the domain and phylum that they have been assigned to by the GTDB.

Data File S2: Proportion of lineages estimated to have genes for ammonium uptake, biological nitrogen fixation, nitrile catabolism, and urea catabolism in different time periods. Outputs generated with three molecular clock models are provided, including CIR, UGAM, and LN. These data underly Figure 2, Figure S1, and Figures S7-11.

REFERENCES

1. L. J. Ustick, A. A. Larkin, C. A. Garcia, N. S. Garcia, M. L. Brock, J. A. Lee, N. A. Wiseman, J. K. Moore, A. C. Martiny, Metagenomic analysis reveals global-scale patterns of ocean nutrient limitation. *Science* **372**, 287–291 (2021).

2. C. M. Moore, M. M. Mills, K. R. Arrigo, I. Berman-Frank, L. Bopp, P. W. Boyd, E. D. Galbraith, R. J. Geider, C. Guieu, S. L. Jaccard, T. D. Jickells, J. La Roche, T. M. Lenton, N. M. Mahowald, E. Marañón, I. Marinov, J. K. Moore, T. Nakatsuka, A. Oschlies, M. A. Saito, T. F. Thingstad, A. Tsuda, O. Ulloa, Processes and patterns of oceanic nutrient limitation. *Nat. Geosci.* **6**, 701–710 (2013).

3. P. W. Crockford, Y. M. Bar On, L. M. Ward, R. Milo, I. Halevy, The geologic history of primary productivity. *Curr. Biol.* **33**, 4741–4750.e5 (2023).

4. P. Barth, E. E. Stüeken, C. Helling, L. Rossmanith, Y. Peng, W. Walters, M. Claire, Isotopic constraints on lightning as a source of fixed nitrogen in Earth's early biosphere. *Nat. Geosci.* **16**, 478–484 (2023).

5. J. M. García-Fernández, N. T. de Marsac, J. Diez, Streamlined regulation and gene loss as adaptive mechanisms in *Prochlorococcus* for optimized nitrogen utilization in oligotrophic environments. *Microbiol. Mol. Biol. Rev.* **68**, 630–638 (2004).

6. J. Wang, D. Yan, R. Dixon, Y.-P. Wang, Deciphering the principles of bacterial nitrogen dietary preferences: A strategy for nutrient containment. *MBio* **7**, e00792–16 (2016).

7. G. Williamson, T. Harris, A. Bizior, P. A. Hoskisson, L. Pritchard, A. Javelle, Biological ammonium transporters: Evolution and diversification. *FEBS J.* **291**, 3786–3810 (2024).

8. D. P. Summers, S. Chang, Prebiotic ammonia from reduction of nitrite by iron (II) on the early Earth. *Nature* **365**, 630–633 (1993).

9. M. Nishizawa, T. Saito, A. Makabe, H. Ueda, M. Saitoh, T. Shibuyam K. Takai, Stable abiotic production of ammonia from nitrate in komatiite-hosted hydrothermal systems in the Hadean and Archean oceans. *Minerals* **11**, 321 (2021).

10. W. C. van Heeswijk, H. V. Westerhoff, F. C. Boogerd, Nitrogen assimilation in *Escherichia coli*: Putting molecular data into a systems perspective. *Microbiol. Mol. Biol. Rev.* **77**, 628–695 (2013).

11. G. de Carvalho Fernandes, A. C. Turchetto-Zolet, L. M. P. Passaglia, Glutamine synthetase evolutionary history revisited: Tracing back beyond the last universal common ancestor. *Evolution* **76**, 605–622 (2022).

12. B. H. Patel, C. Percivalle, D. J. Ritson, C. D. Duffy, J. D. Sutherland, Common origins of RNA, protein and lipid precursors in a cyanosulfidic protometabolism. *Nat. Chem.* **7**, 301–307 (2015).

13. M. P. Robertson, S. L. Miller, An efficient prebiotic synthesis of cytosine and uracil. *Nature* **375**, 772–774 (1995).

14. H. J. Cleaves, J. H. Chalmers, A. Lazcano, S. L. Miller, J. L. Bada, A reassessment of prebiotic organic synthesis in neutral planetary atmospheres. *Orig. Life Evol. Biosph.* **38**, 105–115 (2008).

15. S. J. Mojzsis, T. M. Harrison, R. T. Pidgeon, Oxygen-isotope evidence from ancient zircons for liquid water at the Earth's surface 4,300 Myr ago. *Nature* **409**, 178–181 (2001).

16. S. A. Wilde, J. W. Valley, W. H. Peck, C. M. Graham, Evidence from detrital zircons for the existence of continental crust and oceans on the Earth 4.4 Gyr ago. *Nature* **409**, 175–178 (2001).

17. E. R. R. Moody, S. Álvarez-Carretero, T. A. Mahendrarajah, J. W. Clark, H. C. Betts, N. Dombrowski, L. L. Szánthó, R. A. Boyle, S. Daines, X. Chen, N. Lane, Z. Yang, G. A. Shields, G. J. Szöllősi, A. Spang, D. Pisani, T. A. Williams, T. M. Lenton, P. C. J. Donoghue, The nature of the last universal common ancestor and its impact on the early Earth system. *Nat. Ecol. Evol.* **8**, 1654–1666 (2024).

18. P. C. Dos Santos, Z. Fang, S. W. Mason, J. C. Setubal, R. Dixon, Distribution of nitrogen fixation and nitrogenase-like sequences amongst microbial genomes. *BMC Genomics* **13**, 162 (2012).

19. A. K. Garcia, H. McShea, B. Kolaczkowski, B. Kaçar, Reconstructing the evolutionary history of nitrogenases: Evidence for ancestral molybdenum-cofactor utilization. *Geobiology* **18**, 394–411 (2020).

20. C. Parsons, E. E. Stüeken, C. J. Rosen, K. Mateos, R. E. Anderson, Radiation of nitrogen-metabolizing enzymes across the tree of life tracks environmental transitions in Earth history. *Geobiology* **19**, 18–34 (2021).

21. H.-W. Pi, J.-J. Lin, C.-A. Chen, P.-H. Wang, Y.-R. Chiang, C.-C. Huang, C.-C. Young, W.-H. Li, Origin and evolution of nitrogen fixation in prokaryotes. *Mol. Biol. Evol.* **39**, msac181 (2022).

22. F. Mus, D. R. Colman, J. W. Peters, E. S. Boyd, Geobiological feedbacks, oxygen, and the evolution of nitrogenase. *Free Radic. Biol. Med.* **140**, 250–259 (2019).

23. E. R. R. Moody, T. A. Williams, S. Álvarez-Carretero, G. J. Szöllősi, D. Pisani, T. M. Lenton, P. C. J. Donoghue, The emergence of metabolisms through Earth history and implications for biospheric evolution. *Phil. Trans. R. Soc. B* **380**, 20240097 (2025).

24. E. S. Boyd, A. D. Anbar, S. Miller, T. L. Hamilton, M. Lavin, J. W. Peters, A late methanogen origin for molybdenum-dependent nitrogenase. *Geobiology* **9**, 221–232 (2011).

25. K. Mise, Y. Masuda, K. Senoo, H. Itoh, Undervalued pseudo-*nifH* sequences in public databases distort metagenomic insights into biological nitrogen fixers. *mSphere* **6**, e0078521 (2021).

26. P. S. Garcia, S. Gribaldo, G. Borrel, Diversity and evolution of methane-related pathways in archaea. *Annu. Rev. Microbiol.* **76**, 727–755 (2022).

27. K. Kappaun, A. R. Piovesan, C. R. Carlini, R. Ligabue-Braun, Ureases: Historical aspects, catalytic, and non-catalytic properties—A review. *J. Adv. Res.* **13**, 3–17 (2018).

28. S. L. Schwartz, L. T. Rangel, J. G. Payette, G. P. Fournier, A Proterozoic microbial origin of extant cyanide-hydrolyzing enzyme diversity. *Front. Microbiol.* **14**, 1130310 (2023).

29. H. C. Pace, C. Brenner, The nitrilase superfamily: Classification, structure and function. *Genome Biol.* **2**, reviews0001.1 (2001).

30. R. Egelkamp, T. Zimmermann, D. Schneider, R. Hertel, R. Daniel, Impact of nitriles on bacterial communities. *Front. Environ. Sci.* **7**, 103 (2019).

31. G. A. Blackwell, M. Hunt, K. M. Malone, L. Lima, G. Horesh, B. T. F. Alako, N. R. Thomson, Z. Iqbal, Exploring bacterial diversity via a curated and searchable snapshot of archived DNA sequences. *PLoS Biol.* **19**, e3001421 (2021).

32. A. Tomitani, A. H. Knoll, C. M. Cavanaugh, T. Ohno, The evolutionary diversification of Cyanobacteria: Molecular-phylogenetic and paleontological perspectives. *Proc. Natl. Acad. Sci. U.S.A.* **103**, 5442–5447 (2006).

33. H. R. Rucker, B. Kaçar, Enigmatic evolution of microbial nitrogen fixation: Insights from Earth's past. *Trends Microbiol.* **32**, 554–564 (2024).

34. N. Wannicke, E. Stüeken Eva, T. Bauersachs, M. Gehringer, Exploring the influence of atmospheric CO_2 and O_2 levels on the utility of nitrogen isotopes as proxy for biological N_2 fixation. *Appl. Environ. Microbiol.* **90**, e0057424 (2024).

35. E. E. Stüeken, R. Buick, B. M. Guy, M. C. Koehler, Isotopic evidence for biological nitrogen fixation by molybdenum-nitrogenase from 3.2 Gyr. *Nature* **520**, 666–669 (2015).

36. M. Homann, P. Sansjofre, M. Van Zuilen, C. Heubeck, J. Gong, B. Killingsworth, I. S. Foster, A. Airo, M. J. Van Kranendonk, M. Ader, S. V. Lalonde, Microbial life and biogeochemical cycling on land 3,220 million years ago. *Nat. Geosci.* **11**, 665–671 (2018).

37. A. Pellerin, C. Thomazo, M. Ader, J. Marin-Carbonne, J. Alleon, E. Vennin, A. Hofmann, Iron-mediated anaerobic ammonium oxidation recorded in the early Archean ferruginous ocean. *Geobiology* **21**, 277–289 (2023).

38. H.-W. Pi, Y.-R. Chiang, W.-H. Li, Mapping geological events and nitrogen fixation evolution onto the timetree of the evolution of nitrogen-fixation genes. *Mol. Biol. Evol.* **41**, msae023 (2024).

39. R. D. Milton, S. Abdellaoui, N. Khadka, D. R. Dean, D. Leech, L. C. Seefeldt, S. D. Minteer, Nitrogenase bioelectrocatalysis: Heterogeneous ammonia and hydrogen production by MoFe protein. *Energy Environ. Sci.* **9**, 2550–2554 (2016).

40. K. Danyal, A. J. Rasmussen, S. M. Keable, B. S. Inglet, S. Shaw, O. A. Zadvornyy, S. Duval, D. R. Dean, S. Raugei, J. W. Peters, L. C. Seefeldt, Fe protein-independent substrate reduction by nitrogenase MoFe protein variants. *Biochemistry* **54**, 2456–2462 (2015).

41. K. Danyal, B. S. Inglet, K. A. Vincent, B. M. Barney, B. M. Hoffman, F. A. Armstrong, D. R. Dean, L. C. Seefeldt, Uncoupling nitrogenase: Catalytic reduction of hydrazine to ammonia by a MoFe protein in the absence of Fe protein-ATP. *J. Am. Chem. Soc.* **132**, 13197–13199 (2010).

42. K. A. Brown, D. F. Harris, M. B. Wilker, A. Rasmussen, N. Khadka, H. Hamby, S. Keable, G. Dukovic, J. W. Peters, L. C. Seefeldt, P. W. King, Light-driven dinitrogen reduction catalyzed by a CdS:nitrogenase MoFe protein biohybrid. *Science* **352**, 448–450 (2016).

43. J. P. Riley, D. Taylor, The concentrations of cadmium, copper, iron, manganese, molybdenum, nickel, vanadium and zinc in part of the tropical north-east Atlantic ocean. *Deep-Sea Res. Oceanogr. Abstr.* **19**, 307–317 (1972).

44. J. A. Brandes, N. Z. Boctor, G. D. Cofy, B. A. Cooper, R. M. Hazen, H. S. Yoder, Abiotic nitrogen reduction on the early Earth. *Nature* **395**, 365–367 (1998).

45. A. Smirnov, D. Hausner, R. Laffers, D. R. Strongin, M. A. A. Schoonen, Abiotic ammonium formation in the presence of Ni-Fe metals and alloys and its implications for the Hadean nitrogen cycle. *Geochem. Trans.* **9**, 5 (2008).

46. E. E. Stüeken, F. S. M. Holland, S. Mikhail, Igneous rocks as a viable source of fixed nitrogen to the prebiotic world. *Geochem. Perspect. Lett.* **35**, 13–17 (2025).

47. T. A. Mather, D. M. Pyle, A. G. Allen, Volcanic source for fixed nitrogen in the early Earth's atmosphere. *Geology* **32**, 905–908 (2004).

48. R. Navarro-González, C. P. McKay, D. N. Mvondo, A possible nitrogen crisis for Archaean life due to reduced nitrogen fixation by lightning. *Nature* **412**, 61–64 (2001).

49. B. W. Johnson, E. E. Stüeken, in *Treatise on Geochemistry*, A. Anbar, D. Weis, Eds. (Elsevier, ed. 3, 2025), pp. 177–201.

50. D. L. Pinti, K. Hashizume, J.-i. Matsuda, Nitrogen and argon signatures in 3.8 to 2.8 Ga metasediments: Clues on the chemical state of the Archean ocean and the deep biosphere. *Geochim. Cosmochim. Acta* **65**, 2301–2315 (2001).

51. W.-L. Wang, J. K. Moore, A. C. Martiny, F. W. Primeau, Convergent estimates of marine nitrogen fixation. *Nature* **566**, 205–211 (2019).

52. F. Tian, J. F. Kasting, K. Zahnle, Revisiting HCN formation in Earth's early atmosphere. *Earth Planet. Sci. Lett.* **308**, 417–423 (2011).

53. Z. R. Todd, K. I. Öberg, Cometary delivery of hydrogen cyanide to the early Earth. *Astrobiology* **20**, 1109–1120 (2020).

54. M. Ferus, S. Civiš, A. Mládek, J. Šponer, L. Juha, J. E. Šponer, On the Road from Formamide Ices to Nucleobases: IR-spectroscopic observation of a direct reaction between cyano radicals and formamide in a high-energy impact event. *J. Am. Chem. Soc.* **134**, 20788–20796 (2012).

55. F. S. Brigiano, Y. Jeanvoine, A. Largo, R. Spezia, The formation of urea in space I. Ion-molecule, neutral-neutral, and radical gas-phase reactions. *Astron. Astrophys.* **610**, A26 (2018).

56. D. M. Ratnayake, R. Tanaka, E. Nakamura, Biogeochemical impact of nickel and urea in the great oxidation event. *Commun. Earth Environ.* **6**, 654 (2025).

57. M. A. Mohajer, P. Basuri, A. Evdokimov, G. David, D. Zindel, E. Miliordos, R. Signorell, Spontaneous formation of urea from carbon dioxide and ammonia in aqueous droplets. *Science* **388**, 1426–1430 (2025).

58. P. Kharecha, J. Kasting, J. Siefert, A coupled atmosphere–ecosystem model of the early Archean Earth. *Geobiology* **3**, 53–76 (2005).

59. D. E. Canfield, M. T. Rosing, C. Bjerrum, Early anaerobic metabolisms. *Phil. Trans. R. Soc. B* **361**, 1819–1836 (2006).

60. L. M. Ward, B. Rasmussen, W. W. Fischer, Primary productivity was limited by electron donors prior to the advent of oxygenic photosynthesis. *J. Geophys. Res. Biogeo.* **124**, 211–226 (2019).

61. G. Bianchini, M. Hagemann, P. Sánchez-Baracaldo, Stochastic character mapping, bayesian model selection, and biosynthetic pathways shed new light on the evolution of habitat preference in Cyanobacteria. *Syst. Biol.* **73**, 644–665 (2024).

62. J. S. Boden, K. O. Konhauser, L. J. Robbins, P. Sánchez-Baracaldo, Timing the evolution of antioxidant enzymes in Cyanobacteria. *Nat. Commun.* **12**, 4742 (2021).

63. G. P. Fournier, K. R. Moore, L. T. Rangel, J. G. Payette, L. Momper, T. Bosak, The Archean origin of oxygenic photosynthesis and extant cyanobacterial lineages. *Proc. R. Soc. B* **288**, 20210675 (2021).

64. S. Viehmann, E. E. Stüeken, S. V. Hohl, N. Tepe, Y. Lin, D. Kraemer, M. V. Kranendonk, J. Krayer, D. M. Ernst, S. Weyer, Europium traces the impact of high temperature hydrothermal systems on the early oceans. *Geochem. Perspect. Lett.* **34**, 57–61 (2025).

65. D. H. Parks, M. Chuvochina, P. A. Chaumeil, C. Rinke, A. J. Mussig, P. Hugenholtz, A complete domain-to-species taxonomy for bacteria and archaea. *Nat. Biotechnol.* **38**, 1079–1086 (2020).

66. D. H. Parks, M. Chuvochina, D. W. Waite, C. Rinke, A. Skarshewski, P. A. Chaumeil, P. Hugenholtz, A standardized bacterial taxonomy based on genome phylogeny substantially revises the tree of life. *Nat. Biotechnol.* **36**, 996–1004 (2018).

67. A. Chklovski, D. H. Parks, B. J. Woodcroft, G. W. Tyson, CheckM2: A rapid, scalable and accurate tool for assessing microbial genome quality using machine learning. *Nat. Methods* **20**, 1203–1212 (2023).

68. C. A. Martinez-Gutierrez, F. O. Aylward, Phylogenetic signal, congruence, and uncertainty across bacteria and archaea. *Mol. Biol. Evol.* **38**, 5514–5527 (2021).

69. G. A. Coleman, A. A. Davin, T. A. Mahendrarajah, L. L. Szantho, A. Spang, P. Hugenholtz, G. J. Szöllősi, T. A. Williams, A rooted phylogeny resolves early bacterial evolution. *Science* **372**, eabea0511 (2021).

70. C. W. Stairs, J. E. Dharamshi, D. Tamarit, L. Eme, S. L. Jørgensen, A. Spang, T. J. G. Ettema, Chlamydial contribution to anaerobic metabolism during eukaryotic evolution. *Sci. Adv.* **6**, eabb7258 (2020).

71. J. J. Brocks, P. Schaeffer, Okenane, a biomarker for purple sulfur bacteria (Chromatiaceae), and other new carotenoid derivatives from the 1640Ma Barney Creek Formation. *Geochim. Cosmochim. Acta* **72**, 1396–1414 (2008).

72. S. Sunagawa, D. R. Mende, G. Zeller, F. Izquierdo-Carrasco, S. A. Berger, J. R. Kultima, L. Coelho, M. Arumugam, J. Tap, H. Nielsen, S. Rasmussen, S. Brunak, O. Pedersen, F. Guarner, W. de Vos, J. Wang, J. Li, J. Doré, S. Ehrlich, A. Stamatakis, P. Bork, Metagenomic species profiling using universal phylogenetic marker genes. *Nat. Methods* **10**, 1196–1199 (2013).

73. L. A. Hug, B. J. Baker, K. Anantharaman, C. T. Brown, A. J. Probst, C. J. Castelle, C. N. Butterfield, A. W. Hernsdorf, Y. Amano, K. Ise, Y. Suzuki, N. Dudek, S. A. Relman, K. M. Finstad, R. Amundson, B. C. Thomas, J. F. Banfield, A new view of the tree of life. *Nat. Microbiol.* **1**, 16048 (2016).

74. H. C. Betts, M. N. Puttick, J. W. Clark, T. A. Williams, P. C. J. Donoghue, D. Pisani, Integrated genomic and fossil evidence illuminates life's early evolution and eukaryote origin. *Nat. Ecol. Evol.* **2**, 1556–1562 (2018).

75. T. A. Williams, C. J. Cox, P. G. Foster, G. J. Szöllosi, T. M. Embley, Phylogenomics provides robust support for a two-domains tree of life. *Nat. Ecol. Evol.* **4**, 138–147 (2020).

76. E. R. R. Moody, T. A. Mahendrarajah, N. Dombrowski, J. W. Clark, C. Petitjean, P. Offre, G. J. Szöllősi, A. Spang, T. A. Williams, An estimate of the deepest branches of the tree of life from ancient vertically evolving genes. *eLife* **11**, e66695 (2022).

77. S. R. Eddy, Accelerated Profile HMM Searches. *PLoS Comput. Biol.* **7**, e1002195 (2011).

78. J. Mistry, S. Chuguransky, L. Williams, M. Qureshi, G. A. Salazar, E. L. L. Sonnhammer, S. C. E. Tosatto, L. Paladin, S. Raj, L. J. Richardson, R. D. Finn, A. Bateman, Pfam: The protein families database in 2021. *Nucleic Acids Res.* **49**, D412–D419 (2021).

79. K. Katoh, D. M. Standley, MAFFT multiple sequence alignment software version 7: Improvements in performance and usability. *Mol. Biol. Evol.* **30**, 772–780 (2013).

80. C. Zhang, Y. Zhao, E. L. Braun, S. Mirarab, TAPER: Pinpointing errors in multiple sequence alignments despite varying rates of evolution. *Methods Ecol. Evol.* **12**, 2145–2158 (2021).

81. S. Capella-Gutierrez, J. M. Silla-Martinez, T. Gabaldon, trimAl: A tool for automated alignment trimming in large-scale phylogenetic analyses. *Bioinformatics* **25**, 1972–1973 (2009).

82. C. A. Martinez-Gutierrez, J. C. Uyeda, F. O. Aylward, A timeline of bacterial and archaeal diversification in the ocean. *eLife* **12**, RP88268 (2023).

83. M. N. Price, P. S. Dehal, A. P. Arkin, FastTree 2-approximately maximum-likelihood trees for large alignments. *PLOS ONE* **5**, e9490 (2010).

84. G. Bianchini, P. Sánchez-Baracaldo, TreeViewer: Flexible, modular software to visualise and manipulate phylogenetic trees. *Ecol. Evol.* **14**, e10873 (2024).

85. B. Q. Minh, H. A. Schmidt, O. Chernomor, D. Schrempf, M. D. Woodhams, A. von Haeseler, R. Lanfear, IQ-TREE 2: New models and efficient methods for phylogenetic inference in the genomic era. *Mol. Biol. Evol.* **37**, 1530–1534 (2020).

86. D. T. Hoang, O. Chernomor, A. von Haeseler, B. Q. Minh, L. S. Vinh, UFBoot2: Improving the ultrafast bootstrap approximation. *Mol. Biol. Evol.* **35**, 518–522 (2017).

87. B. Q. Minh, C. C. Dang, L. S. Vinh, R. Lanfear, QMaker: Fast and accurate method to estimate empirical models of protein evolution. *Syst. Biol.* **70**, 1046–1060 (2021).

88. C. C. Dang, L. S. Vinh, Estimating amino acid substitution models and rooting bacterial trees. *J. Comput. Sci. Cybern.* **40**, 53–66 (2024).

89. S. Kalyaanamoorthy, B. Q. Minh, T. K. F. Wong, A. von Haeseler, L. S. Jermiin, ModelFinder: Fast model selection for accurate phylogenetic estimates. *Nat. Methods* **14**, 587–589 (2017).

90. C. Rinke, M. Chuvochina, A. J. Mussig, P.-A. Chaumeil, A. A. Davín, D. W. Waite, W. B. Whitman, D. H. Parks, P. Hugenholtz, A standardized archaeal taxonomy for the Genome Taxonomy Database. *Nat. Microbiol.* **6**, 946–959 (2021).

91. O. Chernomor, A. von Haeseler, B. Q. Minh, Terrace aware data structure for phylogenomic inference from supermatrices. *Syst. Biol.* **65**, 997–1008 (2016).

92. P. Lopez, D. Casane, H. Philippe, Heterotachy, an important process of protein evolution. *Mol. Biol. Evol.* **19**, 1–7 (2002).

93. S. Guindon, J. F. Difayard, V. Lefort, M. Anisimova, W. Hordijk, O. Gascuel, New algorithms and methods to estimate maximum-likelihood phylogenies: Assessing the performance of PhyML 3.0. *Syst. Biol.* **59**, 307–321 (2010).

94. A. Rambaut, FigTree version 1.4.4 (Institute of Evolutionary Biology, University of Edinburgh, 2018.

95. T. A. Williams, G. J. Szöllősi, A. Spang, P. G. Foster, S. E. Heaps, B. Boussau, T. J. G. Ettema, T. Embley, Integrative modeling of gene and genome evolution roots the archaeal tree of life. *Proc. Natl. Acad. Sci. U.S.A.* **114**, E4602–E4611 (2017).

96. Q. Zhu, U. Mai, W. Pfeiffer, G. Janssen, K. R. Asnicar, J. G. Sanders, S. Belda-Ferre, G. A. Al-Ghalith, M. Kopylova, E. J. McDonald, T. Kosciolek, J. T. Morton, K. C. S. Carini, S. M. S. Malik, A. J. S. Gilbert, D. Knight, G. J. S. Knight, R. Knight, Phylogenomics of 10,575 genomes reveals evolutionary proximity between domains Bacteria and Archaea. *Nat. Commun.* **10**, 5477 (2019).

97. Y. Wang, G. Wegener, T. A. Williams, R. Xie, J. Hou, C. Tian, Y. Zhang, F. Wang, X. Xiao, A methylotrophic origin of methanogenesis and early divergence of anaerobic multicarbon alkane metabolism. *Sci. Adv.* **7**, eabj1453 (2021).

98. J. Witwinowski, A. Sartori-Rupp, N. Taib, N. Pende, T. N. Tham, D. Poppleton, J.-M. Ghigo, C. Beloin, S. Gribaldo, An ancient divide in outer membrane tethering systems in bacteria suggests a mechanism for the diderm-to-monoderm transition. *Nat. Microbiol.* **7**, 411–422 (2022).

99. N. Taib, D. Megrian, J. Witwinowski, P. Adam, D. Poppleton, G. Borrel, C. Beloin, S. Gribaldo, Genome-wide analysis of the Firmicutes illuminates the diderm/monoderm transition. *Nat. Ecol. Evol.* **4**, 1661–1672 (2020).

100. C. J. Castelle, J. F. Banfield, Major new microbial groups expand diversity and alter our understanding of the tree of life. *Cell* **172**, 1181–1197 (2018).

101. J. S. Boden, J. Zhong, R. E. Anderson, E. E. Stüeken, Timing the evolution of phosphorus-cycling enzymes through geological time using phylogenomics. *Nat. Commun.* **15**, 3703 (2024).

102. N. Lartillot, T. Lepage, S. Blanquart, PhyloBayes 3: A Bayesian software package for phylogenetic reconstruction and molecular dating. *Bioinformatics* **25**, 2286–2288 (2009).

103. L. S. Quang, O. Gascuel, N. Lartillot, Empirical profile mixture models for phylogenetic reconstruction. *Bioinformatics* **24**, 2317–2323 (2008).

104. T. Lepage, D. Bryant, H. Philippe, N. Lartillot, A general comparison of relaxed molecular clock models. *Mol. Biol. Evol.* **24**, 2669–2680 (2007).

105. J. L. Thorne, H. Kishino, I. S. Painter, Estimating the rate of evolution of the rate of molecular evolution. *Mol. Biol. Evol.* **15**, 1647–1657 (1998).

106. A. J. Drummond, S. Y. W. Ho, M. J. Phillips, A. Rambaut, Relaxed phylogenetics and dating with confidence. *PLoS Biol.* **4**, 699–710 (2006).

107. F. Westall, A. Brack, A. G. Fairén, M. D. Schulte, Setting the geological scene for the origin of life and continuing open questions about its emergence. *Front. Astron. Space Sci.* **9**, 1095701 (2023).

108. A. Nishihara, Y. Tsukatani, C. Azai, M. K. Nobu, Illuminating the coevolution of photosynthesis and bacteria. *Proc. Natl. Acad. Sci. U.S.A.* **121**, e2322120121 (2024).

109. Z. C. Zeng, L. Y. Li, H. Wang, Y. X. Tao, Z. B. Lv, F. P. Wang, Y. Z. Wang, Oxidative adaptations in prokaryotes imply the oxygenic photosynthesis before crown-group Cyanobacteria. *PNAS Nexus* **4**, pgaf035 (2025).

110. K. Mateos, G. Chappell, A. Klos, B. Le, J. Boden, E. E. Stüeken, R. Anderson, The evolution and spread of sulfur cycling enzymes reflect the redox state of the early Earth. *Sci. Adv.* **9**, eade4847 (2023).

111. P. Sánchez-Baracaldo, Origin of marine planktonic Cyanobacteria. *Sci. Rep.* **5**, 17418 (2015).

112. P. Sánchez-Baracaldo, G. Bianchini, A. Di Cesare, C. Callieri, N. A. M. Chrismas, Insights Into the evolution of picocyanobacteria and phycoerythrin genes (*mpeBA* and *cpeBA*). *Front. Microbiol.* **10**, 45 (2019).

113. P. Sánchez-Baracaldo, T. Cardona, On the origin of oxygenic photosynthesis and Cyanobacteria. *New Phytol.* **225**, 1440–1446 (2020).

114. P. Sánchez-Baracaldo, P. K. Hayes, C. E. Blank, Morphological and habitat evolution in the Cyanobacteria using a compartmentalization approach. *Geobiology* **3**, 145–165 (2005).

115. P. Sánchez-Baracaldo, J. A. Raven, D. Pisani, A. H. Knoll, Early photosynthetic eukaryotes inhabited low-salinity habitats. *Proc. Natl. Acad. Sci. U.S.A.* **114**, E7737-E7745 (2017).

116. P. Sánchez-Baracaldo, A. Ridgwell, J. A. Raven, A Neoproterozoic transition in the marine nitrogen cycle. *Curr. Biol.* **24**, 652–657 (2014).

117. J. M. Wolfe, G. P. Fournier, Horizontal gene transfer constrains the timing of methanogen evolution. *Nat. Ecol. Evol.* **2**, 897–903 (2018).

118. B. Cavalazzi, L. Lemelle, A. Simionovici, S. L. Cady, M. J. Russell, E. Bailo, R. Canteri, E. Enrico, A. Manceau, A. Maris, M. Salome, E. Thomassot, N. Bouden, R. Tucoulou, A. Hofmann, Cellular remains in a ~3.42-billion-year-old subseafloor hydrothermal environment. *Sci. Adv.* **7**, eabf3963 (2021).

119. Y. Ueno, K. Yamada, N. Yoshida, S. Maruyama, Y. Isozaki, Evidence from fluid inclusions for microbial methanogenesis in the early Archaean era. *Nature* **440**, 516–519 (2006).

120. A. Rambaut, A. J. Drummond, D. Xie, G. Baele, M. A. Suchard, Posterior summarization in bayesian phylogenetics using Tracer 1.7. *Syst. Biol.* **67**, 901–904 (2018).

121. M. Blum, A. Andreeva, L. C. Florentino, S. R. Chuguransky, T. Grego, E. Hobbs, B. L. Pinto, A. Orr ,T. Paysan-Lafosse, I. Ponamareva, G. A. Salazar, N. Bordin, P. Bork, A. Bridge, L. Colwell, J. Gough, D. H. Haft, I. Letunic, F. Llinares-López, A. Marchler-Bauer, L. Meng-Papaxanthos, H. Mi, D. A. Natale, C. A. Orengo, A. P. Pandurangan, D. Piovesan, C. Rivoire, C. J. A. Sigrist, N. Thanki, F. Thibaud-Nissen, P. D. Thomas, S. C. E. Tosatto, C. H. Wu, A. Bateman, InterPro: The protein sequence classification resource in 2025. *Nucleic Acids Res.* **53**, D444–D456 (2025).

122. M. Fukuda, H. Takeda, H. E. Kato, S. Doki, K. Ito, A. D. Maturana, R. Ishitani, O. Nureki, Structural basis for dynamic mechanism of nitrate/nitrite antiport by NarK. *Nat. Commun.* **6**, 7097 (2015).

123. J. Hallgren, K. D. Tsirigos, M. D. Pedersen, J. J. A. Armenteros, P. Marcatili, H. Nielsen, A. Krogh, O. Winther, DeepTMHMM predicts alpha and beta transmembrane proteins using deep neural networks. bioRxiv 487609 [Preprint] (2022).

124. J. S. Boden, S. M. Som, W. J. Brazelton, R. E. Anderson, E. E. Stüeken, Evaluating serpentinization as a source of phosphite to microbial communities in hydrothermal vents. *Geobiology* **23**, e70016 (2025).

125. S.-C. Chen, G.-X. Sun, Y. Yan, K. T. Konstantinidis, S.-Y. Zhang, Y. Deng, X.-M. Li, H.-L. Cui, F. Musat, D. Popp, B. P. Rosen, Y.-G. Zhu, The Great Oxidation Event expanded the genetic repertoire of arsenic metabolism and cycling. *Proc. Natl. Acad. Sci. U.S.A.* **117**, 10414–10421 (2020).

126. F. D. K. Tria, G. Landan, T. Dagan, Phylogenetic rooting using minimal ancestor deviation. *Nat. Ecol. Evol.* **1**, 0193 (2017).

127. T. Wade, L. T. Rangel, S. Kundu, G. P. Fournier, M. S. Bansal, Assessing the accuracy of phylogenetic rooting methods on prokaryotic gene families. *PLOS ONE* **15**, e0232950 (2020).

128. A. K. Garcia, B. Kolaczkowski, B. Kaçar, Reconstruction of nitrogenase predecessors suggests origin from maturase-like proteins. *Genome Biol. Evol.* **14**, evac031 (2022).

129. C. C. Lee, K. Górecki, M. Stang, M. W. Ribbe, Y. Hu, Cofactor maturase NifEN: A prototype ancient nitrogenase? *Sci. Adv.* **10**, eado6169 (2024).

130. F. Ronquist, M. Teslenko, P. van der Mark, D. L Ayres, A. Darling, S. Hohna, B. Larget, L. Liu, M. A. Suchard, J. P. Huelsenbeck, MrBayes 3.2: Efficient bayesian phylogenetic inference and model choice across a large model space. *Syst. Biol.* **61**, 539–542 (2012).

131. B. Morel, T. A. Williams, A. Stamatakis, G. J. Szöllosi, AleRax: A tool for gene and species tree co-estimation and reconciliation under a probabilistic model of gene duplication, transfer, and loss. *Bioinformatics* **40**, btae162 (2024).

132. R. Navarro-Gonzalez, M. J. Molina, L. T. Molina, Nitrogen fixation by volcanic lightning in the early Earth. *Geophys. Res. Lett.* **25**, 3123–3126 (1998).

133. J. F. Kasting, J. C. G. Walker, Limits on oxygen concentration in the prebiological atmosphere and the rate of abiotic fixation of nitrogen. *J. Geophys. Res. Oceans* **86**, 1147–1158 (1981).

134. J. F. Kasting, Bolide impacts and the oxidation-state of carbon in the Earth's early atmosphere. *Orig. Life Evol. Biosph.* **20**, 199–231 (1990).

135. H. Nakazawa, T. Sekine, T. Kakegawa, S. Nakazawa, High yield shock synthesis of ammonia from iron, water and nitrogen available on the early Earth. *Earth Planet. Sci. Lett.* **235**, 356–360 (2005).

136. N. F. Wogan, D. C. Catling, K. J. Zahnle, R. Lupu, Origin-of-life molecules in the atmosphere after big impacts on the early Earth. *Planet. Sci. J.* **4**, 169 (2023).

137. D. E. Canfield, A. N. Glazer, P. G. Falkowski, The evolution and future of Earth's nitrogen cycle. *Science* **330**, 192–196 (2010).

138. E. K. Moore, B. I. Jelen, D. Giovannelli, H. Raanan, P. G. Falkowski, Metal availability and the expanding network of microbial metabolisms in the Archaean eon. *Nat. Geosci.* **10**, 629–636 (2017).

139. A. Bekker, H. D. Holland, P. L. Wang, D. Rumble, H. J. Stein, J. L. Hannah, L. L. Coetzee, N. J. Beukes, Dating the rise of atmospheric oxygen. *Geochim. Cosmochim. Acta* **68**, A780-A780 (2004).

140. T. Bosak, B. Liang, M. S. Sim, A. P. Petroff, Morphological record of oxygenic photosynthesis in conical stromatolites. *Proc. Natl. Acad. Sci. U.S.A.* **106**, 10939–10943 (2009).

141. K. Pang, Q. Tang, J. D. Schiffbauer, J. Yao, X. Yuan, B. Wan, L. Chen, Z. Ou, S. Xiao, The nature and origin of nucleus-like intracellular inclusions in Paleoproterozoic eukaryote microfossils. *Geobiology* **11**, 499–510 (2013).

142. S. Golubic, V. N. Sergeev, A. H. Knoll, Mesoproterozoic *Archaeoellipsoides*: Akinetes of heterocystous cyanobacteria. *Lethaia* **28**, 285–298 (1995).

143. T. M. Gibson, P. M. Shih, V. M. Cumming, W. W. Fischer, P. W. Crockford, M. S. W. Hodgskiss, S. Worndle, R. A. Creaser, R. H. Rainbird, T. M. Skulski, G. P. Halverson, Precise age of *Bangiomorpha pubescens* dates the origin of eukaryotic photosynthesis. *Geology* **46**, 135–138 (2018).

144. J. A. Zumberge, G. D. Love, P. Cárdenas, E. A. Sperling, S. Gunasekera, M. Rohrssen, E. Grosjean, J. P. Grotzinger, R. E. Summons, Demosponge steroid biomarker 26-methylstigmastane provides evidence for Neoproterozoic animals. *Nat. Ecol. Evol.* **2**, 1709–1714 (2018).

145. J. L. Morris, M. N. Puttick, J. W. Clark, D. Edwards, P. Kenrick, S. Pressel, C. H. Wellman, Z. Yang, H. Schneider, P. C. J. Donoghue, The timescale of early land plant evolution. *Proc. Natl. Acad. Sci. U.S.A.* **115**, E2274–E2283 (2018).

146. F. M. Cornejo-Castillo, A. M. Cabello, G. Szalasar, P. Sánchez-baracaldo, G. Lima-Mendez, P. Hingamp, A. Alberti, S. Sunagawa, P. Bork, C. de Vargas, J. Raes, C. Bowler, P. Wincker, J. P. Zehr, J. M. Gasol, R. Massana, S. G Acinas, Cyanobacterial symbionts diverged in the late Cretaceous towards lineage-specific nitrogen fixation factories in single-celled phytoplankton. *Nat. Commun.* **7**, 11071 (2016).

147. A. P. Sims, D. G. Mann, L. K. Medlin, Evolution of the diatoms: Insights from fossil, biological and molecular data. *Phycologia* **45**, 361–402 (2006).

148. A. Caputo, M. Stenegren, M. C. Pernice, R. A. Foster, A short comparison of two marine planktonic diazotrophic symbioses highlights an un-quantified disparity. *Front. Mar. Sci.* **5**, 10.3389/fmicb.2019.00045 (2018).

149. R. P. Anderson, C. R. Woltz, N. J. Tosca, S. M. Porter, D. E. G. Briggs, Fossilisation processes and our reading of animal antiquity. *Trends Ecol. Evol.* **38**, 1060–1071 (2023).
